# Next-generation broad-spectrum reactivators for effective countermeasure against organophosphorus poisoning

**DOI:** 10.1007/s00204-026-04341-y

**Published:** 2026-03-16

**Authors:** Jana Zdarova Karasova, Martina Hrabinova, Alzbeta Dlabkova, Vendula Hepnarova, Natalie Zivna, Monika Schmidt, Valeria Sheshko, Carilyn Torruellas, Jakub Opravil, Lukas Prchal, Natalie Vanova, Jakub Fibigar, Zbynek Vecera, Tomas Kucera, Jaroslav Chladek, Gabriele Horn, Franz Worek, Jan Marek, Jaroslav Pejchal, Daniel Jun, Ondrej Soukup, Jan Korabecny, Lukas Gorecki

**Affiliations:** 1 Department of Toxicology and Military Pharmacy, Military Faculty of Medicine, University of Defence, Trebesska 1575, 500 01 Hradec Kralove, Czech Republic; 2 Biomedical Research Centre, University Hospital Hradec Kralove, Sokolska 581, 500 05 Hradec Kralove, Czech Republic; 3 Department of Molecular Pathology and Biology, Military Faculty of Medicine, University of Defence, Trebesska 1575, 500 01 Hradec Kralove, Czech Republic; 4 U. S. Army CCDC Chemical Biological Center, Aberdeen Proving Ground, MD 21010-5424 USA; 5 Department of Military Medical Service Organization and Management, Military Faculty of Medicine, University of Defence, Trebesska 1575, 500 01 Hradec Kralove, Czech Republic; 6 Department of Pharmacology, Faculty of Medicine in Hradec Kralove, Charles University, Simkova 870, 500 03 Hradec Kralove, Czech Republic; 7 Bundeswehr Institute of Pharmacology and Toxicology, Neuherbergstrasse 11, 80937 Munich, Germany; 8 Department of Epidemiology, Military Faculty of Medicine, University of Defence, Trebesska 1575, 500 01 Hradec Kralove, Czech Republic

**Keywords:** Reactivator, Oxime, Acetylcholinesterase, Butyrylcholinesterase, Nerve agents, Pesticides

## Abstract

Sustained effort has been dedicated to the development of novel cholinesterase reactivators—the only causal antidotes—to counter organophosphorus (OP) intoxication. As more lethal nerve agents—such as A-agents—continue to emerge, the existing arsenal of causal antidotes remains unchanged. Approved oxime reactivators—**2-PAM**, **HI-6**, and **LüH-6**—are restricted by their limited efficacy spectrum, poor blood–brain barrier permeability, and suboptimal pharmacokinetics. The objective of this study is to design, synthesize, and characterize a new class of asymmetric monoquaternary bisoxime reactivators with broad-spectrum reactivation potential, favorable pharmacokinetics, and dual mechanisms of action—cholinesterase reactivation and direct OP compound degradation. In vitro and in vivo experiments identified **LG-1795** as the lead candidate with the broadest OP spectrum. The averaged second-order reactivation constant (*k*_*r2*_) across five *h*AChE–OP and two *h*BChE-OP complexes was 16.8 mM^−1^ min^−1^, surpassing clinical standards. Notably, **LG-1795** reactivated both AChE and BChE, a dual activity not previously reported for reactivators. In vivo, **LG-1795** restored both cholinesterase enzymes and demonstrated prophylactic efficacy against GB, VX, and PXE following intramuscular administration, preventing symptoms in sarin-poisoned animals. These findings represent a significant advance in antidotal therapy, providing the first evidence that asymmetric monoquaternary bisoximes deliver broad-spectrum efficacy against nerve agents and pesticides while simultaneously targeting both OP-inhibited cholinesterases. The translational potential of **LG-1795** supports its further preclinical development as a next-generation countermeasure for both clinical and regulatory use in chemical defense.

**Supplementary Information:**

The online version contains supplementary material available at 10.1007/s00204-026-04341-y.

## Introduction

**Fig. 1:**
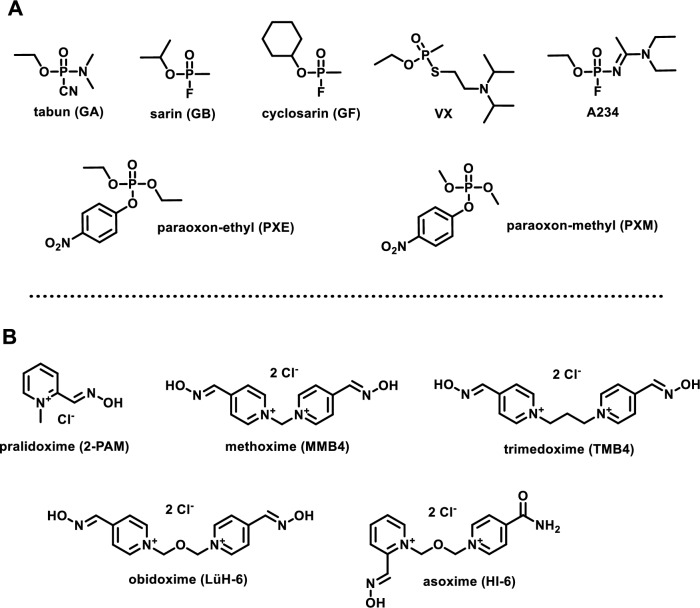
**A** Chemical structures of well-known NAs, including representatives of G-agents (GA, GB, GF), V-agents (VX), and A-agents (A234). Chemical structures of the two traditional OP insecticides, PXE and PXM, are also shown. **B** Chemical structures of the five clinical standards employed in antidotal programs worldwide: pralidoxime (**2-PAM**), methoxime (**MMB4**), trimedoxime (**TMB4**), obidoxime (**LüH-6**), and asoxime (**HI-6**)

**Fig. 2:**
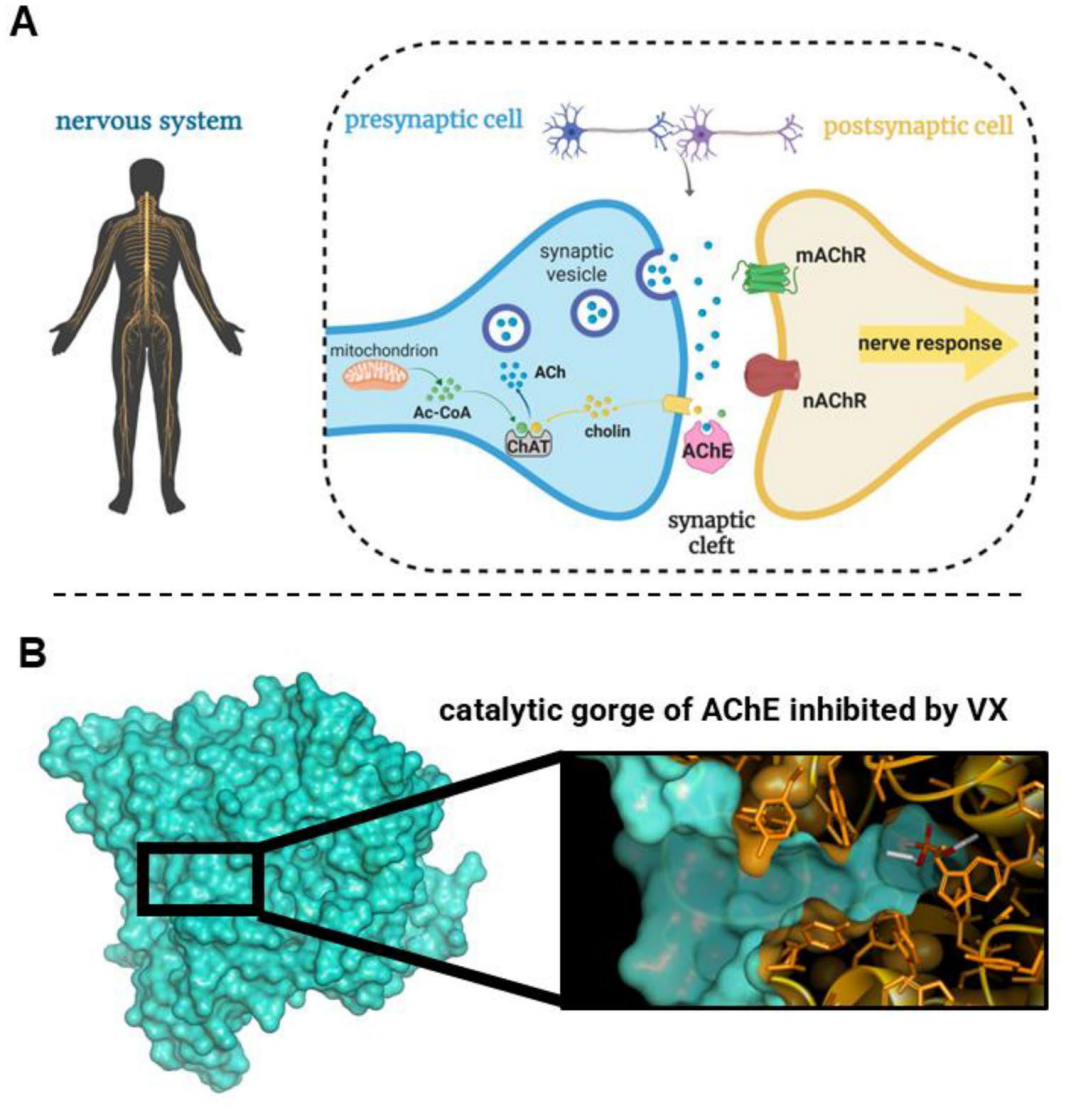
**A** Schematic representation of the cholinergic synapse. Acetylcholine (ACh) is synthesized in the presynaptic neuron from acetyl-CoA and choline by choline acetyltransferase (ChAT) and stored in synaptic vesicles. Upon neuronal stimulation, ACh is released into the synaptic cleft, where it activates muscarinic (mAChR) and nicotinic (nAChR) receptors on the postsynaptic membrane, eliciting a nerve response. AChE terminates the signal by hydrolyzing ACh into acetate and choline. **B** Molecular visualization of the catalytic gorge of AChE inhibited by VX. The surface rendering of the AChE protein (cyan) highlights the deep active-site gorge. The inset shows the bound VX molecule (dark orange), covalently linked to the active-site serine (Ser203), thereby preventing further hydrolysis of ACh and resulting in enzymatic inactivation

Exposure to organophosphorus (OP) compounds, including nerve agents (NAs) and OP insecticides (Fig. 1A), can precipitate a rapid progression toward potentially fatal outcomes. OP intoxication is attributed to the irreversible inhibition of acetylcholinesterase (AChE; E.C. 3.1.1.7) (Costanzi et al. 2018). The physiological role of AChE lies in the termination of neuronal impulses at cholinergic synapses by hydrolyzing the neurotransmitter acetylcholine (Fig. 2A). Inhibition of AChE leads to the accumulation of acetylcholine at synaptic junctions, causing prolonged stimulation of cholinergic receptors, culminating in a cascade of symptoms ranging from miosis and bronchorrhea to seizures, paralysis, and death (Trancart et al. 2025; Taylor 2017). OP compounds pose not only a public health hazard in agricultural settings but also a significant chemical warfare threat. Notable examples include the use of sarin during the Tokyo subway attack in 1995 (Okumura et al. 2007), large-scale chemical attacks in the Syrian civil war (2013 and 2017) (Rosman et al. 2014), and the more recent deployment of highly potent agents such as VX and A-agents in targeted assassination attempts (Paddock and Sang-Hun 2017; Steindl et al. 2021). Treatment for OP poisoning encompasses the use of a reactivator molecule to restore AChE function via nucleophilic attack on the phosphorus atom of the OP-AChE conjugate (Franjesevic et al. 2019). Symptomatic treatment, such as the administration of the antimuscarinic antagonist atropine (Connors et al. 2014) and anticonvulsants (Aroniadou-Anderjaska et al. 2016), may accompany causal treatment; however, complete recovery is only possible by restoring enzyme activity, either through reactivation or de novo enzyme synthesis. Currently, five clinical standards—pralidoxime (**2-PAM**), methoxime (**MMB4**), trimedoxime (**TMB4**), obidoxime (**LüH-6**), and asoxime (**HI-6**)—have been used in antidotal programs worldwide (Fig. 1B) (Worek et al. 2020). The presence of a permanently charged, pyridinium-based aldoxime moiety serves as a common structural feature among all these compounds. The quaternary nitrogen ensures high affinity toward the aromatic gorge of the inhibited enzyme, while the nucleophilic oxime group serves as the key reactive element in the reactivation process (Fig. 1B and 2B). However, this general pharmacophore presents several limitations, including poor blood–brain barrier (BBB) permeability and a narrow spectrum of efficacy against OPs (Worek et al. 2020). Efforts to overcome these limitations have been ongoing for years across the scientific community. A considerable number of novel reactivators with structural variability have been developed; however, none has advanced to clinical application (Taylor et al. 2019; Gorecki et al. 2022). Alongside AChE reactivation, increasing attention has been devoted to butyrylcholinesterase (BChE; EC 3.1.1.8), a member of the cholinesterase (ChE) family, that plays a secondary but significant protective role in OP intoxication (Lockridge 2015; Masson and Nachon 2017). BChE binds OP compounds in the bloodstream, thereby reducing the number of OP molecules reaching AChE and delaying or mitigating the severity of symptoms (Nichols and Chambers 2021). A notable example is the clinical case of A-234 poisoning involving the Russian activist Alexei Navalny, in which *i.v.* administration of BChE-containing fresh frozen plasma facilitated scavenging of the remaining free agent and led to increased plasmatic BChE activity (Steindl et al. 2021). These findings have consistently highlighted the therapeutic potential of BChE, particularly in the context of pseudo-catalytic bioscavenger strategies (Wille et al. 2017; Kohoutova et al. 2024). Building on this concept, we systematically evaluated whether our newly developed oximes could effectively reactivate both AChE and BChE, aiming to develop dual-action antidotes. To the best of our knowledge, no previous study has systematically assessed novel reactivators targeting the restoration of activity of both cholinesterases. This dual-targeting strategy represents a key innovation of our work.

**Fig. 3:**
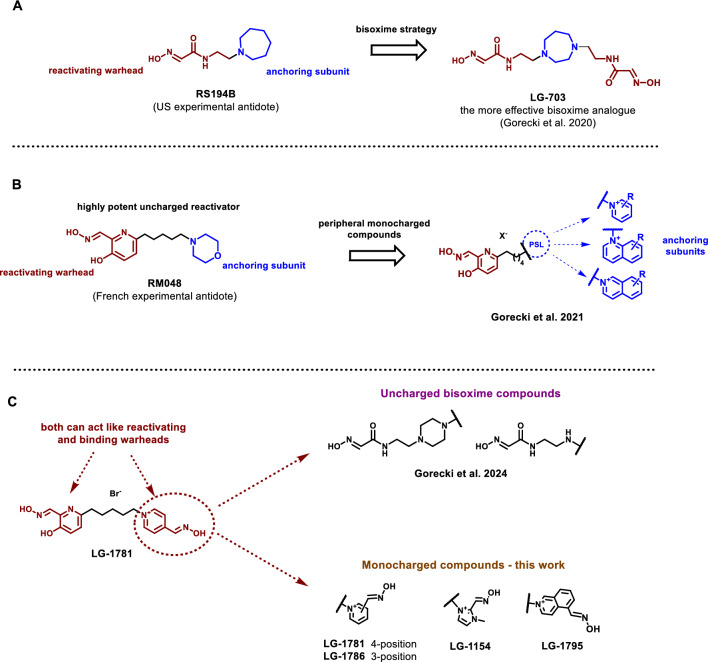
**A** Structural derivation of the initial uncharged bisoxime reactivator (LG-703) from a previously reported experimental antidote, RS194B. The design strategy was based on the dimerization of two pharmacophores via a protonatable saturated heterocyclic linker, resulting in an enhanced reactivation efficiency (Gorecki et al. 2020). **B** Structural concept inspired by the highly potent uncharged reactivator RM048, developed by a French research group (Zorbaz et al. 2018a, 2020). This molecule employs a 2-(hydroxyiminomethyl)pyridine-3-ol scaffold as a nucleophilic warhead, which was further expanded by introducing a permanently charged anchoring group targeting the peripheral site of the inhibited enzyme (Gorecki et al. 2021). **C** Overview of the current design strategy based on asymmetric bisoxime reactivators bearing two distinct nucleophilic warheads. Two structural categories are illustrated: uncharged bisoximes reported previously (Gorecki et al. 2024), and the novel series of monocharged analogues presented in this study, designed to enhance both reactivation efficacy and target engagement versatility

The design of the reactivator molecules described here was guided by two prior studies: one on monoquaternary reactivators bearing a permanent positive charge in the peripheral binding ligand, and another on asymmetric bisoxime reactivators (Fig. 3) (Gorecki et al. 2021, 2024). These concepts build on extensive efforts by research teams worldwide to improve reactivation efficacy and structural adaptability. Notably, a potent uncharged reactivating moiety—namely, the 2-(hydroxyiminomethyl)pyridine-3-ol scaffold—was introduced due to its high reactivation potential. This structural motif was first proposed over a decade ago by a French research group led by Florian Nachon and Pierre-Yves Renard (**RM048**, Fig. 3B) (Mercey et al. 2011; Saint-André et al. 2011). Recent work by a U.S. research group at the University of California, San Diego, inspired the bisoxime strategy by incorporating hydroxyiminoacetamide scaffolds into symmetric bisoxime frameworks (Fig. 3A) (Gorecki et al. 2020).

Prior work by Gorecki et al. (2021) demonstrated that the inclusion of a permanently charged moiety—presumed to interact with the enzyme’s peripheral site—conferred high affinity across a range of OP–AChE conjugates and provided significantly greater versatility than current clinical standards (Fig. 3B) (Gorecki et al. 2021). Subsequent work sought to enhance structural diversity through the integration of a second, spatially distinct reactivating moiety, leading to the development of asymmetric bisoximes bearing two nucleophilic warheads (Fig. 3C) (Gorecki et al. 2024). The combination of both design strategies—peripheral site-targeting positive charge and bifunctional oxime-based reactivator—was expected to improve efficacy and broaden the reactivation spectrum. These structural motifs are anticipated to optimize the molecular interactions within the active and peripheral sites, thereby enhancing the ability to accommodate and effectively reactivate OP-inhibited cholinesterases. To this end, an extensive literature survey was conducted to identify suitable charged nucleophilic motifs to be incorporated into the next-generation bisoxime scaffolds. Among the identified scaffolds, 1-methylimidazole-2-aldoxime possesses a notable affinity for *h*BChE and a considerable reactivating capacity against OP-inhibited enzymes (Sit et al. 2014; Wei et al. 2014). Isoquinoline-5-aldoxime was also selected as a promising nucleophilic motif due to its effective BChE reactivation potential and high affinity towards AChE (Malinak et al. 2020). The latter property is of particular interest, as quinolinium-based systems exhibit strong interactions within the AChE active site, suggesting their potential to enhance affinity for the enzyme’s peripheral site (Musilek et al. 2011; Hrabcova et al. 2017). Pyridinium-2-aldoxime, pyridinium-3-aldoxime, and pyridinium-4-aldoxime were also included. It has been well documented that the 2-positioned aldoxime group on the pyridine ring is superior for reactivation of soman or GB, while the 4-position is superior for reactivation of the GA-inhibited enzyme (Worek et al. 2004). While pyridinium-2-aldoxime is found in **2-PAM** and **HI-6**, **LüH-6** and **MMB4** are the clinical representatives bearing two pyridinium-4-aldoxime moieties. Several halogenated pyridinium-4-aldoximes were also examined due to their enhanced reactivation potency (Zorbaz et al. 2018b; Prchalova et al. 2024). However, their inclusion to the current design was ultimately precluded by low chemical accessibility and poor stability (Zorbaz et al. 2018c, 2022; Prchalova et al. 2024). Similarly, pyridinium-2-aldoxime derivatives encountered the same difficulties.

This work describes a new class of “next-generation” reactivators that integrate three advances into a single chemical architecture: (i) dual cholinesterase targeting (AChE and BChE); (ii) the combination of two chemically distinct oxime warheads within a single scaffold, designed to couple high nucleophilicity with stable, affinity-driven binding, thereby enabling the compounds to address a wider range of OP–cholinesterase adducts and achieve broad-spectrum reactivation potency; and (iii) a modified pharmacokinetic profile differentiated from classical bis-quaternary standards. By incorporating two distinct oximes, the nucleophilic attack trajectory at the phosphorus center is optimized, while the binding and orientation are stabilized through complementary peripheral interactions, such as aromatic stacking or cation–π. Importantly, these functions are not fixed: either oxime can serve as the primary nucleophile, while the other provides anchoring, maintaining productive binding orientations across structurally diverse complexes. This synergy lowers *K*_*D*_ and raises *k*_*r*_ simultaneously, thereby enhancing *k*_*r2*_ across chemically diverse OP–ChE adducts (Table 3). The asymmetric pairing was selected based on literature-reported motifs with documented AChE/BChE engagement and validated experimentally through a comprehensive biochemical evaluation against both AChE and BChE. The reactivator’s ability to directly interact with NAs under physiological conditions was also explored as a potential additive mechanism complementing enzymatic reactivation. Proof-of-concept in vivo studies in mouse models assessed toxicity, pharmacokinetics, and pharmacodynamics of the top-ranked candidates, while in silico molecular dynamics (MD) simulations provided theoretical support for the observed in vitro trends.

## Materials and methods

### General chemistry

Column chromatography was performed using silica gel 100 at atmospheric pressure (70–230 mesh ASTM, Merck, Prague, Czech Republic). Analytical thin-layer chromatography was carried out using plates coated with silica gel 60 featuring a fluorescent indicator F254 (Merck, Prague, Czech Republic). Thin-layer chromatography plates were visualized by exposure to ultraviolet light (254 nm) or using KMnO_4_, as a detection reagent. Chemicals were purchased from Sigma-Aldrich Co. LLC (Prague, Czech Republic), or Fluorochem Ltd. (Hadfield, United Kingdom) and were used without additional purification. CEM Explorer SP 12 S Class (CEM Corporation, Matthews, NC, USA) was used for microwave irradiation. All compounds active in biological assays were screened in silico for pan-assay interference compounds (PAINS), with no hits detected (Baell and Holloway 2010). MarvinSketch was used for drawing and visualizing chemical structures and reactions (Marvin 23.8, ChemAxon, https://www.chemaxon.com).

### Chemical characterization

NMR spectra were recorded on a Varian S500 (Varian, Inc., Palo Alto, CA, USA) and Bruker Avance Neo 500 (Bruker BioSpin GmbH, Rheinstetten, Germany) spectrometers (500 MHz for ^1^H, and 126 MHz for ^13^C). Chemical shifts are reported in δ ppm referenced to a solvent’s residual peaks, standards for ^1^H NMR and ^13^C NMR: CDCl_3_ (CHCl_3_-*d*_*1*_*;* 7.26 (H); 77.16 (C) ppm), CD_3_OD (CH_3_OH-*d*_*4*_; 3.35, 4.78 (H), 49.3 (C) ppm), or hexadeutero-dimethyl sulfoxide (DMSO-*d*_*6*_; 2.50 (H), 39.7 (C) ppm). See Supplementary information Sect. 8 and 9.

The compounds were analyzed by an HPLC Dionex Ultimate 3000 RS coupled with a Q Exactive Plus orbitrap mass spectrometer to obtain high-resolution mass spectrometry (HRMS) spectra (Thermo Fisher Scientific, Waltham, Massachusetts, USA). The samples were dissolved in methanol and/or DMSO/methanol solution 50/50 (v/v). For HRMS and purity analysis, a gradient method was used with reverse-phase C18 column (Kinetex EVO 2.1 × 50 mm, 1.7 μm, Phenomenex, Torrance, CA, USA) as the stationary phase. Purified water with 0.1% formic acid (mobile phase A) and acetonitrile with 0.1% formic acid (mobile phase B) were used as the mobile phases. The method started with 5% B for 0.3 min, the gradient then rose to 100% B in 3 min, remained stable at 100% B for 0.7 min, and then returned to 5% B and the column equilibrated for 3.5 min. The total runtime of the method was 7.5 min. The column temperature was maintained at 27 °C, the flow of the mobile phase was 0.4 mL/min, and the injection volume was 1 μL. To determine HRMS and purity in very polar compounds, a Hypersil GOLD Amino (2.1 × 100 mm, 1.9 µm, Thermo Fisher Scientific, Waltham, MA, USA) column was used. Mobile phase A was 10 mM ammonium acetate buffer (pH = 4), and mobile phase B was acetonitrile. The flow was constant at 0.35 ml/min. The method started with 0.5 min of the isocratic flow at 95% B, the gradient of B then decreased to 40% B in 7.5 min and remained constant at 40% B for 2 min. The composition was then returned to 95% B and equilibrated for 5 min. Total runtime was 15 min. The column was maintained at 35 °C, with a mobile phase flow of 0.35 mL/min. Sample injection was 1 µL. UV detection (diode array detector, 254 nm) confirmed ≥ 95% purity for all compounds. HRMS spectra were collected from the total ion current in positive mode in the scan range 105–1000 m/z, with the resolution set to 140,000. See Supplementary information Sect. 8 and 10.

### Experimental p***K***_a_ determination

Each p*K*_a_ measurement is based on the direct determination of the ratio between the neutral and ionized molecules. The protonation of ionizable groups in studied compounds was monitored using UV–visible spectrophotometry.

Twelve 20 mM phosphate-pyrophosphate buffers (pH range 5–13) were prepared according to Radic et al. (2012) (Radić et al. 2012). To a 900 µL buffer solution, 100 µL of 1 mM compound solution (typically in distilled water) was added. UV spectra for each pH point were recorded in triplicate over a wavelength range of 200 to 650 nm, using a Synergy 2 microplate reader (BioTek, Germany). Differences in maximal absorbance were used for p*K*_a_ determination. Absorbance values of the solvent were subtracted from the absorbance of the compound. p*K*_a_ values were calculated in GraphPad Prism5 by nonlinear regression.

### GB-surrogate and VX-surrogate degradation

A solution of GB-surrogate NIMP (nitrophenyl isopropyl methylphosphonate) or VX-surrogate NEMP (nitrophenyl ethyl methylphosphonate) (Chemforase, Mont Saint-Aignan Cedex, France) was diluted with DMSO to obtain a concentration of 10 mM. Reactivator stock solutions were diluted in Dulbecco’s Phosphate Buffered Saline (PBS, Merck, Darmstadt, Germany) at 1 mM (resulting in ratio 1:1.25, surrogate:oxime). A volume of 125 µL of each stock solution was mixed with 10 µL of 10 mM surrogate solution in a well of a 96-well plate. Each sample was measured in triplicate. The absorption changes were measured by a spectrophotometer (Synergy™ HT, BioTek Instruments, Vermont, USA) at 402 nm. The kinetic interval was set at 6 s between each reading, and data were collected for up to 60 min. Each compound was assayed in triplicate. The resulting data were processed using the spectrophotometer software (Gen5, version 2.01.14, BioTek Instruments, Vermont, USA) and then converted into Microsoft Excel. The rate constant was calculated using the procedure described by Guggenheim (Guggenheim 1926), with modifications introduced by Cabal et al. (Cabal et al. 2007). The methodology is based on the following accepted equation:

1 $${\mathrm{A}}_{t + \Delta t} = {\text{ A}}_{t} \times {\text{ e}}^{k\Delta t} + {\text{ A}}_{n} \times \, (1 \, - \, e^{ - k\Delta t} )$$

where: A_t+∆t_ = absorption at time t + ∆t; A_t_ = absorption at time t; e = base of natural logarithm; *k* = observed rate constant; ∆t = time between two successive measurements; and A_n_ = absorption at infinite time.

This relationship is a linear equation with slope e^*k*∆t^ and an intersection at A_n_ × (1—e^−*k*∆t^). Data from a ten-minute measurement were copied in two columns of MS Excel and the second column was shifted one row up. This shift presents one value ∆t. Data prepared in this way were displayed in an XY scatter chart. A trend line with regression equation and its coefficient was plotted through the indicated points. A trend line with the corresponding regression equation was overlaid on the displayed points. The obtained slope was used to calculate the rate constant *k* from the term e^*k*∆t^. Rate constants were recalculated to the reaction half-times. The resulting data were calculated as the average from three measurements.

### GB, VX, and A234 degradation

Stock solutions of GB (purchased from the Military Research Institute, Brno, Czech Republic, 50% purity, determined by GC–MS) at a concentration of 8 µmol/mL, VX (purchased from the Military Research Institute, Brno, Czech Republic, 43% purity, determined by GC–MS) at a concentration of 3 µmol/mL, and A234 (purchased from the Military Research Institute, Brno, Czech Republic, > 90% purity, determined by GC–MS) at a concentration of 1 µmol/mL, were prepared by dissolving the agent in anhydrous acetonitrile (GB and A234) or anhydrous propan-2-ol (VX), both from Merck (Darmstadt, Germany). The stock solutions of **LG-1781**, **LG-1786,** and **LG-1795** with a concentration of 30 µmol/mL were prepared in methanol (Hypergrade for LC–MS, Merck). The stock solutions of **LG-1154**, **HI-6**, **2-PAM,** and **LüH-6** at the same concentration of 30 µmol/mL were prepared in ultrapure water produced by Aqua Osmotic 06 (Aqua Osmotic, Tisnov, Czech Republic). PBS (phosphate buffered saline tablet, Sigma-Aldrich, St. Louis, USA), pH 7.4 was prepared from tablets according to the manufacturer’s instructions by dissolving one tablet in 200 mL of deionized water.

The volumes of oxime and NA stock solutions as listed in Table 1 were pipetted into a 10 mL volumetric flask and made up with PBS to obtain the working solutions of the oxime and NA in PBS.

Working solutions of GB and VX in the presence and absence of the oxime were incubated at laboratory temperature, and samples (0.5 mL) were collected at time intervals of 0, 15, 30, 60, 90, 120, 180, 240 and 300 min. 1 µL of 2-[(dimethylamino)methyl]phenol (2-DMAMP, Sigma–Aldrich, St. Louis, USA) was added. Each mixture was incubated with shaking at 25 °C for 15 min to generate stable nerve agent 2-DMAMP derivatives and then analyzed by liquid chromatography-mass spectrometry (LC–MS). The derivatization method was adopted from Blanca et al. (2020), (Blanca et al. 2020). The experiments were performed in triplicate for GB solutions and in two triplicates for VX solutions. The aliquots of A234 working solutions with and without oxime were incubated at 37 °C in the autosampler of the HPLC system for 5 h and injected directly for analysis every 15 min. As the concentration of A234 in the PBS solution was stable, the experiments were not repeated.

GB, VX, and A234 concentrations at all time intervals were quantified using high-performance liquid chromatography-mass spectrometry (HPLC–MS). HPLC analysis was performed using a Vanquish Core system (Thermo Scientific, San Jose, CA, USA) on a Gemini NX-C18 column (5 μm, 150 mm × 2.0 mm i.d.) preceded by a C18 Security Guard cartridge (4.0 mm × 3.0 mm i.d.), both from Phenomenex (Torrance, CA, USA). The mobile phase consisted of 10^−3^M ammonium formate in water (A) and 10^−3^M ammonium formate in methanol (B). The separation was performed by gradient elution with a flow rate of 1.0 mL/min and the following elution program: 0–0.3 min (5% B); 0.3–3.3 min (5% to 100% B); 3.3–4 min (100% B); 4–4.01 min (100% to 5% B); 4.01–10 min (5% B). The temperature of the column was set to 35 °C. The runtime of the analysis was 15 min. The mobile phase was introduced into the MS source by switching the diverter valve at 1.5 min. Mass spectrometry was performed on an LTQ XL linear ion trap instrument (Thermo Scientific, San Jose, CA, USA) with a heated electrospray ionization probe (HESI-II) operated in positive ion mode. The parameters in the source were set as follows: source voltage 4.5 kV, source heater temperature 370 °C, sheath gas flow 30 arbitrary units, and auxiliary gas flow 10 arbitrary units. The mass spectrometer was operated in the selected reaction monitoring mode. The observed transitions were *m/z* 272 → 230; 185 for GB-2-DMAMP derivative, *m/z* 258 → 230; 185 for VX-2-DMAMP derivative and *m/z* 225 → 197; 152 for A234.

**Table 1:** Preparation of 10 mL of working solutions of GB, VX, or A234 and selected oxime used to observe the ability of reactivators to directly decompose organophosphorus compounds

Nerve agent (NA)	NA: oxime ratio	NA stock solution volume [µL]	Oxime stock solution volume [µL]	NA final concentration in PBS [nmol/mL]	Oxime final concentration in PBS [nmol/mL]
GB	1:10	100	267	80	800
GB	1:5	100	133	80	400
GB	1:1	100	27	80	80
VX	1:10	50	5	1.5	15
A234	1:10	40	13.3	4	40

### LC analysis of compounds stability

The stability of reactivators at laboratory temperature was evaluated in PBS (Figure S24). All compounds were dissolved in DMSO, diluted with PBS (0.1 M, 25 °C, pH 7.4) to a final concentration of 10^–4^ M and vortexed prior to the injection in the HPLC system. The HPLC system, column and mobile phases used in this study are the same as with the C18 purity method mentioned above. The method began with a 2% B solution for 0.5 min, followed by a 2.5-min gradient rise to 100% B. It remained stable at 100% B for 0.5 min and then returned to 2% B, with the column equilibrated for an additional 2.2 min. The total runtime of the method was 5.2 min. The column temperature was maintained at 30 °C, the flow of the mobile phase was 0.4 mL/min, and the injection volume was 1 μL. The autosampler was set to 25 °C to incubate samples. The injection of each sample was determined immediately after dilution (time point 0) and then at 2-h time intervals up to 24 h. Determination of compounds was performed by MS. Data were collected in the positive mode in total ion current setting in the range of 105–1000 m/z. The concentration of reactivators in the solution was quantified by integrating the area under the curve of total ion chromatograms, selectively filtered for the exact mass of the target compound. Data were obtained from three independent experiments, and visualization was performed using GraphPad Prism version 6.05 (San Diego, USA).

### Production and purification of mouse and rat acetylcholinesterase

The nucleotide canonical sequences encoding mouse acetylcholinesterase (*m*AChE; P2183, isoform T) and rat acetylcholinesterase (*r*AChE; P37136, isoform T) were obtained from the GenBank database (NIH). The sequence for a His6-tag was introduced at the C-terminus of both genes. The resulting constructs were commercially synthesized with codon optimization for expression in Expi293™ cells (Invitrogen GeneArt services, Thermo Fisher Scientific, Prague, Czech Republic). They were subsequently cloned into pcDNA™3.4 TOPO® expression vector (Thermo Fisher Scientific, Prague, Czech Republic). Verified plasmids were transformed into NEB® 10-beta *E. coli* cells (New England Biolabs) and isolated using the PureLink® HiPure Plasmid Filter Midiprep Kit (Invitrogen, Thermo Fisher Scientific, Prague, Czech Republic). The ExpiFectamine™ 293 Transfection Kit (Thermo Fisher Scientific, Prague, Czech Republic) was used for transient transfection of plasmid DNA into Expi293. The cholinesterases were produced as secreted proteins for seven days at 37 °C, in an atmosphere of 8% CO_2_, with orbital shaking at an optimal spinner speed of approximately 125 rpm. At the end of the expression, the cell culture supernatant for AChE was collected and directly subjected to purification steps. Both ChEs were desalted using Amicon® Ultra 15 mL centrifugal devices with Ultracel® filters with a molecular weight (cut-off of 30,000 daltons; Merck) and subsequently purified using Ni–NTA affinity chromatography (GE Healthcare, Chicago, IL, USA). The proteins were eluted from the resin by a concentration gradient of 25–250 mM imidazole in 20 mM TRIS buffer (pH 7.5, 150 mM NaCl; Merck) followed by buffer exchange step using Amicon® Ultra 15 mL centrifugal devices (cut-off of 30,000 daltons (Merck, Prague, Czech Republic). The expression level of the proteins, their enzymatic activity, and the efficiency of the purification steps were checked using Ellman’s method (Ellman et al. 1961). The purity of the purified enzymes was checked by SDS-PAGE. The enzymes were stored at -80 °C for further use.

### In vitro reactivation screen

The reactivation potency of the standard and novel compounds was evaluated on human recombinant *h*AChE, *m*AChE, *r*AChE, and human recombinant *h*BChE. Enzymes were prepared at the Department of Toxicology and Military Pharmacy (Military Faculty of Medicine, University of Defence) according to previously established protocol (Hrabinova et al. 2024). The enzyme was inhibited by a solution of the appropriate cholinesterase inhibitor, such as GB, VX, or PXE, in propan-2-ol at a concentration of 10^–4^_,_ 10^–5^ and 10^–4^ M for 5 × half-life of inhibition (33, 45 and 45 min) at 37 °C. The excess of the inhibitor was subsequently removed using an octadecylsilane-bonded silica gel solid-phase extraction (SPE) cartridge (UCT, Bristol, PA, USA). The inhibited enzyme was incubated for 10 min with a solution of reactivator at a concentration of 10^–4^ M at 37 °C, 0.1 M phosphate buffer (pH 7.4), and DTNB (1 mM). The reaction was initiated by adding the substrate acetylthiocholine/butyrylthiocholine (ATCh/BTCh) (1 mM). The activities of *h*AChE/*m*AChE/*r*AChE/*h*BChE were then measured spectrophotometrically at 412 nm by the modified method according to Ellman (Ellman et al. 1961). Each concentration (10^–4^ and 10^–5^ M) of the reactivator was assayed in triplicate. The obtained data were used to compute reactivation potency (R; Eq. 2). The results were corrected for oximolysis and inhibition of *h*AChE/*m*AChE/*r*AChE/*h*BChE by the reactivator.

2 $$R = \left( {1 - \frac{{\Delta A_{0} - \Delta A_{r} }}{{\Delta A_{0} - \Delta A_{i} }}} \right) \times 100$$

where: ΔA_0_ = change in absorbance caused by intact cholinesterases (phosphate buffer instead of *h*AChE/*h*BChE inhibitor), ΔA_i_ = change in absorbance provided by *h*AChE/*m*AChE/*r*AChE/*h*BChE exposed to inhibitors, and ΔA_r_ = change in absorbance caused by *h*AChE/*m*AChE/*r*AChE/*h*BChE incubated with the solution of reactivator.

### In vitro reactivation kinetics

Hemoglobin-free erythrocyte ghosts served as the source of erythrocyte human AChE and were prepared as described previously (Worek et al. 2012). Prior to use, erythrocyte ghosts were thawed and homogenized on ice with a Sonoplus HD 2070 ultrasonic homogenizer (Bandelin Electronic, Berlin, Germany) twice for 5 s with a 20 s interval in between to achieve a homogenous matrix for kinetics studies. Human heparinised plasma served as the source for human BChE. OP-inhibited AChE was prepared by adding a small volume (< 1% v/v) of tabun, sarin, cyclosarin, VX (purity > 95% by GC–MS, ^1^H NMR, and ^31^P NMR; German Ministry of Defence, Bonn, Germany) or PXE (supplied by Dr Ehrenstorfer, Augsburg, Germany) to erythrocyte ghosts, and incubating the mixture for 15 min at 37 °C to achieve > 95% inhibition of control activity. OP-inhibited BChE was prepared by adding a small volume (< 1% v/v) sarin or VX to human plasma and incubating the mixture for 15 min at 37 °C to achieve > 95% inhibition of control activity. Excess OP was removed through dialysis of the inhibited samples in phosphate buffer (0.1 M, pH 7.4, Merck, Prague, Czech Republic) overnight at 4 °C. The residual enzyme activity was measured by incubating OP-treated and control enzymes (30 min, 37 °C). Aliquots were stored at − 80 °C. 150 µL of OP-treated AChE or BChE were mixed with an equal volume of phosphate buffer (0.1 M, pH 7.4 containing 0.2% gelatin for stabilization of cholinesterases; Merck), and 3 µL of obidoxime dichloride (Ferak, Berlin, Germany), asoxime dichloride (a gift from Dr Clement, Defence Research Establishment Suffield, Ralston, Canada) and LG-oximes in final concentrations of 1 to 100 µM were then added to initiate reactivation at t = 0. After the specified time intervals (t = 1 to 12 min), aliquots were transferred to cuvettes containing phosphate buffer, DTNB (0.3 mM; Sigma-Aldrich, Taufkirchen, Germany), and ATCh (0.45 mM, Merck; for AChE assay) or BTCh (1 mM, Merck; for BChE assay) for measurement of enzyme activity at 412 nm for 3 min (Worek et al. 1999). All experiments were performed in duplicate.

The pseudo-first-order reactivation rate constants (*k*_*obs*_) were determined via linear regression analysis, and the dissociation constant *K*_*D*_ and the reactivity constant *k*_*r*_ were calculated from plots of oxime concentration versus *k*_obs_ via nonlinear regression analysis using Prism 5.04 (GraphPad Software, San Diego, CA, USA) (Worek et al. 2004).

### Molecular dynamics

Molecular docking was employed to predict the binding poses of eight selected compounds and three commercial oxime reactivators (**LüH-6**, **2-PAM**, and **HI-6**) into the active site of *h*AChE inhibited by either GB or VX. The 3D structures of all compounds were generated using Open Babel (O’Boyle et al. 2011) and optimized using the general AMBER force field (GAFF) with Avogadro (Hanwell et al. 2012). Molecular modeling was performed using the oximate form of the reactivators, representing the nucleophilically active species involved in the reactivation process. Although protonated oxime species are expected to be present at physiological pH based on experimental p*K*_a_ values, protonation–deprotonation equilibria were not explicitly modeled.

The structure of GB-inhibited *h*AChE was obtained from the RCSB Protein Data Bank (PDB ID: 6WV1, resolution 2.37 Å); the VX-inhibited structure was also retrieved from the RCSB database (PDB ID: 6CQW, resolution 2.28 Å) (Berman 2000; Berman et al. 2003; Bester et al. 2018a, b; McGuire et al. 2021a, b; Burley et al. 2025; Bester, S.M., Guelta, M.A., Pegan, S.D., Height, J.J.; McGuire, J.R., Bester, S.M., Pegan, S.D., Height, J.J.). Only the protein chain and covalently bound inhibitor were preserved for both proteins. Protein structures were prepared using the DockPrep function in UCSF Chimera (Pettersen et al. 2004), converted to the PDBQT format with OpenBabel (v3.1.0), and then to GRO format using GROMACS (Abraham et al. 2024) with the Amber14SB force field and TIP3P water model (Maier et al. 2015).

Semi-flexible molecular docking was performed in triplicate using AutoDock Vina (Eberhardt et al. 2021). The top-ranked docking poses from all the replicates were selected for molecular dynamics (MD) simulations. Ligand parameters were generated using Antechamber (v22.0) with GAFF (Wang et al. 2004, 2006). Each protein–ligand complex was solvated in TIP3P water and neutralized with Na^+^ and Cl^−^ ions During equilibration, positional restraints (1000 kJ·mol^−1^·nm^−2^) were applied only to the protein and the covalently bound organophosphorus inhibitor, while all water molecules and ions were left fully unrestrained to allow proper solvent relaxation. No positional restraints were applied during the production MD simulations. Energy minimization was followed by equilibration: 100 ps in the NVT ensemble at 300 K and 100 ps in the NPT ensemble at 1 bar. Subsequently, 100 ns production MD simulations were performed. All trajectories were inspected and final structures were visualized using PyMOL Molecular Graphics System (Schrödinger, LLC, 2015). Furthermore, an analysis of interaction energies was performed, calculated as the sum of short-range Lennard–Jones and Coulombic interaction energies. Simulations exhibiting large ligand displacements or the absence of a stable RMSD plateau were not used for quantitative interaction energy calculations, as such trajectories do not provide meaningful time-averaged energetic descriptors. These trajectories were not considered informative for productive active-site binding and were excluded solely to avoid introducing noise into the energy analysis. The excluded trajectories did not contradict the binding trends or qualitative conclusions derived from the retained simulations. In total, 132 independent 100-ns molecular dynamics simulations were performed, corresponding to 1–5 simulations per complex (average 3.6 simulations per complex). Trajectories were screened for stability prior to interaction energies analysis. A total of 48 simulations were excluded based on RMSD-based criteria. Specifically, 21 trajectories exceeded an average heavy-atom RMSD least-squares fitted to backbone threshold of 1.5 nm or a RMSD standard deviation of 0.5 nm. An additional 27 trajectories were excluded following visual inspection of RMSD time profiles, where no stable plateau indicative of a persistent binding mode was observed. RMSD thresholds were selected based on systematic inspection of ligand behavior within the active site across all trajectories. Statistical analysis was performed using JASP (v0.19.1; (JASP Team 2024)).

### Animal models for in vivo studies

Young adult Balb/c mice (both sexes, 20–30 g) were obtained from Velaz, Únětice, Czech Republic. Animals were housed in an air-conditioned facility under a 12 h light/dark cycle (lights on 07:00 a.m.–07:00 p.m.) with free access to standard chow (Cerea Corporation, Pardubice, Czech Republic) and tap water. The vivarium accreditation number was 69233/2015-MZE-17214 (Military Faculty of Medicine, University of Defence, Czech Republic). All experimental procedures were approved by the Ethics Committee of the Military Faculty of Medicine (protocols MO-106085/2023-1457, MO-192365/2024-1457, and MO-474883/2024-1457) and were conducted in accordance with national and institutional ethical guidelines.

Animals were acclimatized for at least 10 days prior to experimentation. They were assigned to groups of four (acute toxicity, pharmacokinetics), five (overall protective index), or eight (reactivation studies). Test solutions (10% Kolliphor® in PBS, v/v) were freshly prepared and administered within 30 min of preparation.

### In vivo toxicity study

Assessment of acute toxicity was conducted through the determination of the maximum tolerated dose (MTD). The experimental conditions included monitoring for toxicity signs, performing autopsy with macroscopic examination, and conducting histopathological analysis of organs.

Balb/c mice were randomly assigned to experimental groups, **LG-1154**, **LG-1781**, **LG-1786**, and **LG-1795**, with four males and four females in each group per administered dose. The control group also consisted of four males and four females. Test compounds and sham control treatments were both administered intramuscularly (10% Kolliphor® in PBS, V/V, 0.1 mL/10 g). Multiple doses were administered to determine the MTD. Subsequently, animals were monitored for signs of toxicity, including changes in cardiovascular, respiratory, and nervous systems. Weight loss and reduced food consumption were also recorded. According to the FELASA classification, observations were conducted during the first 2 h and then periodically over 48 h. The classification of toxicity signs and the subsequent determination of the MTD followed the methodology described (Misik et al. 2018).

All animals surviving the 48-h observation period were euthanized using CO_2_ and subjected to basic macroscopic necropsy. Liver, kidney, and muscle samples were collected for standard histopathological examination. Organs were fixed in 10% neutral-buffered formalin (Bamed, Ceske Budejovice, Czech Republic), processed histologically, and stained with hematoxylin and eosin (both Merck, Prague, Czech Republic) (Pejchal et al. 2012). Histopathological changes were evaluated using a BX-51 microscope (Olympus, Tokyo, Japan).

### Pharmacokinetic study

Based on the acute toxicity study results, oximes **LG-1781**, **LG-1786**, and **LG-1795** were selected for further in vivo studies. Selected oximes were dissolved in 10% Kolliphor® in PBS and administered at 25 mg/kg (**LG-1781** and **LG-1786**) and 28.8 mg/kg (**LG-1795**) based on their respective MTDs. The doses were adjusted to 50% of the MTD for **LG-1786**, with equimolar recalculation (intramuscular—*i.m.* administration; 0.1 mL/10 g). Blood and brain samples were collected at 10 time points (pre-dose, 3, 5, 10, 15, 20, 30, 60, 120, and 240 min), with 4 animals per time point. Animals were exsanguinated following transcardial perfusion, and plasma and brain samples were subsequently collected as described (Zdarova Karasova et al. 2010; Wu et al. 2021). Whole blood samples were centrifuged at 3,000 × g for 10 min at 4 °C, and plasma samples were stored at -80 °C until analysis. Oxime concentrations in all samples were quantified using HPLC–MS. All treated animals exhibited normal behavior without any side effects throughout the experiment. Non-compartmental analysis of the concentration–time curves was performed with the help of the Kinetica software (version 4.0, Kinetica, Arlington, USA). In addition, the absorption rate constant *k*_a_ was estimated using the method of residuals, assuming a one-compartment disposition model with first-order absorption and elimination. The absorption half-life was calculated as follows: t_1/2abs_ = ln(2)/*k*_a_.

### HPLC–MS for LG-compounds

The brains were weighed and added to PBS (from tablets, Sigma-Aldrich, Steinheim, Germany) at a ratio of 1:4 (w/w). Then the brains were homogenized using a T-25 Ultra Turrax disperser (IKA, Staufen, Germany). The homogenate was transferred to a microcentrifuge tube, sonicated by UP 50H needle homogenizer (Hielscher, Teltow, Germany), and the final homogenate was stored at − 80 °C prior to extraction.

A volume of 190 µL of brain homogenate or 95 µL of plasma was spiked with either 10 µL (brain homogenate) or 5 µL (plasma) of internal standard (IS; donepezil for LG compounds, TCI, Tokyo, Japan, and our laboratory IS K032 in methanol), with the final concentration being 100 nM. The sample was then vortexed and precipitated with 600 µL (brain homogenate) or 300 µL (plasma) of acetone (for HPLC, J.T.Baker, VWR, Fontenay-sous-Bois, France). The samples were shaken for 5 min (1800 RPM, VM-10 orbital motion Vortex Mixer, witeg Labortechnik GmbH, Wertheim, Germany) and then centrifuged (15,610 g, 5 min, Universal 320 R centrifuge, Hettich, Tuttlingen, Germany). 500 µL (brain homogenate) or 250 µL (plasma) of supernatant were transferred to a microtube and evaporated to dryness in a CentriVap concentrator (Labconco Corporation, Kansas City, USA). Before analysis, the samples were reconstituted in 100 µL of 50% (v/v) acetonitrile. Calibration samples were prepared by spiking 180 µL (brain homogenate) or 90 µL (plasma) of blank brain homogenate or plasma with 10 µL (brain homogenate) or 5 µL (plasma) of analyte (compounds **LG-1781**, **LG-1786**, **LG-1795**, **LüH-6**, or **HI-6**) methanol solution (final concentrations range from 0.5 nM to 5 µM) and 10 µL (brain homogenate) or 5 µL (plasma) of IS (final concentration 100 nM), and were then vortexed and extracted as above.

An HPLC–MS analysis was performed to determine the concentration of compounds **LG-1781**, **LG-1786**, **LG-1795**, **LüH-6**, or **HI-6** in plasma and brain homogenates. The system used in this study was Dionex Ultimate 3000 UHPLC: RS LPG quaternary pump, RS column compartment, RS autosampler, diode array detector, Chromeleon software (version 7.2.9 build 11,323, Thermo Fisher Scientific, Germering, Germany) with a Q Exactive Plus Orbitrap mass spectrometer with Thermo Xcalibur software (version 4.3.73.11, Thermo Fisher Scientific, Bremen, Germany). Detection was carried out by mass spectrometry in positive mode. Settings of the heated electrospray source were spray voltage 3.5 kV, capillary temperature: 262 °C, sheath gas: 50 arbitrary units, auxiliary gas: 12.5 arbitrary units, spare gas: 2.5 arbitrary units, probe heater temperature: 300 °C, max spray current: 100 µA, S-lens RF level: 50.

Data for synthesized compounds were obtained through reverse phase gradient elution method using a C18 column (Kinetex EVO C18 2.1 × 50 mm, 1.7 µm, Phenomenex, Torrance, CA, USA) with mobile phase A: 0.1% (v/v) formic acid in ultrapure water of ASTM type I (resistance 18.2 MΩ.cm at 25 °C) prepared using a Barnstead Smart2Pure 3 UV/UF apparatus (Thermo Fisher Scientific, Bremen, Germany) and mobile phase B: 0.1% (v/v) formic acid in LC–MS grade acetonitrile. The flow was constant at 0.45 mL/min. The method started with 0.5 min of isocratic flow at 3% B, the gradient of B then rose to 40% B in 2.4 min, and then rose to 100% after 0.7 min and remained constant at 100% B for 0.4 min. The composition was returned to 3% B and equilibrated for 3.5 min. The total runtime was 7.5 min. The column was tempered to 40 °C. The injection volume was 5 µL.

To assess the concentration levels of **LüH-6** and **HI-6**, the hydrophilic interaction chromatography (HILIC) approach was used. The method was employed as a gradient using an NH_2_ column (Hypersil GOLD Amino, 2.1 × 100 mm, 1.9 µm, Thermo Fisher Scientific, Waltham, MA, USA) as stationary phase and 20 mM ammonium acetate buffer (pH 4, Mobile Phase A) and acetonitrile (Mobile Phase B). The flow was kept constant at 0.35 mL/min. The method started with 0.5 min of the isocratic flow of 95% B, and then the gradient of B decreased to 40% B in 3.5 min and remained constant at 40% B for 1.5 min. The composition then returned to 95% B in 0.5 min and equilibrated for 4 min. The total runtime was 10 min. The column was tempered to 35 °C. The sample injection volume was 1 µL.

The concentration of compounds was measured using a mass spectrometer in positive mode, operating in total ion current mode. Searched masses and retention times (t_R_) are listed in Table 2. The calibration curve for plasmatic and brain homogenate levels of **LG-1781**, **LG-1786**, **LG-1795**, **LüH-6**, and **HI-6** had 9 points ranging from 0.5 nM to 5 µM.

**Table 2:** Searched masses and retention times of LG-1781, LG-1786, LG-1795, LüH-6, HI-6, and corresponding internal standards (IS)

	Compound	t_R_	Mass searched (m/z)
C18 analysis	LG-1781	2.50	165.08–165.09
	LG-1786	2.50	165.08–165.09
	LG-1795	3.00	190.090–190.105
	donepezil (IS)	3.61	380.22–380.23
NH_2_ analysis	LüH-6	4.95	144.0590–144.0615
	HI-6	4.95	144.0590–144.0615
	K032 (IS)	4.90	171.1015–171.1030

t_R_, retention time

### In vivo reactivation study against GB, VX, and PXE

Based on the in vitro results mentioned above, the therapeutic effectiveness of the new oximes was tested against GB, VX, and PXE. **HI-6**, **LüH-6**, and **2-PAM**, commonly used reactivators, served as reference standards. All reactivation and protective index studies followed the same protocol and dosing regimen.

OPs were administered *i.m* at 1 × LD_50_ (GA 229 µg/kg, VX 20 µg/kg, PXE 609 µg/kg; volume 0.1 mL/kg*.*). Animals were treated with either atropine alone (10 mg/kg, Merck) or a combination of atropine and a selected oxime. The treatment was administered *i.m.* one minute after nerve agent exposure at equimolar doses, recalculated to 50% of the MTD for **LG-1781** and **LG-1786** (both 25 mg/kg), **LG-1795** (28.8 mg/kg), **LüH-6** and **HI-6** (both 21.9 mg/kg), and **2-PAM** (10.4 mg/kg). Blood samples were collected under deep terminal anesthesia by cardiac puncture into heparinized 5 mL tubes at 60 min (n = 8). For brain tissue, animals were perfused transcardially with saline solution (0.9% NaCl) for 5 min; the skull was then opened, and the whole brain carefully removed and stored at − 80 °C until analysis.

Freshly-collected whole blood was hemolyzed using 20 mM TRIS buffer (pH 7.6, V_blood/_V_TRIS_ = 1/20) after 1 h. *m*AChE activity was measured spectrophotometrically at 436 nm using a Genesys BioMate160 spectrophotometer (Thermo Fisher Scientific, Prague, Czech Republic) by a modified Ellman’s method with ATCh (Merck, Prague, Czech Republic) as the substrate (Worek et al. 1999; Karasova et al. 2017). Each sample was analyzed in triplicate.

The remaining whole blood samples were centrifuged at 3,000 × g for 10 min at 4 °C, and plasma samples were stored at ‒80 °C until analysis. *m*BChE activity was measured directly at 412 nm using a Genesys BioMate160 spectrophotometer with BTCh (Merck, Prague, Czech Republic) as the substrate (Karasova et al. 2017). Each sample was analyzed in triplicate.

Brain samples were homogenized in 20 mM TRIS buffer (pH 7.6; V_brain/_V_TRIS_ = 1/10) using an Ultra-Turrax T 18 Digital homogenizer (Thermo Fisher Scientific, Prague, Czech Republic). Enzyme activity was measured as described above at the same time interval as bloods, with absorbance recorded at 412 nm (Ellman et al. 1961). Each brain sample was analyzed in triplicate.

ChE activity was calculated from absorbance values using a calibration curve with cysteine and expressed as μkat/kg or μkat/L (i.e., μmol of substrate hydrolyzed per kg of wet tissue per second or per liter of blood per second). The blood and brain ChE activity values of the control group were obtained from rats administered physiological saline (B. Braun Melsungen AG, Melsungen, Germany) instead of the nerve agent.

Normality was assessed using the Shapiro–Wilk test. Statistical analysis was performed using a two-way analysis of variance (ANOVA) or the Kruskal–Wallis test, both followed by Dunnett’s post *hoc* multiple comparisons test, using GraphPad Prism version 9.5.1 (GraphPad Software, Boston, USA).

### The protective ratio of LG-1781 and LG-1795 against GB, VX, and PXE

Based on the results of the reactivation study, the protective index was evaluated for all selected AChE inhibitors. Among the new candidates, only **LG-1781** and **LG-1795** were chosen for further studies. The dosing and administration protocol followed the same procedure as in the reactivation studies. NAs were administered at a dose of 1 × LD_50_ (GB 229 µg/kg, VX 20 µg/kg, PXE 609 µg/kg; volume: 0.1 mL/kg; *i.m.*). Treatment consisted of atropine (10 mg/kg) alone or in combination with oximes: LG-1781 (25 mg/kg), LG-1795 (28.8 mg/kg), LüH-6 (21.9 mg/kg), and HI-6 (21.9 mg/kg), administered *i.m*. 1 min after NA exposure.

OP-induced toxicity was assessed by determining the LD_50_ value and 95% confidence interval (CI) using probit regression analysis (SPSS Statistics version 26, IBM, Armonk, NY, USA). Mortality was recorded over 24 h following administration at three to five different doses, with five animals per dose (Tallarida and Murray 2012). The efficacy of the antidote/treatment was expressed as a protective ratio (LD_50_ value of protected mice/LD_50_ value of unprotected mice). Differences between LD_50_ values were analyzed using chi-squared parallelism of regression lines and relative median potency (RMP) with 95% confidence limits (SPSS Statistics version 26). Due to software limitations, only two samples could be tested simultaneously. The two samples were considered significantly different if the 95% confidence limits did not include 1.0 (Lei and Sun 2018).

### Functional observational battery

As in the protective ratio study, oximes **LG-1781** and **LG-1795** were tested using the Functional Observational Battery (FOB). The dosing and administration protocol remained consistent with the reactivation and protective ratio studies. NAs were administered at a dose of 1 × LD_50_ (GB: 196 µg/kg, VX: 20 µg/kg, PXE: 562 µg/kg; volume: 0.1 ml/kg; *i.m.*). Treatment (either atropine alone or combined with an oxime) was administered intramuscularly one minute after nerve agent exposure.

The potential neuroprotective effect was assessed using FOB, which includes evaluations of spontaneous behavior, motor activity, coordination, sensory responses, reflexes, and autonomic functions, as well as physical observations such as posture, skin condition, and respiratory patterns. Body weight was also recorded. The studies were blinded to ensure that the observer was unaware of the experimental design. FOB data included both categorical (scored) and continuous values. Scored values were analyzed using the chi-squared test of homogeneity, the concordance–discordance test, and the Kruskal–Wallis test. Continuous data were statistically evaluated, including the confidence interval (CI) for delta, Bartlett’s test for equality of variance, Williams’ test, and distribution function tests. Differences were considered statistically significant at p < 0.05, using GraphPad Prism version 9.5.1 (GraphPad Software, Boston, USA).

## Results

### Chemical synthesis

**Scheme 1:**
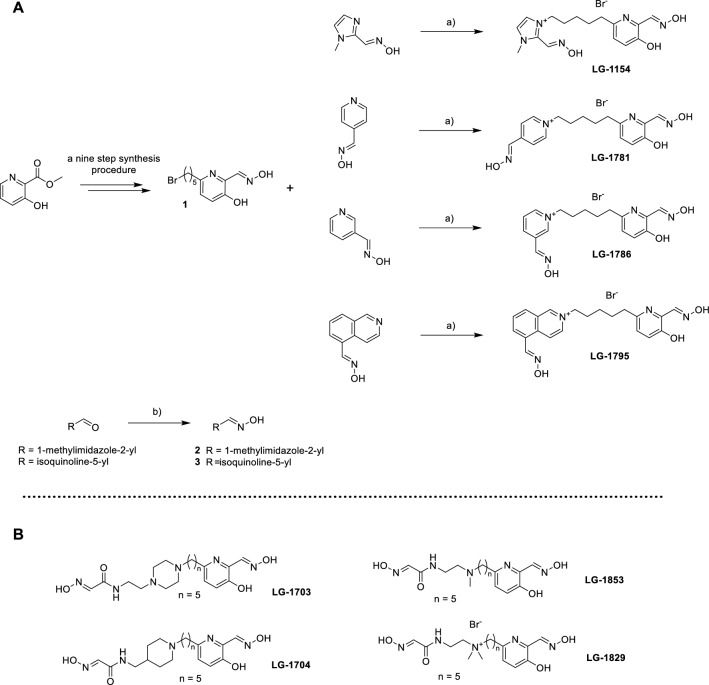
**A** Preparation of asymmetric monoquaternary bisoximes via MW-assisted *N*-alkylation. A nine-step synthetic route to intermediate **1** has been reported previously (Gorecki et al. 2021, 2024). Reagents and conditions: a) MW, MeCN, 24 h, 90 °C; b) NH_2_OH (50% in H_2_O), absolute EtOH, RT, 1–24 h. **B** Four previously reported reference bisoxime reactivators (Gorecki et al. 2024) that were used for comparative purposes in this study

Intermediate **1** was synthesized via a nine-step pathway, which proved pivotal to the overall synthetic strategy (Scheme 1) (Gorecki et al. 2021, 2024). Aldoxime fragments were either obtained commercially or synthesized via condensation of the corresponding aldehydes with hydroxylamine. Finally, microwave (MW) assisted *N*-alkylation provided the desired monoquaternary bisoximes. This reaction represents the limiting step for the synthetic feasibility of various proposed reactivators. Intermediate **1** carries a nucleophilic nitrogen on the pyridine ring, with a predicted p*K*_a_ value of 3.21, indicating its susceptibility to *N*-alkylation. In comparison, the nitrogen in pyridine-3-aldoxime has a calculated pKa of 4.31, rendering it more than ten times more susceptible to *N*-alkylation. In contrast, pyridine-2-aldoxime, with a p*K*_a_ of 2.78, is less prone to alkylation and more likely to undergo intramolecular cyclization or other side reactions. Consequently, the pyridinium-2-aldoxime analogue among others—such as halogenated pyridinium-4-aldoximes with nitrogen p*K*_a_ < 2.0—were not included in this study. This work led to the synthesis of four novel asymmetric monoquaternary bisoximes, which were investigated in detail. The novel compounds were characterized using ^1^H and ^13^C NMR spectroscopy, HRMS, and LC–MS, which confirmed their structures and ensured a purity of ≥ 95%, as determined by UV analysis.

### In vitro reactivation screen using various enzymes

**Fig. 4:**
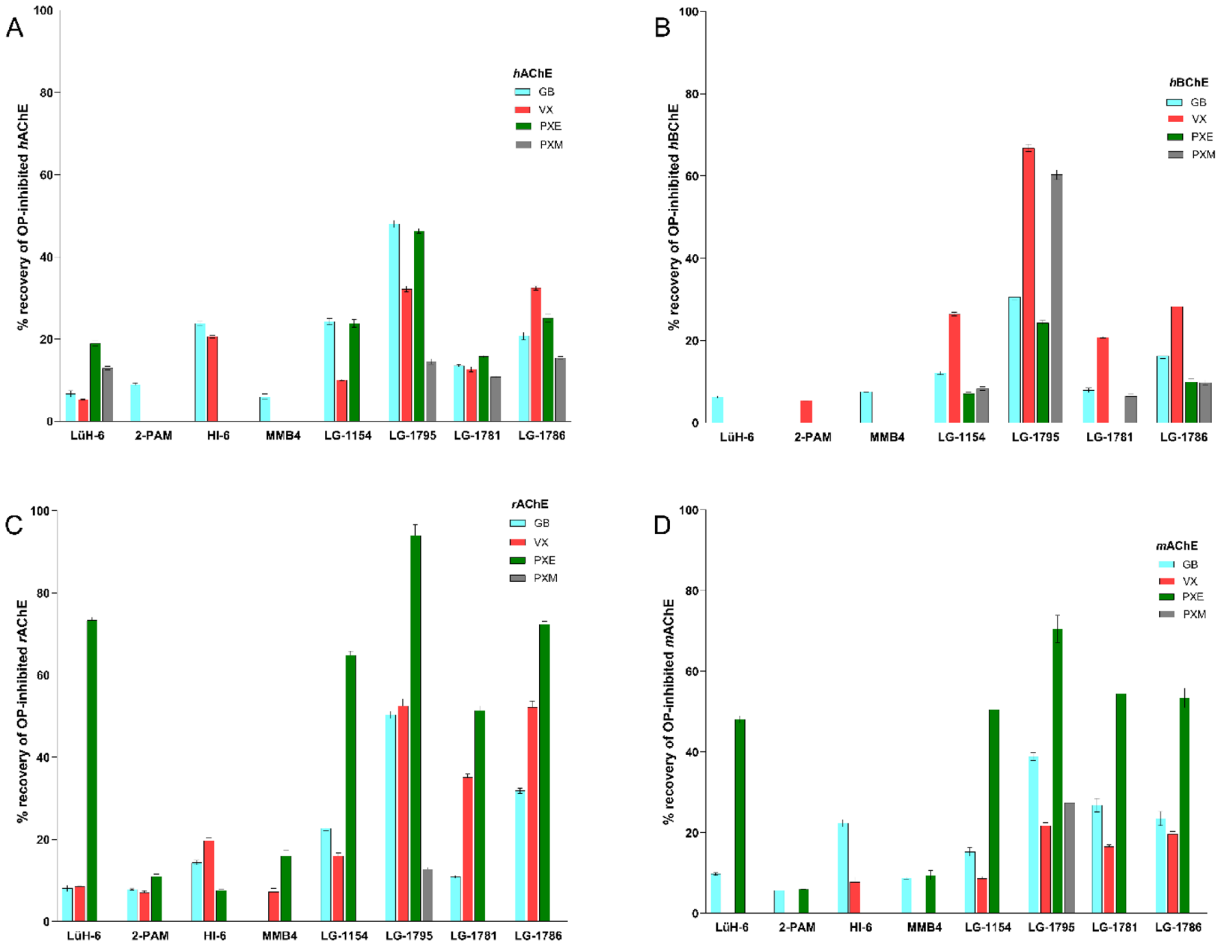
Reactivation efficacy of selected reactivators (10 µM) against OP-inhibited cholinesterases. Bar charts represent enzyme activity recovery (%) after 10 min incubation with GB-, VX-, PXE-, and PXM-inhibited *h*AChE **A**, *m*AChE **B**, *r*AChE **C**, and *h*BChE **D**. The results are expressed as the means of at least three experiments. The data can be found in SI Tables S1–S4

To comprehensively assess the reactivation efficacy of selected oximes, in vitro reactivation screening was performed using OP-inhibited ChE enzymes at a therapeutically relevant concentration of 10 µM (Fig. 4). The dataset for the higher 100 µM concentration is provided in Supplementary Tables S1–S4 and was not included in the main analysis. A 10-min incubation interval was chosen, as clinically useful oximes must act rapidly to ensure a favorable prognosis, making early time points particularly relevant. Four novel entities described here for the first time (**LG-1154**, **LG-1781**, **LG-1786**, and **LG-1795**; Scheme 1A), and four previously reported reference compounds (**LG-1703, LG-1704, LG-1853**, and **LG-1829**; Scheme 1B), were evaluated (Gorecki et al. 2024). Five OP inhibitors were selected from NA (GB, GA, VX) and OP pesticide groups (PXE and PXM). The reactivation potential was assessed across four species-relevant cholinesterases, human AChE (*h*AChE), mouse AChE (*m*AChE), rat AChE (*r*AChE), and human BChE (*h*BChE), to better predict translational efficacy. Due to the low reactivation potency for GA, the data are available in Supplementary Information only (Tables S1–S4).

Among all tested compounds, **LG-1795** proved to be the most potent broad-spectrum reactivator. **LG-1795** showed substantial reactivation of *h*AChE across all relevant OPs, with enzyme recovery reaching 48% for GB, 32% for VX, 46% for PXE, and 15% for PXM. In contrast, standard oximes such as **HI-6**, **2-PAM**, and **LüH-6** generally exhibited a reactivation potential below 20% against most agents, except for **HI-6** (24% for GB, 21% for VX) and **LüH-6** (19% for PXE). Notably, **LG-1786** and **LG-1154** also exhibited robust reactivation of GB-inhibited *h*AChE (21% and 24%, respectively), though their performance against other OPs was variable. When comparing the reactivation profiles across species, distinct differences were observed (Worek et al. 2002). However, conclusive interpretation of species-dependent reactivation efficacy remains elusive, as the reactivation profiles exhibited substantial variability contingent upon both the chemical nature of the oxime and the structure of the OP inhibitor. This disparity highlights the importance of carefully interpreting animal-to-human translational efficacy, especially in the context of novel molecular scaffolds.

Selected compounds were also evaluated against OP-inhibited *h*BChE, which functions as a systemic bioscavenger. Again, **LG-1795** was distinguished by its high efficacy, reaching 31% reactivation for GB and 67% for VX, significantly outperforming all clinical comparators. **LG-1786** also reached 28% reactivation for VX, whereas standard oximes (HI-6, LüH-6, 2-PAM) remained below the 10% threshold. These findings reinforce the therapeutic potential of **LG-1795** not only as a direct AChE reactivator, but also as a dually functional agent capable of enhancing peripheral detoxification via* h*BChE-OP reactivation.

### In vitro reactivation kinetics

To further elucidate the mechanistic performance of the evaluated oximes, detailed reactivation kinetics were assessed against *h*AChE inhibited by five OPs (GB, GF, GA, VX, and PXE), as well as *h*BChE inhibited by GB and VX (Tables 3, 4). In addition to the four newly synthesized candidates and clinical standards (**2-PAM**, **HI-6**, and **LüH-6**), a selection of literature-reported oximes with confirmed in vivo efficacy or advanced mechanistic profiles was also included: **RS194B**, an uncharged oxime with demonstrated efficacy in non-human primates (Rosenberg et al. 2017, 2024); **LG-703**, a bisoxime analogue of **RS194B** with superior in vitro properties (Gorecki et al. 2020); and **RM048** and **GM415**, two uncharged reactivators featuring a 3-hydroxypyridinium-2-aldoxime scaffold (Zorbaz et al. 2018a, 2020). The analysis of the three kinetic parameters, first-order cleavage rate constant (*k*_*r*_; min^−1^), dissociation constant (*K*_*D*_; µM)), and bimolecular reactivation rate constant (*k*_*r₂*_ = *k*_*r*_/*K*_*D*_; mM^−1^ min^−1^), enabled a quantitative comparison of reactivation efficiency across various OP-inhibited *h*AChE complexes. Among the tested compounds, **LG-1795** exhibited a highly favorable kinetic profile, particularly in terms of *k*_*r₂*_, which integrates both affinity and catalytic efficiency. Against GB-inhibited *h*AChE, **LG-1795** achieved a *k*_*r₂*_ of 9.3 mM^−1^ min^−1^, comparable to **HI-6** (13.5) and 2-PAM (9.1), though remaining below **LüH-6** (31.0). While **LüH-6** exhibited the highest absolute *k*_*r₂*_, **LG-1795** showed superior affinity (*K*_*D*_ = 13 µM) and a balanced *k*_*r*_ value. For VX, **LG-1795** exhibited a *k*_*r₂*_ of 29.0, which was superior to **HI-6** (21.0) and comparable to **LüH-6** (33.0). Notably, for PXE, while most reactivators demonstrated limited efficacy, **LG-1795** still attained the highest *k*_*r₂*_ among all tested compounds at 36.0, exceeding both **HI-6** (0.4) and **LüH-6** (25.0). Against GA- and GF-inhibited *h*AChE, **LG-1795** displayed lower *k*_*r₂*_ values (0.91 and 2.3, respectively), which—while moderate—were on a par with or superior to several reference reactivators. For GA, the standard reactivators **LüH-6**, **2-PAM**, **GM415**, and **RM048** exhibited *k*_*r2*_ values of 0.41, 0.01, 0.10, and 0.05, respectively, while **HI-6** showed no measurable activity. For GF, only **HI-6** surpassed **LG-1795** with a *k*_*r2*_ of 28.0, whereas other comparators, including **LüH-6** (0.42), **2-PAM** (0.06), **GM415** (0.20), **RM048** (0.32), **LG-703** (0.17), and **RS194B** (0.14), remained markedly less effective. Notably, **LG-1154** exhibited the lowest *K*_*D*_ values among all novel candidates, suggesting high binding affinity to the OP–AChE adducts. However, its comparatively low *k*_*r*_ values resulted in suboptimal* k*_*r2*_ rates, indicating that binding alone was not sufficient for efficient catalysis. The only exception was GF, where a balanced combination of *k*_*r*_ and *K*_*D*_ values resulted in an extraordinary *k*_*r2*_ rate of 94, exceeding that of **HI-6** by more than threefold (28 mM^−1^min^−1^). This contrast highlights the importance of a balanced kinetic profile, where both substrate affinity and cleavage efficiency contribute to optimal reactivation.

**Table 3:** Reactivation kinetics of the four novel reactivators, clinical standards and selected reactivators from the literature measured for GB-, GF-, GA-, VX- and PXE-inhibited *h*AChE

Compound	Reactivation kinetics of OP-inhibited *h*AChE ^1^														
	GB			GF			GA			VX			PXE		
	*k*_*r*_ (min^−1^)	*K*_*D*_ (µM)	*k*_*r2*_ (mM^−1^ min^−1^)	*k*_*r*_ (min^−1^)	*K*_*D*_ (µM)	*k*_*r2*_ (mM^−1^ min^−1^)	*k*_*r*_ (min^−1^)	*K*_*D*_ (µM)	*k*_*r2*_ (mM^−1^ min^−1^)	*k*_*r*_ (min^−1^)	*K*_*D*_ (µM)	*k*_*r2*_ (mM^−1^ min^−1^)	*k*_*r*_ (min^−1^)	*K*_*D*_ (µM)	*k*_*r2*_ (mM^−1^ min^−1^)
LG-1703 ^2^	0.25	104	2.4	0.16	336	0.5	0.021	58	0.36	0.53	85	6.2	0.29	48	6
LG-1704 ^2^	0.16	62	2.6	0.11	211	0.52	0.036	151	0.25	0.33	62	5.4	0.29	39	7.8
LG-1829 ^2^	0.11	86	1.2	0.06	191	0.35	0.026	173	0.15	0.13	34	3.7	0.4	139	2.9
LG-1853 ^2^	0.08	45	1.8	0.08	172	0.47	0.025	85	0.3	0.5	63	8	0.31	61	5.1
LG-1154	0.05 ± 0.003	14 ± 2.3	3.7 ± 0.8	0.69 ± 0.05	7.4 ± 1.0	94 ± 6.1	0.009 ± 0.000	6.4 ± 0.4	1.34 ± 0.14	0.05 ± 0.003	5 ± 0.3	10 ± 0.1	0.12 ± 0.01	5.5 ± 0.1	22 ± 1.4
LG-1781	0.11 ± 0.001	32 ± 8.2	3.8 ± 0.9	0.17 ± 0.02	270 ± 29	0.64 ± 0.00	0.021 ± 0.002	56 ± 1.9	0.37 ± 0.02	0.34 ± 0.01	42 ± 0.1	8.1 ± 0.2	0.33 ± 0.01	32 ± 3.5	10 ± 0.7
LG-1786	0.27 ± 0.003	86 ± 5.5	3.2 ± 0.2	0.14 ± 0.004	123 ± 15	1.1 ± 0.1	0.041 ± 0.001	83 ± 2.3	0.49 ± 0.03	0.32 ± 0.002	23 ± 1.9	14 ± 1.1	0.37 ± 0.002	27 ± 3.2	14 ± 1.7
LG-1795	0.12 ± 0.001	13 ± 1.6	9.3 ± 1.3	0.07 ± 0.001	31 ± 3.6	2.3 ± 0.3	0.021 ± 0.001	24 ± 1.6	0.91 ± 0.11	0.33 ± 0.025	11 ± 0.7	29 ± 0.4	0.29 ± 0.01	8.2 ± 1.5	36 ± 5.2
2-PAM ^3^	0.25	28	9.1	0.18	3159	0.06	0.01	706	0.01	0.22	28	7.7	0.17	187	0.9
LüH-6 ^3^	0.97	31	31	0.4	946	0.42	0.04	97	0.41	0.89	27	33	0.81	32	25
HI-6 ^3^	0.68	50	13.5	1.3	47	28	no activity	no activity	no activity	0.24	12	21	0.2	548	0.4
RS194B ^4^	0.60	1000	0.6	0.17	1300	0.14	no data found	no data found	no data found	0.60	530	1.1	0.08	970	0.1
LG-703 ^4^	0.80	900	0.9	0.50	2900	0.17	no data found	no data found	no data found	1.30	1200	1.1	0.14	2000	0.1
RM048 ^5^	0.12	220	0.6	0.04	120	0.32	0.014	310	0.05	0.57	120	4.9	0.27	620	0.4
GM415 ^5^	0.18	130	1.4	0.02	120	0.20	0.014	140	0.10	0.31	10	31	0.24	190	1.3

^1^*K*_*D*_ indicates affinity towards OP-AChE conjugate—low values mean higher affinities; *k*_*r*_ indicates ability to cleave OP-ChE covalent bond, higher is greater; and bimolecular rate constant *k*_*r2*_ indicates better efficacy with the higher values. Data of measured bisoximes **LG-1154**, **LG-1781**, **LG-1786**, and **LG-1795** are expressed as means ± SD; ^2^data taken from (Gorecki et al. 2024); ^3^ data taken from (Gorecki et al. 2021); ^4^ data taken from (Gorecki et al. 2020); ^5^ data taken from (Zorbaz et al. 2018a)

**Table 4:** Reactivation kinetics of the four novel reactivators, clinical standards and selected reactivators from the literature measured for GB- and VX-inhibited *h*BChE

Compound	Reactivation kinetics of OP-inhibited *h*BChE ^1^					
	GB			VX		
	*k*_*r*_ (min^−1^)	*K*_*D*_ (µM)	*k*_*r2*_ (mM^−1^ min^−1^)	*k*_*r*_ (min^−1^)	*K*_*D*_ (µM)	*k*_*r2*_ (mM^−1^ min^−1^)
LG-1154	0.10 ± 0.01	29 ± 4.1	3.3 ± 0.3	0.10 ± 0.00	18 ± 0.2	5.6 ± 0.1
LG-1781	0.22 ± 0.02	105 ± 17	2.2 ± 0.2	0.19 ± 0.01	42 ± 5.8	4.6 ± 0.4
LG-1786	0.20 ± 0.02	107 ± 31	2.0 ± 0.4	0.19 ± 0.01	35 ± 0.1	5.6 ± 0.1
LG-1795	0.04 ± 0.00	7 ± 1.0	5.7 ± 0.8	0.15 ± 0.01	4 ± 0.5	34.8 ± 1.2
2-PAM ^2^	0.28	579	0.4	0.28	759	0.4
LüH-6 ^2^	0.25	1017	0.3	0.21	409	0.5
HI-6 ^2^	0.73	2657	0.3	0.20	952	0.2
RM048 ^4^	0.004	–	–	0.06	160	0.4
GM415 ^4^	0.007	–	–	0.10	130	0.8

^1^ *K*_*D*_ indicates affinity towards OP-AChE conjugate—low values mean higher affinities; *k*_*r*_ indicates ability to cleave OP-ChE covalent bond, higher is greater; and bimolecular rate constant *k*_*r2*_ indicates better efficacy with the higher values. Data of measured bisoximes **LG-1154**, **LG-1781**, **LG-1786**, and **LG-1795** are expressed as means ± SD. ^2^ data taken from (Aurbek et al. 2009); ^4^ data taken from (Zorbaz et al. 2018a), in the case of GB, the *k*_*r*_ denotes the observed first-order reactivation rate constant, *k*_*obs*_ (min^−1^) at a 0.2 mM concentration

In addition to *h*AChE, selected compounds were evaluated for their ability to reactivate *h*BChE. **LG-1795** again emerged as the most efficacious novel oxime, achieving *k*_*r2*_ values of 5.7 mM^−1^ min^−1^ for GB and 34.8 for VX. In contrast, standard oximes exhibited limited activity: **HI-6**, **LüH-6**, and **2-PAM** remained below 1 mM^−1^ min^−1^ for both agents. These findings confirm the hypothesis—initially suggested by the reactivation screening—that **LG-1795** may exert dual pharmacodynamic effects by directly reactivating AChE and simultaneously supporting pseudo-catalytic detoxification via BChE. This dual action could be particularly advantageous in systemic circulation, where peripheral detoxification mechanisms are essential.

When averaged across all five *h*AChE–OP and two *h*BChE-OP complexes, the mean *k*_*r2*_ values were 16.8 mM^−1^min^−1^ for **LG-1795**, 12.9 mM^−1^min^−1^ for **LüH-6**, and 10.6 mM^−1^min^−1^ **HI-6**, supporting the potential of **LG-1795** as one of the most mechanistically efficient oxime reactivators currently available.

### In vitro reactivation of the agent A234

**LG-1795** was also examined for its ability to cleave the A234-AChE conjugate. Phosphoramidate-type NAs, such as GA and A234, are known to form notably stable OP-AChE conjugates that are difficult to reactivate due to the presence of a phosphorus–nitrogen (P–N) bond. The electron-donating nature of the nitrogen substituent stabilizes the partial positive charge on the phosphorus atom, reducing the electrophilicity of the OP-AChE complex and rendering it particularly resistant to reactivation with oximes (Worek et al. 2004; Carletti et al. 2008). The nitrogen atom in GA originates from a simple dimethylamide group, whereas in A234, it is incorporated into a more complex amidine moiety (see Fig. 1A and Table 5). Although GA and A234 differ in their leaving groups —cyanide and fluoride, respectively—this distinction appears to have a negligible impact on the reactivation potential once the covalent AChE adduct is formed. The markedly increased resistance to reactivation of A234-conjugated AChE is attributed to the steric and electronic effects of its amidine substituent, which introduces greater spatial hindrance and enhances stability of the conjugate, as previously reported (Hrabinova et al. 2024). However, GA and A234 diverged significantly in their time-course reactivation profiles. Whereas A234-inhibited *h*AChE remained only marginally responsive to **LG-1795**, GA-inhibited *h*AChE showed pronounced time-dependent recovery, with **LG-1795** becoming the most effective reactivator at 12 h (Table 5). This limited effect against A234 represents the clear exception within an otherwise broad-spectrum reactivation profile. This indicates that phosphoramidates are not uniformly refractory, and that the identity of the P–N substituent (dimethylamide vs amidine)—rather than the mere presence of the P–N bond – critically modulates reactivation kinetics.

**Table 5:** Comparison of in vitro reactivation rates of GA- and A234-inhibited *h*AChE at 100 µM concentration

	% Recovery of OP-inhibited *h*AChE at 100 µM of reactivator					
		
Compound	After 10 min	After 1 h	After 12 h	After 10 min	After 1 h	After 12 h
2-PAM	< 3	< 3	6.5 ± 0.5	< 3	< 3	< 3
LüH6	5.3 ± 0.3	11 ± 1.6	46 ± 1.9	< 3	3.0 ± 0.6	10 ± 1.5
HI-6	< 3	< 3	< 3	< 3	5.5 ± 0.9	23 ± 0.9
MMB4	< 3	< 3	4.5 ± 0.4	< 3	8.7 ± 1.2	51 ± 2.5
LG-1795	7.2 ± 0.8	18 ± 0.7	74 ± 1.0	< 3	< 3	6.7 ± 1.8

*h*AChE activity was measured after 10 min, 1 h, and 12 h of incubation of GA-*h*AChE or A234-*h*AChE with the compound. The results are expressed as means of at least three experiments. The chemical structures of GA and A234 were included for comparison

### Predictive molecular dynamics of reactivator binding and orientation

MDs were used to explain the reactivation potency of GB-inhibited and VX-inhibited *h*AChE with selected oxime reactivators. Simulations with unstable root-mean-square deviation (RMSD) as validation parameters were excluded (see Supplemental information, Figures S1-S22). Ligands were simulated in both binding orientations, defined by the proximity of either end to the GB or VX conjugate. The quaternary moiety was designated as '**A**', while the non-quaternary end was labeled '**B**', as illustrated in the top-scoring MD snapshots shown in Figs. 5 and 6. **LG-1781** was selected as a representative compound due to its incorporation of the pyridinium-4-aldoxime motif, also present in **LüH-6**. The MD parameters of interactions between the oxime and enzyme active sites, inhibited by GB or VX, were compared (Table 6; the detailed dataset containing all the parameters can be found in the SI, Tables S5 and S6).

**Fig. 5:**
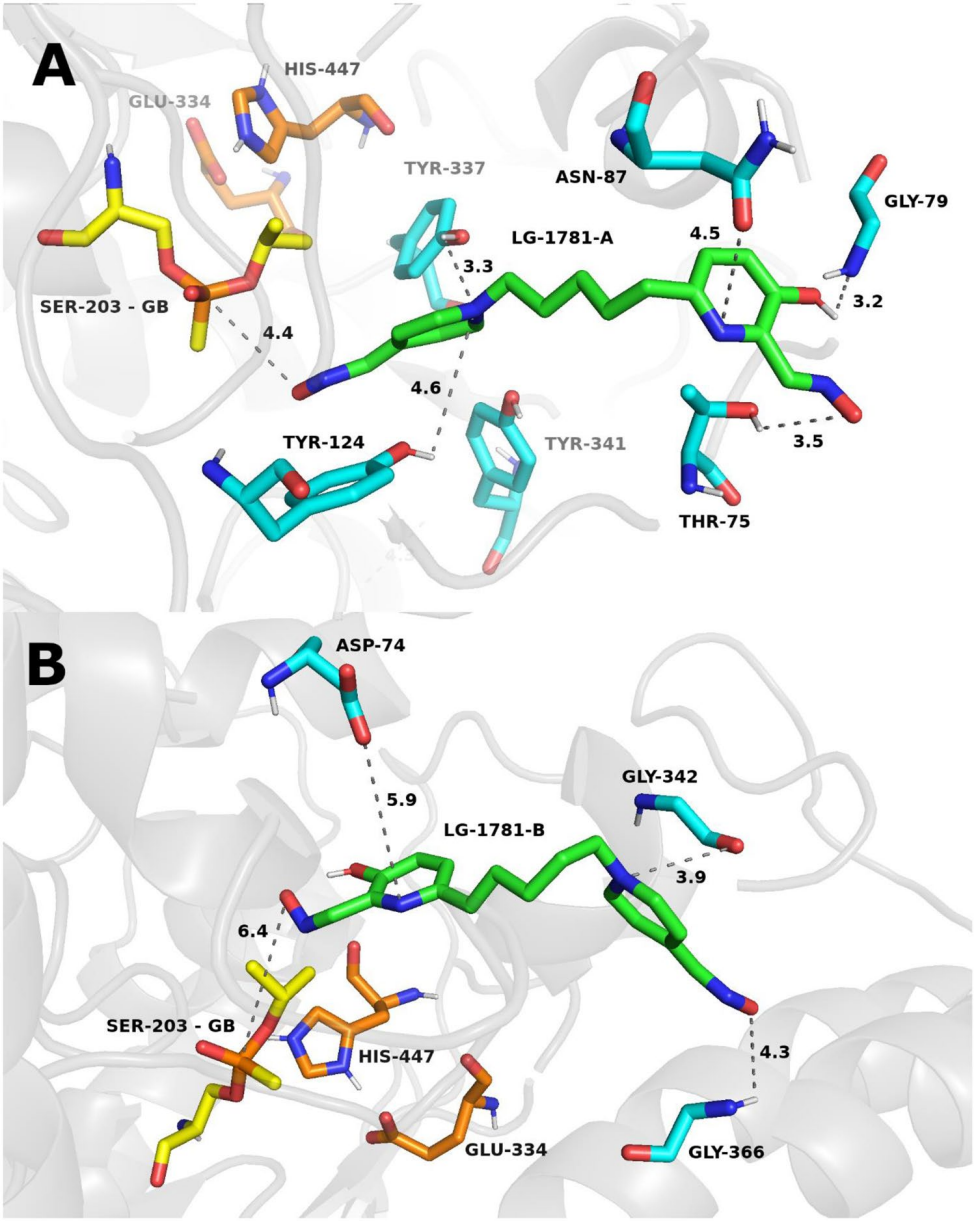
Representative top-scored MD snapshots illustrating the binding of oxime reactivator **LG-1781** to GB-inhibited *h*AChE. **LG-1781** is shown with green carbon atoms. In panel A, the quaternary moiety of **LG-1781** is positioned proximally to the phosphorus atom of GB, whereas in panel B, the non-quaternary moiety is oriented closer to GB. Catalytic triad residues His447 and Glu334 are shown in orange, while the active-site Ser203 inhibited by GB is highlighted in yellow. Amino acid residues interacting with the ligand are depicted in cyan. Distances shown by grey dashed lines are given in angstroms (Å)

**Fig. 6:**
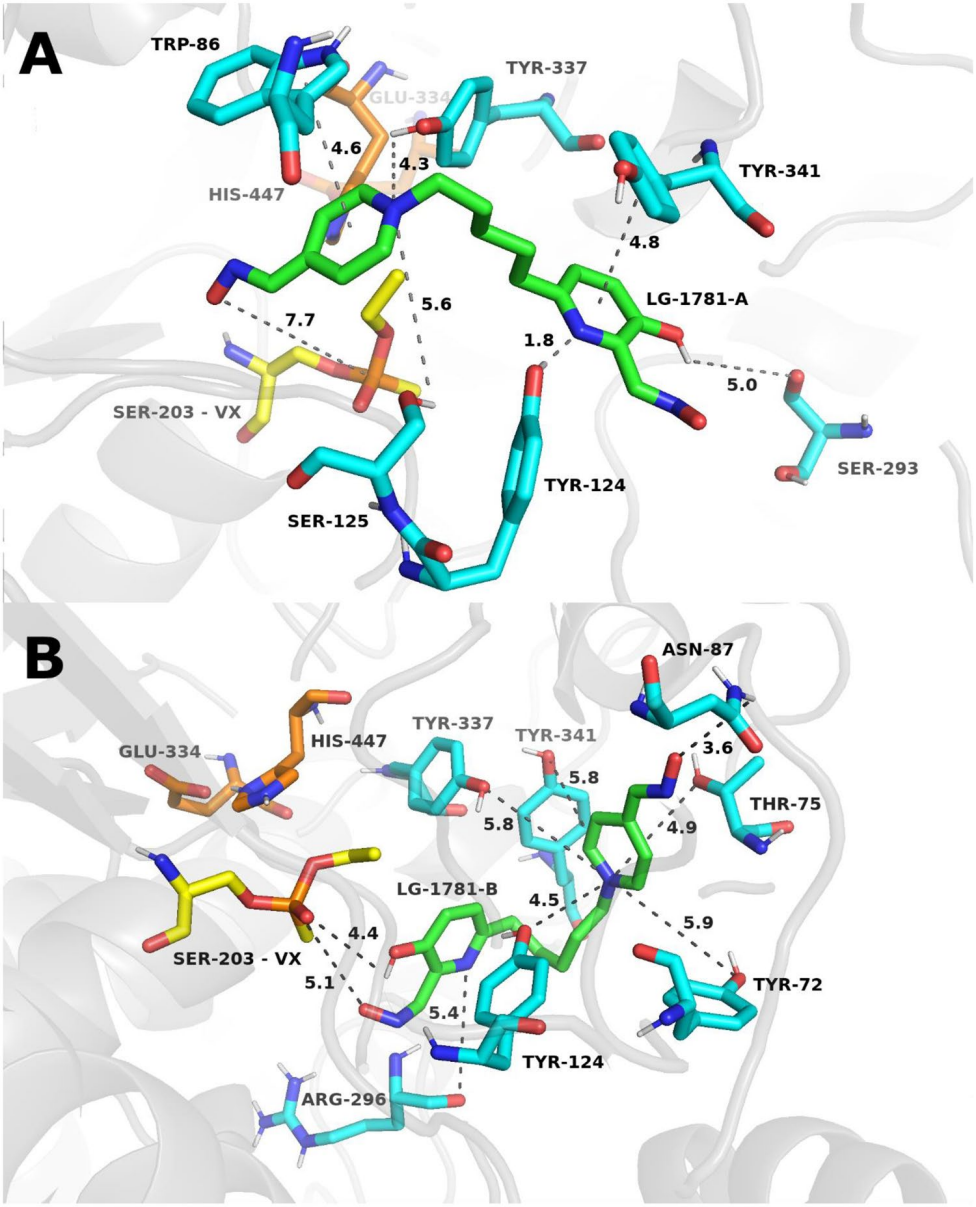
Representative top-scoring MD snapshots illustrating the binding of oxisme reactivator **LG-1781** to VX-inhibited *h*AChE. **LG-1781** is shown with green carbon atoms. In panel A, the quaternary moiety of **LG-1781** is positioned proximally to the phosphorus atom of the VX adduct. In panel B, the non-quaternary moiety is oriented closer to the VX-modified site. Catalytic triad residues His447 and Glu334 are shown in orange, and the active-site serine (Ser203), covalently modified by VX, is highlighted in yellow. Amino acid residues interacting with the ligand are depicted in cyan. Distances indicated by grey dashed lines are expressed in angstroms (Å)

**Table 6:** MD results summarizing each ligand orientation, with average (mean ± SD) O-P distances between the oximate oxygen and the phosphorus atom of GB or VX

Ligand	Orientation mark closer to OP	GB		VX	
		Average O-P distance [Å]	Interaction energy between the oxime and GB [kJ/mol]	Average O-P distance [Å]	Interaction energy between the oxime and VX [kJ/mol]
LüH-6	A	9.2 ± 0.2 9.5 ± 0.1	−6.1 ± 1	9.6 ± 2.3 14.3 ± 2.2	−3.2 ± 0.4
2-PAM	A	8.7 ± 2.5	−1.9 ± 1.2	7.4 ± 0.8	−4.8 ± 3.6
HI-6	A	9.2 ± 2.8	−5.0 ± 1.1	9.8 ± 2.1	−2.5 ± 0.2
LG-1154	A	11.3 ± 2.8 14.9 ± 0.4	−3.0 ± 1.5	7.5 ± 1.9 14.8 ± 2.6	−4.7 ± 0.4
	B	11.9 ± 0.2 10.6 ± 1.3	−3.2 ± 0.9	16.8 ± 2.0 10.2 ± 3.6	−0.03 ± 0.53
LG-1795	A	8.7 ± 0.8 11 ± 1.0	−12.4 ± 0.7	13.5 ± 3.3 17.2 ± 2.0	−0.5 ± 0.2
	B	14.5 ± 3.2 11.2 ± 1.2	−6.8 ± 1.4	20.3 ± 4.4 10.7 ± 3.0	−0.9 ± 0.6
LG-1781	A	7.4 ± 1.5 16.8 ± 2.1	−7.7 ± 0.9	8.8 ± 1.8 16.5 ± 3.0	−4.1 ± 0.6
	B	13.8 ± 2.7 12.7 ± 4.7	−2.8 ± 0.7	15.7 ± 4.1 5.2 ± 0.5	−7.6 ± 0.4
LG-1786	A	8.6 ± 1.8 14.3 ± 1.0	−6.6 ± 0.5	7.2 ± 1.9 11.2 ± 0.9	−10 ± 0.7
	B	13.9 ± 2.0 10.4 ± 2.9	−4.2 ± 0.6	13.7 ± 2.3 10 ± 1.3	−6.2 ± 0.8
LG-1703	A	14.9 ± 4.8 16.1 ± 3.9	−2.0 ± 1.5	8 ± 2.2 11.6 ± 1.6	−3.7 ± 0.6
	B	21.7 ± 3.1 7.1 ± 2.0	−1.0 ± 0.6	20.3 ± 1.8 5.3 ± 1.8	−5.7 ± 0.5
LG-1704	A	no simulation fulfilled RMSD stability criteria		13 ± 4.8 13.6 ± 2.9	−4.0 ± 0.4
	B	16.5 ± 2.5 12 ± 1.3	−3.4 ± 0.3	15.5 ± 3.1 12.8 ± 1.5	−1.7 ± 0.4
LG-1853	A	11 ± 3.9 12.4 ± 2.7	−4.3 ± 1.3	4.5 ± 1.2 17.6 ± 2.2	−9 ± 0.5
	B	20.1 ± 1.2 13.8 ± 2.4	−0.8 ± 0.3	17.1 ± 1.2 14.6 ± 2.9	−0.3 ± 0.1
LG-1829	A	11.4 ± 2.1 17.4 ± 2.6	−3.0 ± 0.5	7.9 ± 1.7 15.8 ± 2.9	−6.5 ± 1
	B	18.5 ± 2.5 11 ± 2	−2.3 ± 0.3	13.5 ± 2.8 11.4 ± 1.1	−5.3 ± 0.8

The interaction energies are defined as the sum of short-range Lennard–Jones and Coulombic interaction energies (mean ± SD)

At the 100 ns snapshot of the MD simulation, two distinct binding orientations of **LG-1781** were observed in the active site of GB-inhibited *h*AChE. In the first orientation (**GB–LG-1781-A**; Fig. 5, panel A), the quaternary nitrogen of the pyridinium moiety is positioned proximal to the GB, with the distance between the oximate oxygen of LG-1781 and the GB phosphorus atom measuring 4.4 Å. This arrangement is stabilized by a hydrogen bond between the hydroxyl group of the non-quaternary pyridine ring and Gly79 and another between the oximate group and Thr75. Dipole–dipole interaction is observed between the non-quaternary moiety and Asn87. In contrast, the second GB-related orientation (**GB–LG-1781-B**; Fig. 5, panel B) places the non-quaternary moiety closer to GB, but increases the oximate–phosphorus distance to 6.4 Å. Here, a hydrogen bond forms between the oximate group of the quaternary ring and Gly366, while the protonated nitrogen is positioned in proximity to Gly342, without formation of a persistent stabilizing interaction. Dipole–dipole interaction is also observed between the uncharged nitrogen of the pyridine ring and Asp74.

Similarly, two distinct orientations were identified for VX-inhibited *h*AChE. In the first configuration (**VX–LG-1781-A**, 60 ns; Fig. 6, panel A), the quaternary nitrogen is located near the VX phosphorus, but the oximate group is relatively distant, at 7.7 Å. This orientation is primarily stabilized by cation–π interactions between the quaternary ammonium moiety and the aromatic residues of the active-site gorge (notably Trp86), further supported by a hydrogen bond between the hydroxyl group of Tyr124 and the non-quaternary nitrogen. A potential π–π interaction may also occur between the non-quaternary ring and Tyr341, and dipole–dipole interaction is observed between the oximate group of the non-quaternary ring and Ser203. In the second configuration (**VX–LG-1781-B**, 100 ns; Fig. 6, panel B), the non-quaternary nitrogen is positioned closer to VX, with the oximate group at a shorter distance of 5.1 Å from the phosphorus atom. This conformation features a cation–π interaction between Tyr341 and the protonated nitrogen, which is further stabilized by local hydrogen-bonding interactions within the active-site gorge. Additionally, a weak hydrogen bond is formed between the distal oximate group and Asn87, and a transient hydrogen-bonding contact is observed between the hydroxyl group of the non-quaternary moiety and the VX phosphorus atom.

In summary, the most favorable orientation for GB is **LG-1781**-**A**, owing to the shorter oximate–phosphorus distance and hydrogen-bonding network. In contrast, orientation **LG-1781-B** is preferred for VX, combining a favorable distance (5.1 Å) and direct interaction to the VX phosphorus. While orientation B in both systems offers extended polar interaction networks, orientation A presents a more reactive geometry for potential nucleophilic attack in the GB system. These findings are consistent with the interaction binding energies between the oxime and VX.

**Fig. 7:**
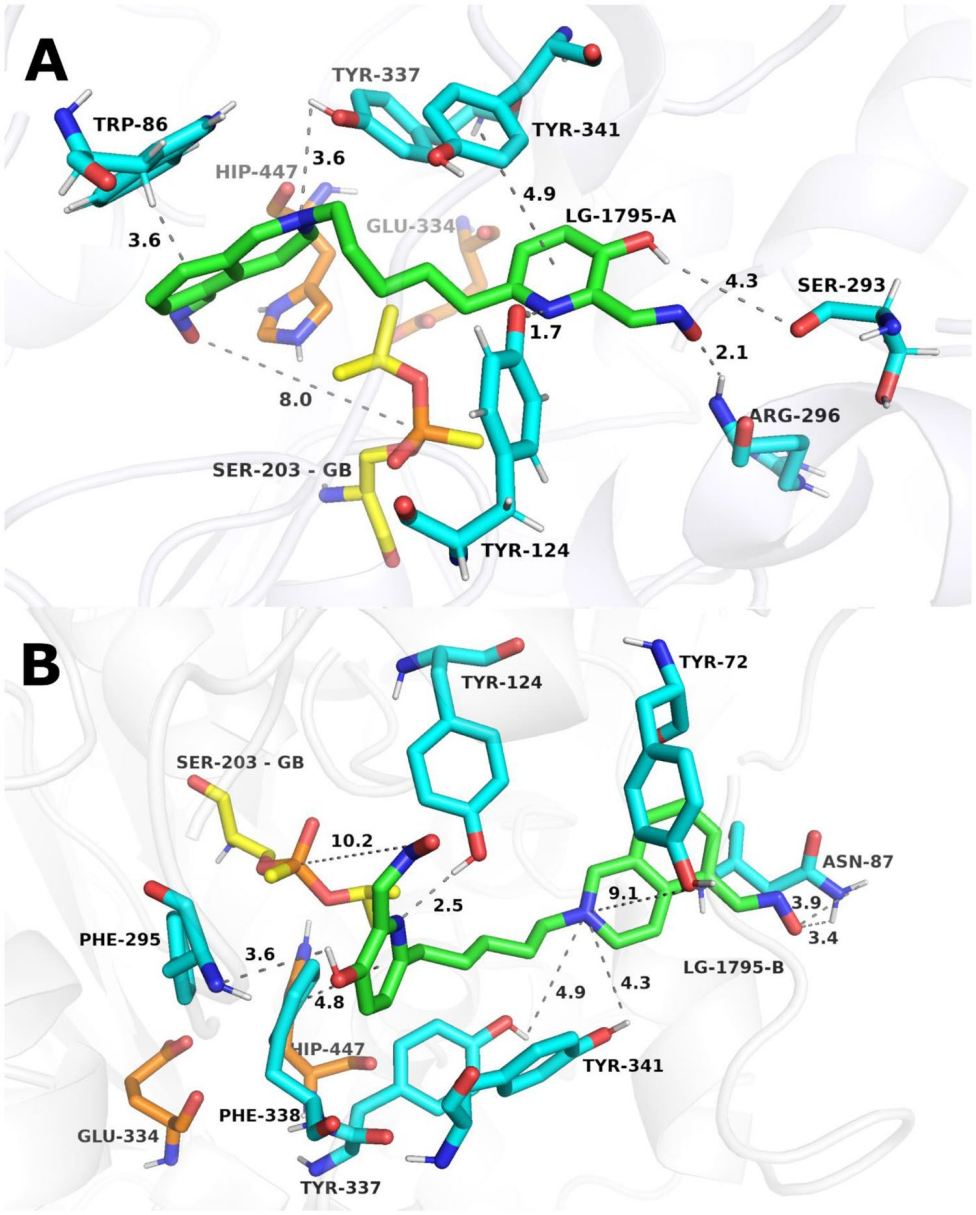
Representative top-scoring MD snapshots illustrating the binding of oxime reactivator **LG-1795** to GB-inhibited *h*AChE. **LG-1795** is shown with green carbon atoms. In panel A, the quaternary moiety of **LG-1795** is positioned proximally to the phosphorus atom of the GB adduct. In panel B, the non-quaternary moiety is oriented closer to the GB-modified site. Catalytic triad residues His447 and Glu334 are shown in orange, and the active-site serine (Ser203), covalently modified by GB, is highlighted in yellow. Amino acid residues interacting with the ligand are depicted in cyan. Distances indicated by grey dashed lines are expressed in angstroms (Å)

At the 100 ns MD snapshot, **LG-1795** adopts two distinct binding modes in the GB-inhibited *h*AChE active site. In the first orientation (**GB–LG-1795-A**; Fig. 7, panel A), the quaternary nitrogen moiety is positioned near the GB, with the distance between oximate oxygen of **LG-1795** and the GB phosphorus 8.0 Å. The pose is stabilized by a hydrogen bond between the non-quaternary pyridine ring and Tyr124 hydroxy group, and another between the oximate group of the non-quaternary pyridine ring and Arg296. A strong π–π stacking interaction with Trp86 is observed, while a potential π–π interaction with Tyr341 further contributes to stabilization. Weak electrostatic interactions involve the protonated nitrogen of the quaternary ring and Tyr337, as well as the hydroxyl group of the non-quaternary moiety with Ser293. In the second orientation (**GB–LG-1795-B**; Fig. 7, panel B), the non-quaternary moiety shifts closer to GB, increasing the oximate oxygen–GB phosphorus distance to 10.2 Å. Here, potential π–π stacking of the non-quaternary ring with Phe337 and a potential hydrogen bond or weak electrostatic interaction between the nitrogen of the non-quaternary ring and Tyr124 are observed. The protonated nitrogen of the quaternary ring forms weak electrostatic interactions with Tyr337, Tyr341 and Tyr72. The oximate oxygen of the quaternary moiety engages in a weak electrostatic interaction with Asn87.

**Fig. 8:**
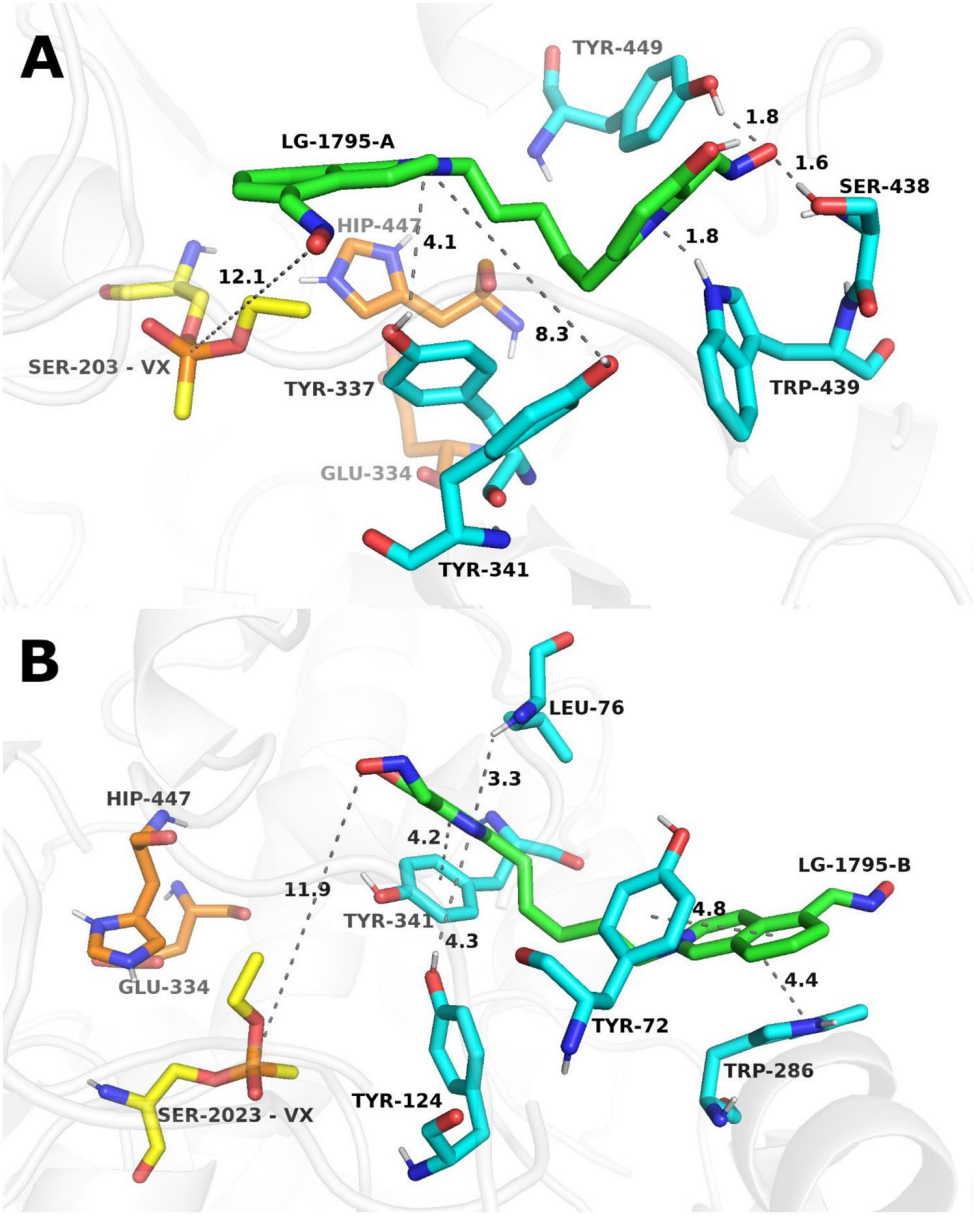
Representative top-scoring MD snapshots illustrating the binding of oxime reactivator **LG-1795** to VX-inhibited *h*AChE. **LG-1795** is shown with green carbon atoms. In panel A, the quaternary moiety of **LG-1795** is positioned proximally to the phosphorus atom of the VX adduct. In panel B, the non-quaternary moiety is oriented closer to the VX-modified site. Catalytic triad residues His447 and Glu334 are shown in orange, and the active-site serine (Ser203), covalently modified by VX, is highlighted in yellow. Amino acid residues interacting with the ligand are depicted in cyan. Distances indicated by grey dashed lines are expressed in angstroms (Å)

At the 60 ns snapshot of the MD simulation (**VX-LG-1795-A**; Fig. 8, panel A), the quaternary moiety of **LG-1795** is positioned closer to VX, with the oximate oxygen–VX phosphorus distance measuring 12.1 Å. In this orientation, a hydrogen bond is formed between the nitrogen of the non-quaternary ring and Trp439, while the oximate oxygen of the non-quaternary moiety establishes strong hydrogen bonds with Tyr449 and Ser438, providing significant stabilization. Weak electrostatic interactions are observed between the protonated nitrogen of the quaternary ring and the hydroxyl groups of Tyr337 and Tyr341. In contrast, at 80 ns (**VX-LG-1795-B**; Fig. 8, panel B), the non-quaternary moiety is located closer to VX, with the oximate oxygen–VX phosphorus distance slightly reduced to 11.9 Å. This arrangement allows for possible π–π interactions between the quaternary ring and Tyr72 as well as Trp286, and between the non-quaternary ring and Tyr341. Additionally, weak electrostatic interactions occur between the nitrogen of the non-quaternary ring and Tyr124 and Leu76.

MD simulations revealed that **LG-1795** adopts two alternative binding orientations in both GB- and VX-inhibited *h*AChE. In the GB complex, stabilization was mainly achieved through π–π interactions (Trp86, Tyr341, Phe337) and hydrogen bonds (Tyr124, Arg296), with the quaternary-oriented pose positioning the oximate group closer to the phosphorus atom (8.0 Å) compared to the non-quaternary orientation (10.2 Å). In the VX complex, strong hydrogen bonds with Trp439, Tyr449, and Ser438 or π–π stacking with Tyr72, Trp286, and Tyr341 dominated the stabilization. Collectively, these results indicate that **LG-1795** binding is governed by a balance of π–π and hydrogen bonding interactions, while the ligand orientation critically influences the proximity of the oximate group to the reactive phosphorus center.

To further assess the predictive value of computational descriptors, we performed a correlation analysis comparing in silico parameters with experimentally determined kinetic and affinity constants. Both Pearson’s correlation coefficients (assessing linear relationships) and Spearman’s correlation coefficients (capturing monotonic trends) were calculated and visualized in a correlation heatmap with corresponding significance levels (Table 7). A strong and statistically significant positive linear correlation was observed between the kinetic rate constants *k*_*r*_ and *k*_*r2*_ (Pearson *r* = 0.708, *p* < 0.001), alongside a moderate monotonic association (Spearman ρ = 0.543, *p* < 0.001), indicating robust agreement between these experimentally derived parameters. Among the in silico descriptors, the interatomic distance metric between hydroxyl group of Ser203 and the phosphorus of GB or VX (OG-P) showed a moderate positive correlation with *k*_*r*_ (Pearson *r* = 0.574, *p* < 0.001) and a comparable monotonic correlation with *k*_*r2*_ > (Spearman ρ = 0.535, *p* < 0.05), suggesting its relevance as a structural predictor of kinetic behavior. Furthermore, the predicted protein–ligand interaction energy (PROT–LIG) demonstrated a moderate negative correlation with the dissociation constant *K*_*D*_ > (Pearson *r* = –0.516, *p* < 0.05; Spearman ρ = –0.494, *p* < 0.05), consistent with the expected relationship between stronger binding and lower affinity values. Collectively, these results highlight specific in silico metrics that capture both linear and non-linear relationships with key experimental parameters, reinforcing their applicability in early-phase screening and computational model development.

**Table 7:**
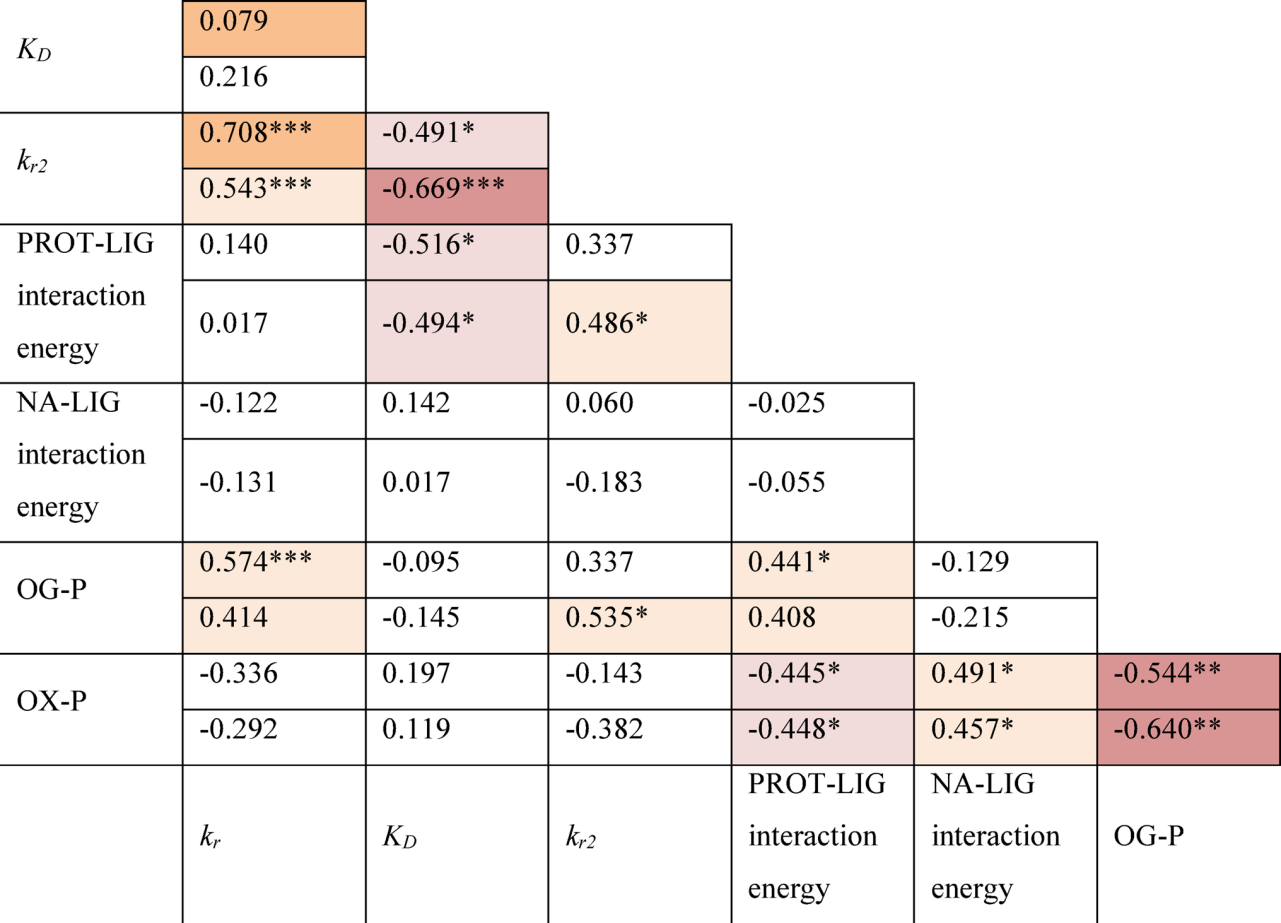
Heatmap of correlation coefficients between in silico descriptors and in vitro parameters

The upper cell of the matrix displays Pearson correlation coefficients, while the bottom cell shows Spearman’s correlation coefficients. Parameters include reaction rate constant (*k*_*r*_), dissociation constant (*K*_*D*_), second-order reactivation rate constant (*k*_*r2*_), interaction energy between the active-site of GB or VX-inhibited enzyme and the oxime (PROT-LIG interaction energy), interaction energy between the NA and ligand (NA-LIG interaction energy), average distance between hydroxyl group of Ser203 and the phosphorus of GB or VX (OG-P), and shortest distance between oximate oxygen and the phosphorus of GB or VX (OX-P). Asterisks denote statistical significance levels: * < 0.05, ** < 0.01, *** < 0.001. Positive correlations are depicted in shades of orange, while negative correlations are shown in shades of red. The strength of the correlation is indicated by color intensity: darker hues represent strong correlations, while lighter hues denote more moderate correlations

### Decomposition of OP surrogates by novel reactivators

The concept of employing oxime-based reactivators for the direct chemical degradation of OPs was initially explored in earlier studies, including the use of a phenyl-VX analogue to screen pyridine- and benzene-based nucleophiles (Saint-André et al. 2011b), as well as a high-throughput screening (HTS) campaign utilizing a fluorogenic OP surrogate (Amitai et al. 2021). Building on these foundational approaches, our recent work further demonstrated that certain reactivators can mediate OP degradation alongside enzymatic reactivation (Gorecki et al. 2024). This strategy was also applied in the current study. Four newly synthesized reactivators – **LG-1154**, **LG-1781**, **LG-1786**, and **LG-1795**—were evaluated for their ability to directly degrade the GB surrogate (NIMP) and VX surrogate (NEMP), as summarized in Table 8. These surrogates were selected due to their improved detectability via UV–Vis spectroscopy. While nucleophilicity is traditionally considered the primary determinant of degradation potential, our results suggest that it is not the sole contributing factor. To quantify nucleophilic strength, the p*K*_a_ values of the oxime functional groups were determined and compared with those of established clinical standards. The reference oximes exhibited p*K*_a_ values below 8, indicating a higher proportion of the deprotonated oximate species under physiological conditions. In contrast, most of the novel compounds displayed p*K*_a_ values above this threshold, which would theoretically correspond to a reduced capacity for nucleophilic attack on the electrophilic phosphorus center. As expected, the oxime of **LG-1786** exhibits a higher p*K*_a_ value compared to **LG-1781** and **LG-1795**, which is consistent with the absence of conjugative stabilization of its oximate form.

**Table 8:** Experimentally determined p*K*_a_ values for novel reactivators and clinical standards and their ability to directly decompose GB and VX surrogates in a ratio of 1.25:1 (compound to surrogate)

Compound	MarvinSketch 24.3.2 calculated acidic p*K*_a_	UV–vis determined p*K*_a_^1,2^	GB-surrogate decomposition T_1/2_ [min] ^1,3^	VX-surrogate decomposition T_1/2_ [min] ^1,3^
2-PAM ^4^	7.63	7.9 ± 0.01	6.1	8.5
LüH-6 ^4^	oxime 1: 7.51 oxime 2: 8.11	7.96 ± 0.02	6.7	19.7
HI-6 ^4^	oxime: 5.88 amide: 12.31	oxime: 7.43 ± 0.02 amide: 11.46 ± 0.26	13.5	25.9
LG-1154	oxime 1: 10.33 phenol: 7.81 oxime 2: 9.43	9.69 ± 0.27 7.97 ± 0.25	4.2	2.9
LG-1781	oxime 1: 10.26 phenol: 7.79 oxime 2: 9.01	8.44 ± 0.05 8.36 ± 0.03	> 60	14.3
LG-1786	oxime 1: 10.24 phenol: 8.09 oxime 2: 7.48	9.39 ± 0.05 8.64 ± 0.04	> 60	14.9
LG-1795	oxime 1: 10.24 phenol: 7.87 oxime 2: 6.83	8.70 ± 0.09 8.13 ± 0.03	> 60	6.0

^1^The results are expressed as the mean values from at least three independent experiments. ^2^Measurements were taken in phosphate-pyrophosphate buffers (pH from 5 to 13), with UV spectra for each pH point across the 200–650 nm wavelength range, as previously described (Radić et al. 2012). ^3^Measured in ratio 1:1.25 (surrogate to reactivator) at a concentration of 1 mM at physiological pH. ^4^Data taken from (Gorecki et al. 2024)

However, experimental results contradicted this assumption: **LG-1154**, despite its higher p*K*_a_, demonstrated the most pronounced degradation of OP surrogates. This apparent discrepancy highlights that oxime nucleophilicity alone does not dictate degradation efficiency. Instead, reactivity is likely modulated by additional structural and electronic factors. Within this framework, the imidazolium-2-aldoxime scaffold appears to function as a privileged motif, combining favorable orientation, charge distribution, and electronic properties conducive to surrogate decomposition.

### Degradation of GB, VX and A234 by selected reactivators

**Fig. 9:**
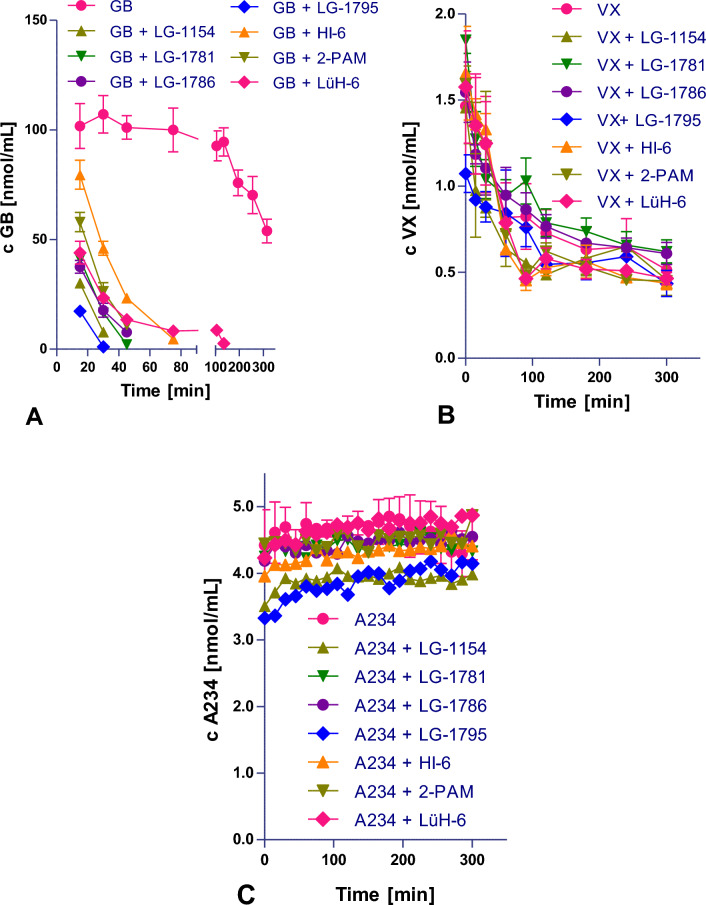
Time curve degradation of **A** GB in PBS by selected reactivators in 1:10 (GB to reactivator, molar concentration) ratio with or without reactivator; **B** VX in PBS by selected reactivators in 1:10 (VX to reactivator, molar concentration) ratio with or without reactivator; and **C** A234 in PBS by selected reactivators in 1:10 (A234 to reactivator, molar concentration) ratio with or without reactivator. The results are expressed as mean ± SD (n = 3)

To assess the true potential of the selected reactivators for dual biological activity—enzymatic reactivation and direct chemical degradation—authentic NAs (GB, VX, and A234) were employed (Fig. 9A-C and Table 9). Prior to the experiment, all novel oximes were confirmed to be stable under the specified LC conditions (Figure S24). Degradation was monitored by LC–MS as a decrease in the concentration of unbound agent in the solution. The reactivator-to-agent ratio was set to 10:1; when notable OP decomposition was observed, additional assays were conducted at 5:1 and 1:1 ratio. Whereas the VX surrogate underwent rapid decomposition in prior experiments (Table 8), only minimal degradation of authentic VX was observed (Fig. 9B), even at a reactivator-to-agent ratio of 10:1. A similar lack of degradation was observed for A234 (Fig. 9C), which exhibited greater stability in solution than either GB or VX. GB showed appreciable susceptibility to degradation (Fig. 9A), with this effect detectable even at equimolar (1:1) concentrations (see Figure S23A-C for all GB degradation curves). When degradation kinetics could be adequately described by a first-order model, apparent half-lives were calculated, apart from **LG-1154** and **LG-1795** at a 10:1 oxime-to-agent ratio, where the degradation was too rapid, not allowing analysis of enough time points to obtain the kinetic profiles (Table 9). Among the tested compounds, **LG-1154** and **LG-1795** exhibited the highest degradative activity (see Fig. 9A). Overall, these results indicate that imidazolium-2-aldoxime and pyridinium-4-aldoxime scaffolds are associated with enhanced organophosphate degradation under the applied experimental conditions. It should be emphasized that, in contrast to the sarin surrogates, GB undergoes spontaneous hydrolysis in an aqueous environment even in the absence of an oxime. Under the applied experimental conditions (GB concentration 80 nmol/mL in PBS, pH 7.4), the decrease in GB concentration followed an approximately linear time course, consistent with apparent zero-order kinetics; therefore, a first-order kinetic model is not applicable in this case. Nevertheless, it can be reasonably assumed that the presence of an oxime further accelerates GB degradation. Consequently, the apparent half-lives reported in Table 9 reflect the combined contribution of spontaneous hydrolysis of GB in water and oxime-mediated degradation. Accordingly, apparent half-life values are reported only for experimental conditions consistent with pseudo-first-order kinetics.

**Table 9:** Experimentally determined ability of novel reactivators and clinical standards to promote direct GB degradation at compound-to-agent ratio 10:1, expressed as apparent half-lives (T_1/2_)

Compound	GB decomposition T_1/2_ [min] ratio 10:1
2-PAM	14.2
LüH-6	13.8
HI-6	20.3
LG-1154	NA ^1^
LG-1781	19.4
LG-1786	15.1
LG-1795	NA ^1^

^1^NA, not applicable—apparent half-life could not be determined because the degradation kinetics did not conform to a first-order model

Half-life values were calculated using one-phase exponential decay non-linear regression implemented in GraphPad Prism 9 (GraphPad Software, San Diego, CA, USA)

Despite these findings, the physiological relevance of such in-solution degradation remains questionable. Although our assay used PBS at physiological pH 7.4, it lacks critical compartments such as plasma proteins or tissue distribution, which affect bioavailability. Comparable in vitro degradation studies have been reported for nerve agents including VX, sarin, cyclosarin, and soman using oxime–hydroxamate hybrids (Amitai et al. 2016), and for VX using sulfonatocalixarenes (Schneider et al. 2016), typically requiring high reactivator-to-agent ratios (e.g., 50:1) (Schneider et al. 2016). Amitai and colleagues further demonstrated protective effects in vivo, but primarily under pretreatment conditions (Amitai et al. 2016), underscoring that OP degradation can contribute to prophylaxis yet remains less feasible as a therapeutic approach once intoxication has occurred. The different physicochemical properties of individual OPs also significantly influence their physiological fate. GB—due to its small and lipophilic structure—rapidly penetrates the central nervous system (CNS) and exhibits a high affinity for plasma proteins, which markedly reduces the concentration of unbound agents in circulation. VX shares similar pharmacokinetic properties, albeit with slower CNS entry (Bajgar 2004; Costanzi et al. 2018). As such, while the observed decomposition of GB is experimentally valid, its in vivo relevance may be limited in post-exposure therapeutic settings. Conversely, the case of A234 may carry greater physiological implications. Recent in vivo studies have confirmed slower pharmacokinetics of A234 (Hrabinova et al. 2024). Nevertheless, under the current experimental conditions, no measurable degradation of A234 was observed, indicating that the tested reactivators have limited capacity to chemically decompose this agent under physiologically relevant conditions.

### In vivo toxicity study

Prior to initiating in vivo proof-of-concept studies, the acute toxicity and solubility of the novel oxime reactivators in formulation were systematically evaluated in mice. All compounds were dissolved in phosphate-buffered saline (PBS) supplemented with 10% Kolliphor® (v/v) to assess their maximum achievable solubility and chemical stability. Under these conditions, the following solubility limits were determined: **LG-1154**—20 mg/mL, **LG-1781**—10 mg/mL, **LG-1786**—20 mg/mL, and **LG-1795**—10 mg/mL.

Subsequently, the maximum tolerated dose (MTD) was determined for each compound based on clinical observations, macroscopic necropsy findings, and histopathological analysis (Table S7). At the maximal soluble dose of 100 mg/kg, **LG-1781** and **LG-1795** were well tolerated, although both induced mild to moderate tremors (+ + / +) and reduced spontaneous activity ( +). In females treated with **LG-1781**, additional respiratory abnormalities (+ +) were observed at this dose. Similar signs were recorded in animals treated with **LG-1786**, including respiratory changes (+ +), tremors ( +), and reduced activity ( +), all of which resolved within one hour. Due to its higher acute toxicity, the MTD for **LG-1786** was defined as 50 mg/kg in both sexes. More pronounced toxic effects were observed for **LG-1154**, particularly in female animals. While males tolerated the 100 mg/kg dose with only moderate signs of toxicity, all females succumbed at this dose, prompting an assignment of a MTD of 50 mg/kg for females. Due to its unfavorable safety profile and lower reactivation capacity, **LG-1154** was excluded from further in vivo evaluation (see Table S7 for detailed symptomatology and toxicity data). For comparison, the MTDs of the established oxime standards **LüH-6** and **HI-6** were previously determined to be approximately 150 mg/kg (Hepnarova et al. 2019). Compared to earlier studies, this toxicity evaluation followed a more rigorous protocol with expanded behavioral and clinical monitoring, which may account for slight differences in reported tolerability.

Macroscopic examination of treated animals revealed no gross pathological changes in skeletal muscle (site of injection), kidneys, or liver. These findings were further corroborated by histopathological analysis (Table S8), which showed no treatment-related alterations in renal or hepatic tissue following administration of **LG-1154**, **LG-1781**, **LG-1786**, or **LG-1795**.

### Pharmacokinetics study

**Fig. 10:**
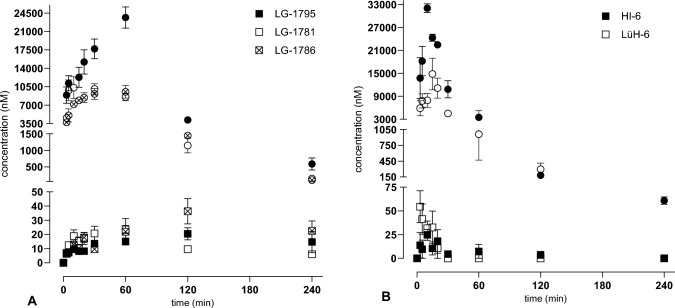
**A** Plasma (○) and brain (□) profiles following a single intramuscular administration (**LG-1781** at 25 mg/kg; **LG-1786** at 25 mg/kg; **LG-1795** at 28.8 mg/kg; equimolar doses). **B** Plasma (○) and brain (□) profiles following a single intramuscular administration (**HI-6** and **LüH-6** both at 21.9 mg/kg; equimolar doses to LG compounds). The male mice (Balb/c) were used in pharmacokinetic studies. The results are expressed as mean ± SEM (n = 4)

Pharmacokinetic profiles of the selected oxime reactivators—**LG-1781**, **LG-1786**, **LG-1795**—and the reference standards **LüH-6** and **HI-6** were evaluated following single intramuscular (*i.m*.) administration at equimolar doses (0.1 mL/10 g; 25 mg/kg for **LG-1781** and **LG-1786**, 28.8 mg/kg for **LG-1795**, 21.9 mg/kg for **HI-6** and **LüH-6**). All animals tolerated the treatment well and exhibited no adverse clinical signs throughout the experiment. Plasma and brain concentrations were measured at predefined time points, and all compounds were successfully detected in both compartments (Figs. 10A and B; Table 10).

**Table 10:** Pharmacokinetic parameters in plasma and brain following a single intramuscular administration (**LG-1781** at 25 mg/kg; **LG-1786** at 25 mg/kg; **LG-1795** at 28.8 mg/kg; **HI-6** at 21.9 mg/kg; **LüH-6** at 21.9 mg/kg; all in equimolar doses) in male mice (Balb/c)

PLASMA	LG-1781	LG-1786	LG-1795	HI-6	LüH-6
C_max_ (nM)	11 344 ± 1 580	9 962 ± 916	23 945 ± 1 823	32 048 ± 1 147	16 888 ± 3 300
T_max_ (min)	13.75 ± 2.39	45.00 ± 8.66	47.50 ± 12.50	10.00 ± 0.00	10.75 ± 4.05
*k*_a_ (min^−1^)	0.104 ± 0.091	0.145 ± 0.037	0.090 ± 0.026	0.176 ± 0.153	0.363 ± 0.217
t_1/2abs_ (min)	6.68 ± 6.06	4.79 ± 0.99	7.67 ± 3.02	3.93 ± 3.23	1.91 ± 1.23
AUC_total_ (nM × min)	813 283 ± 84 102	793 128 ± 81 106	1 938 760 ± 198 512	875 752 ± 39 751	360 512 ± 18 004
λ_z_ (min^−1^)	0.022 ± 0.001	0.025 ± 0.001	0.021 ± 0.001	0.021 ± 0.001	0.055 ± 0.025
Half-life (min)	32.19 ± 0.62	27.78 ± 0.85	34.10 ± 2.02	23.87 ± 1.04	18.83 ± 4.95
MRT (min)	55.55 ± 1.72	59.72 ± 1.33	68.75 ± 2.27	28.43 ± 0.81	29.40 ± 5.55
CL/F (L/min/kg)	0.10 ± 0.01	0.10 ± 0.01	0.04 ± 0.00	0.07 ± 0.01	0.17 ± 0.01
V_z_/F (L/kg)	4.50 ± 0.57	3.94 ± 0.37	1.95 ± 0.10	3.42 ± 0.14	4.45 ± 1.09
*BRAIN*					
C_max_ (nM)	25.74 ± 4.18	58.19 ± 22.81	21.28 ± 3.70	43.97 ± 6.29	65.29 ± 16.59
T_max_ (min)	40.00 ± 12.25	120.00 ± 0.00	105.00 ± 15.00	11.00 ± 3.00	4.00 ± 1.00
AUC_total_ / AUC_*i*_ (nM × min)	3 791 ± 492 (AUC_total_)	7 701 ± 2 704 (AUC_*i*_)	3 425 ± 975 (AUC_*i*_)	792 ± 516 (AUC_total_)	574 ± 184 (AUC_total_)
Kp_brain_ (AUCbrain/AUCplasma)	0.466	0.971	0.177	0.091	0.159

C_max_, maximal plasma or brain concentration; T_max_, time to C_max_; *k*_a_, absorption rate constant; t_1/2abs_, absorption half-life; AUC_total_/AUC_*i*_, area under curve/bioavailability; λ_z_, elimination constant in terminal phase of curve, half-life in elimination phase; MRT, mean residence time; CL, clearance; V_z_, elimination rate constant; F, fraction absorbed (bioavailability). All values are expressed as mean ± standard error (SE) of four independent measurements

Among the newly developed oximes, **LG-1795** achieved the highest plasma exposure, as indicted by its C_max_ and AUC_total_ values. In comparison, **HI-6** demonstrated superior systemic absorption overall, reaching nearly twice the plasma concentration of **LüH-6**, when administered at the same equimolar dose. Notably, **HI-6** also displayed the shortest T_max_, indicating rapid systemic uptake. In contrast, the new oximes displayed markedly slower elimination kinetics and prolonged systemic residence times, as indicated by their lower clearance (CL/F) and higher mean residence time (MRT) values. This extended plasma exposure may be advantageous for maintaining therapeutic concentration over prolonged periods, thereby supporting the feasibility of less frequent dosing or continuous infusion strategies. This approach is well established in clinical pharmacotherapy, where agents with slower clearance and longer half-lives achieve more stable target concentrations under continuous infusion compared to intermittent dosing (Kuti et al. 2004; Van Herendael et al. 2012).

Despite promising systemic parameters, most tested compounds—including the standards—exhibited low penetration into the CNS, with brain concentrations not exceeding 1% of their respective plasma levels. **LG-1786** was an exception, achieving a brain-to-plasma ratio of 0.971—markedly higher than that of all other novel reactivators and clinical standards. Moreover, **LG-1786** maintained stable brain concentrations for up to 240 min post-administration. In contrast, **HI-6** and **LüH-6** demonstrated rapid brain entry followed by swift elimination. This kinetic profile of **LG-1786** may support sustained CNS availability, a critical factor for reactivating centrally inhibited AChE in NA intoxications.

### In vivo efficacy assessment: ChE reactivation and clinical symptomatology

**Fig. 11:**
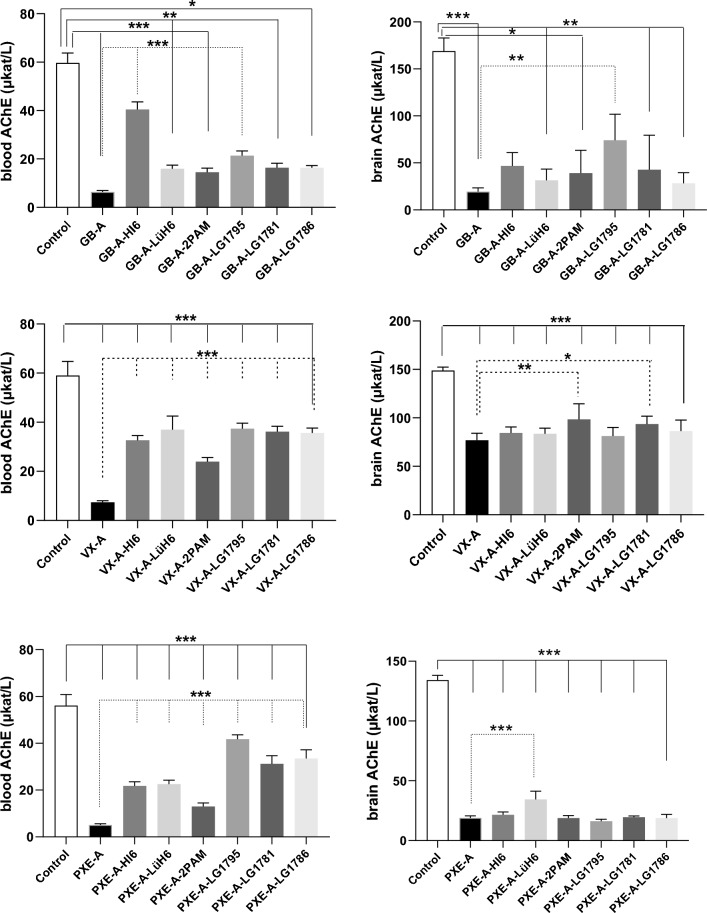
AChE reactivation with tested oximes (*i.m.*) in mice intoxicated with GB, VX, and PXE (1 × LD_50_*, i.m.*) in blood and brain. The results are expressed as mean ± SEM (n = 7). Dosing corresponds to Table 10 (protective ratio)

To comprehensively assess the therapeutic potential of **LG-1781** and **LG-1786** (both at 25 mg/kg), and **LG-1795** (28.8 mg/kg), their ability to reactivate OP-inhibited ChE in vivo was evaluated in a murine model following intoxication with GB, VX, or PXE at 1 × LD_50_ (*i.m*.) (Fig. 10 for *m*AChE, Fig. 11 for *m*BChE). All animals received co-administration of atropine (10 mg/kg) as standard symptomatic treatment (Kassa and Karasova 2021). For comparative purposes, three reference oximes were included: **HI-6** (21.9 mg/kg), a broad-spectrum reactivator of NA-inhibited AChE (Maxwell et al. 2008); **LüH-6** (21.9 mg/kg), considered the standard of care for pesticide poisoning (Lorke and Petroianu 2019); and **2-PAM** (10.4 mg/kg), selected for its relatively superior CNS penetration (Knittelova et al. 2024). All oximes were administered at equimolar, non-toxic doses, ensuring comparability and eliminating confounding effects related to toxicity. This approach contrasts with previous studies where oximes were typically compared at equitoxic doses (Kassa et al. 2012, 2025). In parallel, a Functional Observational Battery (FOB) was employed to evaluate neurobehavioral and physiological responses following intoxication and treatment. This validated behavioral screening method enables the systematic quantification of functional impairments and recovery patterns across three principal domains: (i) activity and neuromuscular coordination (Tables S10 for GB, S13 for VX, and S16 for PXE); (ii) sensorimotor integration and excitability (Tables S11 for GB, S14 for VX, and S17 for PXE); and (iii) autonomic nervous system function (Tables S12 for GB, S15 for VX, and S18 for PXE). An explanation of scoring and definitions used across all domains is provided in Table S9.

Following GB intoxication, only **HI-6** and **LG-1795** elicited significant reactivation of whole-blood AChE, with **LG-1795** being the only compound to induce a statistically significant reactivation in the brain (Fig. 10). In contrast, **HI-6** afforded the highest reactivation of plasma BChE, suggesting differential selectivity or accessibility between the two enzymes under these conditions (Fig. 11). Correspondingly, **LG-1795** was the only novel oxime that visibly alleviated acute cholinergic symptoms within the first hour post-exposure—including tremors and hypoactivity. FOB assessments confirmed this effect, showing that all reactivator treatments improved neuromuscular and sensorimotor parameters within 2 h; however, complete normalization of behavioral markers was observed only with **LG-1795** and the reference compound **HI-6**. Autonomic dysfunctions such as lacrimation, altered pupil responses, and respiratory distress were also mitigated more effectively in these groups. In contrast, treatment with **LG-1781**, **LG-1786**, or **LüH-6** led to only partial improvements in both enzymatic reactivation and clinical manifestations. These findings support the superior performance of **LG-1795** in reversing GB-induced neurotoxicity under the tested conditions.

**Fig. 12:**
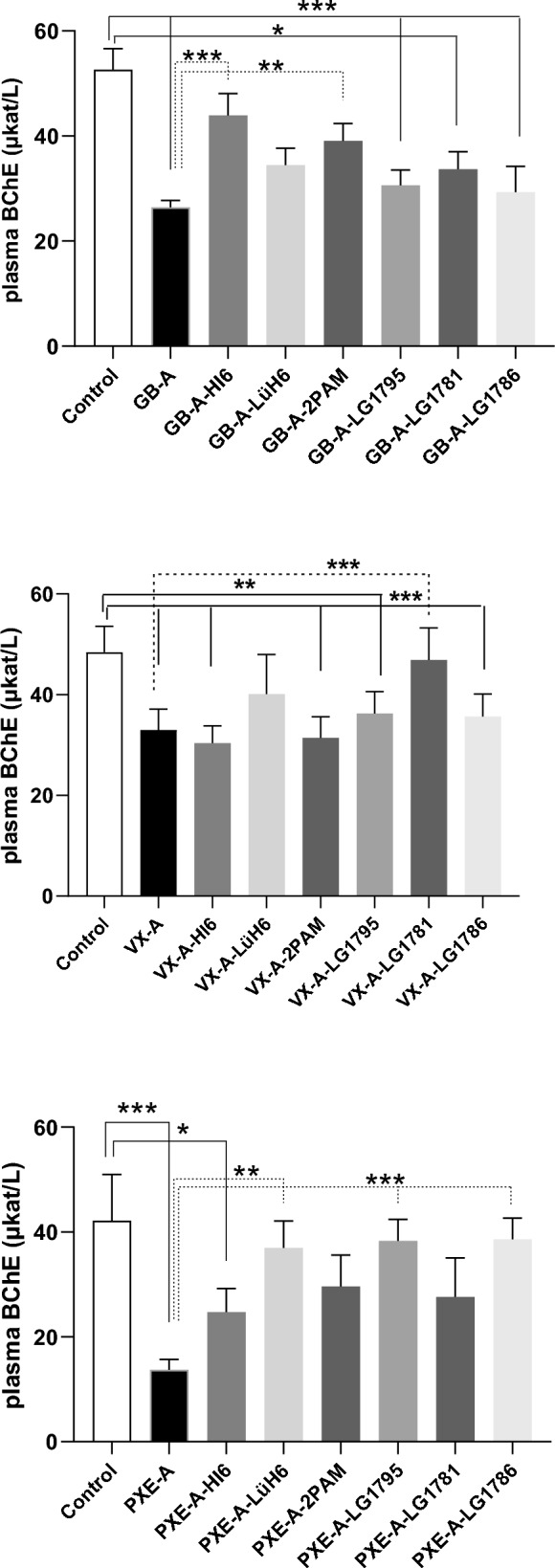
BChE reactivation with tested oximes (*i.m.*) in mice intoxicated with GB, VX, and PXE (1 × LD_50_, *i.m.*) in plasma. The results are expressed as mean ± SEM (n = 7). Dosing corresponds to Table 10 (protective ratio)

Following VX intoxication, all tested oximes demonstrated notable reactivation capacity in whole blood, with **LüH-6** emerging as the most effective among the reference compounds (Fig. 11). The newly developed oximes **LG-1781**, **LG-1786**, and **LG-1795** showed comparable or superior efficacy to **2-PAM**. In brain tissue, **LG-1781** and **2-PAM** elicited statistically significant reactivation effects, although intergroup differences remained moderate. When plasma BChE was evaluated, **LG-1781** exhibited the highest reactivation potential, outperforming both standard oximes and other novel candidates (Fig. 12). This finding highlights **LG-1781**’s robust capacity to restore peripheral cholinesterase activity following VX exposure. Regarding functional outcomes, VX exposure induced less severe neuromuscular and autonomic disruptions compared to GB or PXE. Nevertheless, the tested oximes contributed to full normalization of affected neuromuscular markers (e.g., activity, tremor, mobility) within 24 h. **LG-1781** achieved favorable outcomes, including the restoration of reflexes and respiration. Sensorimotor responses were largely normalized across all treatment groups by 2 h post-treatment, while signs of autonomic dysfunction—such as lacrimation or respiratory distress—resolved more rapidly compared to those observed with other OPs. Collectively, these findings indicate that the new oximes—particularly **LG-1781**—provide efficient symptomatic recovery and AChE reactivation following VX poisoning.

All tested oximes achieved significant reactivation of AChE activity in blood following PXE exposure. Among the newly developed compounds, **LG-1795** demonstrated the highest efficacy, followed by **LG-1781** and **LG-1786** (Fig. 11). All three outperformed **2-PAM** and demonstrated comparable or superior efficacy to **LüH-6**, the most effective reference standard. In contrast, brain AChE reactivation remained low across all treatments, except for **LüH-6**, which produced a statistically significant increase. In plasma, **LG-1795** and **LG-1786** also demonstrated favorable BChE reactivation, comparable to or slightly exceeding that of **LüH-6—**further supporting their potential as dual-function reactivators (Fig. 11). Behavioral analysis revealed the most severe functional impairments following PXE intoxication, particularly in neuromuscular activity and sensorimotor response. Two hours post-intoxication, all groups receiving oxime-based treatments exhibited improvements in key markers, including spontaneous activity, tremors, clonic movements, and responsiveness to handling. The combination of atropine with novel oximes proved more effective than atropine alone, which failed to normalize several endpoints. Despite partial recovery, some deficits—including impaired coordination and hypoalgesia—persisted at 24 h post-exposure in animals treated with LG compounds. **LüH-6** and **HI-6** showed more pronounced effects in restoring sensorimotor and autonomic markers at later time points. Nonetheless, **LG-1781** and **LG-1795** demonstrated encouraging efficacy in attenuating the acute toxicological manifestations of PXE.

### The protective ratios of LG-1781 and LG-1795 against GB, VX, and PXE

To evaluate the in vivo protective efficacy of the novel oximes (Table 11), LD_50_ studies were conducted using **LG-1781** (25 mg/kg) and **LG-1795** (28.8 mg/kg) in combination with atropine (10 mg/kg) and the results were compared to standard treatments (**HI-6** and **LüH-6**, both at 21.9 mg/kg plus atropine). NAs were administered at a dose of 1 × LD_50_ (GB 229 µg/kg, VX 20 µg/kg, PXE 609 µg/kg; volume: 0.1 mL/kg; *i.m.*). Treatment consisted of atropine (10 mg/kg) alone or in combination with oximes: LG-1781 (25 mg/kg), LG-1795 (28.8 mg/kg), LüH-6 (21.9 mg/kg), and HI-6 (21.9 mg/kg), administered *i.m*. 1 min after NA exposure. A wide range of muscarinic and nicotinic symptoms was observed in all intoxicated groups following the administration of GB, VX, or PXE, regardless of the treatment applied.

**Table 11:** Overview of selected oximes to reduce the lethality in mice

Poisoning/treatment	*i.m.* LD_50_ (95% CI) (μg/kg)	Protective ratio vs. the untreated group	Protective ratio vs. the atropine group
GB	196.02 (161.59–237.78)	–	–
atropine (10 mg/kg)	268.38 (238.75–301.70)	1.40	–
LüH-6 (21.9 mg/kg) + atropine	589.62 (372.86–932.38)	3.01*	2.20^#^
HI-6 (21.9 mg/kg) + atropine	764.85 (452.71–1292.22)	3.90*	2.85^#^
LG-1781 (25.0 mg/kg) + atropine	345.09 (169.57–702.29)	1.76	1.29
LG-1795 (28.8 mg/kg) + atropine	438.03 (253.22–757.73)	2.24*	1.63
VX	16.49 (15.86–17.15)	–	–
atropine (10 mg/kg)	21.57 (16.50–28.20)	1.31	–
LüH-6 (21.9 mg/kg) + atropine	115.62 (87.98–161.59)	7.01*	5.36^#^
HI-6 (21.9 mg/kg) + atropine	200.25 (143.54–279.36)	12.14*	9.28^#^
LG-1781 (25.0 mg/kg) + atropine	96.81 (88.36–106.04)	5.87*	4.49^#^
LG-1795 (28.8 mg/kg) + atropine	67.87 (39.65–116.18)	4.11*	3.15^#^
PXE	561.76 (539.61–584.82)	–	–
atropine (10 mg/kg)	1 709.02 (778.51–1 495.53)	3.04*	–
LüH-6 (21.9 mg/kg) + atropine	12 444.02 (11 063.12–13 997.28)	22.14*	7.28^#^
HI-6 (21.9 mg/kg) + atropine	5 537.91 (3 120.81–9 827.08)	9.85*	3.24^#^
LG-1781 (25.0 mg/kg) + atropine	6 186.14 (5 085.85–7 524.46)	11.01*	3.61^#^
LG-1795 (28.8 mg/kg) + atropine	7 065.03 (6 301.65–7 920.87)	12.57*	4.13^#^

*Significantly different from the untreated group based on relative median potency (RMP)

^#^Significantly different from the atropine group based on RMP

Across all tested agents, **HI-6** consistently conferred the highest protective ratios, particularly in GB and VX poisoning. **LüH-6** also showed substantial efficacy. In comparison, **LG-1781** and **LG-1795** provided moderate levels of protection against GB and VX. While both novel oximes achieved statistically significant improvements over atropine alone, their protective performance did not reach the levels of the standard compounds. Notably, in the PXE model, **LG-1781** and **LG-1795** outperformed **HI-6** and approached the efficacy of **LüH-6**, suggesting a potentially favorable profile for treating specific OP agents.

## Discussion

This study presents a new generation of asymmetric monoquaternary bisoxime reactivators with enhanced efficacy against a broad spectrum of OPs, including classical NAs and organophosphorus pesticides. Among the evaluated compounds, **LG-1795** emerged as the lead candidate—primarily due to its broad-spectrum reactivation capability—consistently outperforming some of the established oximes (**2-PAM**, **HI-6**, and **LüH-6**) across both in vitro reactivation assays and in vivo models of OP poisoning.

From a structural standpoint, **LG-1795** incorporates two chemically distinct oxime pharmacophores—3-hydroxypyridinium-2-aldoxime and isoquinolinium-5-aldoxime—within a single molecular framework. This asymmetric dual-warhead configuration enables complementary functions: one oxime acts as the primary nucleophile while the other stabilizes binding and orientation through peripheral-site or π–cation interactions. Importantly, these roles are not fixed but can interchange, allowing **LG-1795** to maintain productive binding geometries across structurally diverse OP–ChE adducts. This mechanistic versatility is reflected in the superior bimolecular reactivation rate constants (*k*_*r2*_) observed across multiple OP–ChE complexes (Table 3). This design principle aligns with prior studies suggesting that multifunctional oximes, capable of adopting alternative binding conformations, exhibit a broad substrate range and high reactivation capacity (Gorecki et al. 2020; Čadež et al. 2025; Kolić et al. 2025). In addition to its strong activity toward *h*AChE, **LG-1795** also showed efficient reactivation of OP-inhibited *h*BChE in vitro, particularly against GB- and VX-conjugates. This dual action suggests an appealing mechanistic advantage: reactivation of AChE directly restores synaptic function, whereas reactivation of BChE sustains its systemic bioscavenger role, thereby reducing the OP load reaching AChE. Although this effect was less evident in our in vivo studies, possibly because BChE inhibition was lower under those conditions or due to interspecies structural variations between animal and human BChE that affect inhibitor binding and catalytic efficiency, the in vitro findings support that dual targeting of both cholinesterases represents a promising strategy to enhance overall protection. Nevertheless, **LG-1795** showed only marginal activity against A234-inhibited AChE, which is regarded as a specific outlier within an otherwise broad-spectrum reactivation profile. MD simulations with GB- and VX-inhibited AChE further corroborated this conformational adaptability (Table 6), revealing productive binding orientations of both oxime moieties within the active-site gorge. These snapshots (Figs. 7 and 8) are consistent with the high *k*_*r2*_ experimental values and support the mechanistic rationale for **LG-1795**’s broad reactivation profile.

The in vivo data corroborated the key in vitro observations, particularly in terms of blood AChE reactivation following acute OP intoxication. All three novel oximes demonstrated significant reactivation of peripheral AChE activity across GB, VX, and PXE models (Fig. 11). **LG-1795** consistently yielded the highest reactivation, and notably it was the only compound to achieve statistically significant reactivation in the brain following GB exposure. The neurobehavioral evaluation, assessed via the FOB, further reinforced these pharmacodynamic outcomes. Specifically, **LG-1795** effectively alleviated a broad spectrum of OP-induced symptoms, including deficits in neuromuscular coordination, sensorimotor excitability, and autonomic function. Despite this favorable pharmacodynamic profile, the protective efficacy of **LG-1795** and **LG-1781—**expressed as LD₅₀-based protective ratios—remained lower than that of **HI-6** and **LüH-6**, particularly in the GB and VX models (Table 11). The disconnection between reactivation capacity and survival-based endpoints can be plausibly explained by pharmacokinetic limitations inherent in the *i.m*. administration route. While **HI-6** reached maximal plasma concentrations within approximately 10 min post-injection, **LG-1795** exhibited a delayed *T*ₘₐₓ and lower *C*ₘₐₓ, despite achieving therapeutically relevant levels (~ 10 µM) within the first few minutes (Table 10). This delay likely reduced its efficacy during the critical early window of cholinergic crisis, when rapid reactivation of AChE is essential for preventing lethality.

These findings highlight the importance of aligning pharmacokinetic profiles with the temporal pathophysiology of OP poisoning. The relatively prolonged half-life and systemic persistence of **LG-1795** could be highly advantageous in clinical dosing strategies, such as intravenous infusion or repeated bolus administration, by sustaining effective plasma concentrations during the critical phases of intoxication. Indeed, continuous infusion regimens have been demonstrated to enhance pharmacodynamic outcomes in both experimental and clinical settings. For instance, clinical protocols often employ a loading dose followed by maintenance infusion to sustain therapeutic plasma concentrations (10–20 µM) over extended periods (Eyer et al. 2007; Eddleston et al. 2008; Thiermann et al. 2009; Eddleston 2019).

Importantly, the current in vivo findings must be interpreted within the broader context of species-dependent differences in AChE architecture and OP reactivity. Previous in vitro experiments have revealed that *h*AChE exhibits distinct kinetic properties compared to its murine counterpart (*m*AChE), including differences in binding site accessibility and reactivation susceptibility (Worek et al. 2002; Tressler et al. 2024). For instance, **LG-1795** demonstrated markedly higher reactivation potency against *h*AChE than against *m*AChE, especially against GB and VX adducts. This observation raises the possibility that the true therapeutic potential of **LG-1795** may be underestimated in murine models, as indicated by protective in vivo experiments, and could manifest more favorably in human applications. Furthermore, differences in BBB permeability between species—and under pathological conditions (e.g., OP-induced neuroinflammation)—may influence central reactivation outcomes (Nair et al. 2023; Andrew et al. 2024). While the brain reactivation profiles of the novel oximes were modest overall, **LG-1795** showed evidence of CNS penetration and therapeutic engagement—an aspect that merits deeper pharmacokinetic and biodistribution analysis in future studies.

Finally, in vivo AChE reactivation should be interpreted in a functional and physiological context rather than judged solely by the extent of complete enzyme recovery. This concept is supported by experimental studies demonstrating that relatively low levels of AChE activity within critical brainstem and neuromuscular regions may be sufficient to maintain respiratory drive and muscle function (Bajgar et al. 2007b, a, 2012). Importantly, clinical observations from severe NA poisoning further corroborate this notion. In a confirmed case of A234 poisoning, recovery of spontaneous breathing was associated with approximately 30% AChE activity in red blood cells, despite the absence of full enzyme reactivation (Steindl et al. 2021). Accordingly, although AChE activities observed in the present study generally remain below control levels, the degree of reactivation achieved is likely to be physiologically meaningful and consistent with levels associated with functional recovery and survival.

## Conclusion

This study reported the rational design of bisoxime reactivators, guided by structure–activity relationship (SAR) principles and supported through molecular modeling, resulting in compounds with substantially improved pharmacological properties. Among the tested candidates, **LG-1795** consistently emerged as the most promising molecule. In vitro, **LG-1795** displayed the highest reactivation efficacy across structurally diverse OP–ChE complexes, with a second-order reactivation rate constant (*k*_*r2*_) of 16.8 mM^−1^min^−1^ averaged across five *h*AChE–OP and two *h*BChE–OP adducts. Notably, GA-inhibited *h*AChE recovered up to 74% of baseline activity after 12 h in the presence of **LG-1795**, compared with only 6.5% for **2-PAM**. Such broad-spectrum activity, characterized by concurrent reactivation of both AChE and BChE, highlights a dual mechanism that remains largely underexplored in the field of oxime antidotes. A limiting feature is its poor potency against A234, which in general remains highly resistant to reactivation. In vivo, **LG-1795** demonstrated robust pharmacokinetic properties—including good bioavailability and a prolonged half-life—allowing sustained systemic exposure. Although its intramuscular protective efficacy did not consistently surpass clinical standards, this limitation can be attributed to pharmacokinetic factors, particularly the delayed T_max_ and modest peak concentrations observed after *i.m.* dosing. Importantly, **LG-1795** restored both AChE and BChE activity in blood and brain tissue and mitigated acute toxic signs in multiple OP challenge models, highlighting its functional relevance even under suboptimal exposure profiles. The compound’s slower onset but sustained presence suggests that it may be particularly well-suited for scenarios requiring extended protection, such as repeated low-dose exposures or delayed medical intervention. Furthermore, the ability of **LG-1795** to reactivate BChE in vitro—an effect rarely reported for oximes—points to a potential dual therapeutic role, combining direct AChE rescue with preservation of BChE’s bioscavenger function. Together, these findings suggest that while immediate efficacy may depend on optimized dosing strategies (e.g., intravenous infusion or repeated bolus), the pharmacological profile of **LG-1795** provides strong rationale for its continued preclinical development.

Taken together, these findings establish **LG-1795** as a next-generation lead candidate with broad-spectrum reactivation efficacy, favorable pharmacokinetics, and dual mechanistic action. Beyond therapeutic use, its properties also suggest potential applications in bioscavenger-based prophylaxis or decontamination approaches. Future studies should therefore prioritize: (i) comprehensive toxicokinetic and safety profiling; (ii) efficacy assessment in higher-order species with AChE architectures more closely resembling that of humans; (iii) refined biodistribution and CNS penetration studies; and (iv) development of operationally suitable formulations—including autoinjectors and infusion protocols—to evaluate full translational and field readiness.

## Supplementary Information

Below is the link to the electronic supplementary material.

Supplementary file1 (DOCX 37878 KB)

## Data Availability

All data generated or analysed during this study are included in this published article and its supplementary information files.
