# Supplementary material for: Next-generation broad-spectrum reactivators for effective countermeasure against organophosphorus poisoning
Source: Arch Toxicol. 2026 Mar 16;100(6):2467–502. doi: 10.1007/s00204-026-04341-y (PMC13221350; doi:10.1007/s00204-026-04341-y)
Supplement: Supplementary file 1 — Supplementary file1 (DOCX 37878 KB) [file 204_2026_4341_MOESM1_ESM.docx]

Next-Generation Broad-Spectrum reactivators for Effective Countermeasure against Organophosphorus Poisoning

# Jana Zdarova Karasova^1,*,#^, Martina Hrabinova^1^, Alzbeta Dlabkova^1^, Vendula Hepnarova^1^, Natalie Zivna^1,2^, Monika Schmidt^2^, Valeria Sheshko^3^, Carilyn Torruellas^4^, Jakub Opravil^1^, Lukas Prchal^2^, Natalie Vanova^1^, Jakub Fibigar^1^, Zbynek Vecera^1^, Tomas Kucera^5^, Jaroslav Chladek^6^, Gabriele Horn^7^, Franz Worek^7^, Jan Marek^2,8^, Jaroslav Pejchal^1^, Daniel Jun^1^, Ondrej Soukup^1,2^, Jan Korabecny^1,2^ Lukas Gorecki^1,2,*,#^

^1^ University of Defence, Military Faculty of Medicine, Department of Toxicology and Military Pharmacy, Trebesska 1575, 500 01 Hradec Kralove, Czech Republic

^2^ University Hospital Hradec Kralove, Biomedical Research Centre, Sokolska 581, 500 05 Hradec Kralove, Czech Republic

^3^ *University of Defence, Military Faculty of Medicine, Department of Molecular Pathology and Biology, Trebesska 1575, 500 01 Hradec Kralove, Czech Republic*

^4^ U. S. Army CCDC Chemical Biological Center, Aberdeen Proving Ground, MD 21010-5424, USA

^5^ *University of Defence, Military Faculty of Medicine, Department of Military Medical Service Organization and Management, Trebesska 1575, 500 01 Hradec Kralove, Czech Republic*

^6^ Charles University, Faculty of Medicine in Hradec Kralove, *Department of Pharmacology, Simkova 870, 500 03 Hradec Kralove, Czech Republic*

^7^ Bundeswehr Institute of Pharmacology and Toxicology, Neuherbergstrasse 11, 80937 Munich, Germany

^8^ University of Defence, Military Faculty of Medicine, Department of Epidemiology, Trebesska 1575, 500 01 Hradec Kralove, Czech Republic

# contributed equally

# * Corresponding authors’ e-mails: [lukas.gorecki@fnhk.cz](mailto:lukas.gorecki@fnhk.cz) (L.G.); [jana.zdarovakarasova@unob.cz](mailto:jana.zdarovakarasova@unob.cz) (J.Z.K);

# *In vitro* reactivation screen using various enzymes

**Table S1** *In vitro* reactivation rates of OP-inhibited *h*AChE at both concentrations. The ChE activity was measured after 10 min incubation of the compound with OP-inhibited ChE.

|  | **% recovery of OP-inhibited *h*AChE ± SD** | | | | | | | | | | | | | |
| --- | --- | --- | --- | --- | --- | --- | --- | --- | --- | --- | --- | --- | --- | --- |
|  | **GB** | | **GA** | | | **VX** | | | **PXE** | | | **PXM** | | |
|  | **100 µM** | **10 µM** | | **100 µM** | **10 µM** | | **100 µM** | **10 µM** | | **100 µM** | **10 µM** | | **100 µM** | **10 µM** |
| **LüH-6** | 21 ± 1.1 | 6.7 ± 0.8 | | 5.3 ± 0.3 | < 5 | | 26 ± 0.3 | 5.3 ± 0.1 | | 61 ± 1.2 | 19 ± 0.6 | | 36 ± 0.7 | 13 ± 0.5 |
| **2-PAM** | 40 ± 0.6 | 9.0 ± 0.3 | | < 5 | < 5 | | 28 ± 0.5 | < 5 | | 12 ± 0.3 | < 5 | | 14 ± 0.3 | < 5 |
| **HI-6** | 77 ± 1.0 | 24 ± 0.5 | | < 5 | < 5 | | 65 ± 1.2 | 21 ± 0.3 | | 11 ± 0.3 | < 5 | | 17 ± 0.4 | < 5 |
| **MMB4** | 21 ± 0.6 | 6.0 ± 0.7 | | < 5 | < 5 | | 20 ± 0.5 | < 5 | | 21 ± 0.4 | < 5 | | 10 ± 0.5 | < 5 |
| **LG-1154** | 59 ± 0.6 | 24 ± 0.8 | | < 5 | < 5 | | 25 ± 1.0 | 10 ± 0.2 | | 36 ± 0.7 | 24 ± 1.0 | | < 5 | < 5 |
| **LG-1795** | 78 ± 1.5 | 48 ± 0.8 | | 7.2 | < 5 | | 79 ± 1.3 | 32 ± 0.7 | | 83 ± 0.6 | 46 ± 0.6 | | 46 ± 0.7 | 15 ± 0.7 |
| **LG-1781** | 58 ± 1.0 | 14 ± 0.3 | | 6.4 ± 0.2 | < 5 | | 60 ± 0.9 | 13 ± 0.6 | | 62 ± 1.1 | 16 ± 0.3 | | 63 ± 0.5 | 11 ± 0.3 |
| **LG-1786** | 67 ± 2.6 | 21 ± 0.9 | | 8.5 ± 0.3 | < 5 | | 82 ± 1.4 | 32 ± 0.5 | | 74 ± 1.5 | 25 ± 0.9 | | 67 ± 0.8 | 15 ± 0.3 |
| **LG-1703** | 28 ± 1.0 | 4.4 ± 0.7 | | 5.3 ± 0.2 | < 5 | | 39 ± 0.2 | 2.2 ± 0.0 | | 45 ± 0.6 | 8.2 ± 0.3 | | 49 ± 0.3 | 7.5 ± 0.3 |
| **LG-1704** | 50 ± 0.9 | 12 ± 0.9 | | 7.3 ± 0.3 | < 5 | | 55 ± 0.8 | 11 ± 0,4 | | 71 ± 0.6 | 21 ± 0.6 | | 45 ± 0.6 | 8.5 ± 0.4 |
| **LG-1829** | 61 ± 1.0 | 17 ± 0.6 | | 9.1 ± 0.5 | < 5 | | 69 ± 1.4 | 19 ± 1.2 | | 65 ± 0.8 | 14 ± 0.3 | | 49 ± 0.6 | 12 ± 0.3 |
| **LG-1853** | 54 ± 0.7 | 13 ± 0.8 | | 7.6 ± 0.2 | < 5 | | 61 ± 0.6 | 17 ± 0.8 | | 55 ± 1.2 | 13 ± 0.4 | | 48 ± 0.9 | 10 ± 0.4 |

^1^ The results are expressed as the mean of at least three experiments

**Table S2** *In vitro* reactivation rates of OP inhibited *r*AChE at both concentrations. The *r*AChE activity was measured after 10 min incubation of the compound with OP-inhibited ChE.

|  | **% recovery of OP-inhibited *r*AChE ± SD** | | | | | | | | | | | | | |
| --- | --- | --- | --- | --- | --- | --- | --- | --- | --- | --- | --- | --- | --- | --- |
|  | **GB** | | **GA** | | | **VX** | | | **PXE** | | | **PXM** | | |
|  | **100 µM** | **10 µM** | | **100 µM** | **10 µM** | | **100 µM** | **10 µM** | | **100 µM** | **10 µM** | | **100 µM** | **10 µM** |
| **LüH-6** | 26 ± 1.3 | 8 ± 0.8 | | 7 ± 0.2 | < 5 | | 35 ± 0.7 | 9 ± 0.2 | | 93 ± 1.8 | 61 ± 0.9 | | 19 ± 0.6 | < 5 |
| **2-PAM** | 36 ± 0.9 | 8 ± 0.2 | | < 5 | < 5 | | 33 ± 0.5 | 7 ± 0.4 | | 59 ± 1.1 | 11 ± 0.5 | | < 5 | < 5 |
| **HI-6** | 70 ± 1.5 | 14 ± 0.5 | | < 5 | < 5 | | 80 ± 1.2 | 20 ± 0.8 | | 50 ± 1.1 | 8 ± 0.2 | | < 5 | < 5 |
| **MMB4** | 11 ± 0.4 | < 5 | | < 5 | < 5 | | 51 ± 0.5 | 7 ± 0.9 | | 72 ± 0.4 | 15 ± 0.2 | | < 5 | < 5 |
| **LG-1154** | 69 ± 2.5 | 23 ± 0.5 | | 7 ± 1.6 | 5.0 ± 0.2 | | 25 ± 0.7 | 16 ± 0.7 | | inhibited | 65 ± 1.1 | | 30 ± 4.4 | < 5 |
| **LG-1795** | 79 ± 2.2 | 50 ± 0.9 | | 22 ± 0.9 | 13 ± 0.1 | | 99 ± 2.1 | 52 ± 1.8 | | inhibited | 94 ± 2.1 | | 75 ± 2.7 | 13 ± 0.4 |
| **LG-1781** | 51 ± 0.5 | 11 ± 0.3 | | 13 ± 0.2 | < 5 | | 89 ± 0.6 | 35 ± 0.9 | | 98 ± 1.3 | 51 ± 1.1 | | 23 ± 0.4 | < 5 |
| **LG-1786** | 81 ± 2.1 | 32 ± 0.7 | | 15 ± 0.7 | 5.2 ± 0.7 | | 99 ± 0.7 | 52 ± 1.5 | | 98 ± 1.5 | 72 ± 1.0 | | 37 ± 1.1 | 8 ± 0.0 |
| **LG-1703** | 35 ± 0.6 | 9 ± 0.5 | | 5 ± 0.2 | < 5 | | 68 ± 0.9 | 21 ± 0.6 | | 85 ± 0.8 | 20 ± 0.4 | | 12 ± 0.3 | < 5 |
| **LG-1704** | 46 ± 1.0 | 10 ± 0.7 | | 7 ± 0.2 | < 5 | | 79 ± 1.7 | 38 ± 0.6 | | 91 ± 1.0 | 48 ± 1.0 | | 13 ± 0.5 | < 5 |
| **LG-1829** | 54 ± 0.5 | 15 ± 0.4 | | 11 ± 0.3 | < 5 | | 89 ± 1.3 | 39 ± 0.7 | | 96 ± 1.4 | 54 ± 1.8 | | 19 ± 0.4 | < 5 |
| **LG-1853** | 38 ± 0.6 | 9 ± 0.5 | | < 5 | < 5 | | 74 ± 1.5 | n.d. | | 91 ± 3.1 | 45 ± 0.9 | | 18 ± 0.4 | < 5 |

^1^ The results are expressed as the mean of at least three experiments

**Table S3** *In vitro* reactivation rates of OP inhibited *m*AChE at both concentrations. The *m*AChE activity was measured after 10 min incubation of the compound with OP-inhibited *m*AChE.

|  | **% recovery of OP-inhibited *m*AChE ± SD** | | | | | | | | | | | | | |
| --- | --- | --- | --- | --- | --- | --- | --- | --- | --- | --- | --- | --- | --- | --- |
|  | **GB** | | | **GA** | | | **VX** | | **PXE** | | | **PXM** | | |
|  | **100 µM** | **10 µM** | **100 µM** | | **10 µM** | **100 µM** | | **10 µM** | | **100 µM** | **10 µM** | | **100 µM** | **10 µM** |
| **LüH-6** | 33 ± 0.8 | 10 ± 0.3 | 7 ± 0.6 | | < 5 | 22 ± 1.7 | | < 5 | | 83 ± 1.2 | 48 ± 0.9 | | 31 ± 3.7 | < 5 |
| **2-PAM** | 34 ± 0.7 | 6 ± 0.3 | < 5 | | < 5 | 12 ± 0.4 | | < 5 | | 36 ± 1.4 | 6.0 ± 0.1 | | < 5 | < 5 |
| **HI-6** | 79 ± 1.9 | 22 ± 0.9 | < 5 | | < 5 | 45 ± 1.3 | | 8 ± 1.2 | | 28 ± 0.5 | < 5 | | < 5 | < 5 |
| **MMB4** | 39 ± 1.8 | 9 ± 0.3 | < 5 | | < 5 | 13 ± 0.8 | | < 5 | | 51 ± 3.4 | 9 ± 1.2 | | < 5 | < 5 |
| **LG-1154** | 47 ± 3.1 | 15 ± 1.1 | < 5 | | < 5 | 27 ± 2.3 | | 9 ± 0.5 | | 66 ± 2.8 | 51 ± 1.3 | | < 5 | < 5 |
| **LG-1795** | 82 ± 2.3 | 39 ± 1.0 | 27 ± 1.2 | | 10 ± 0.7 | 77 ± 2.4 | | 22 ± 0.8 | | 93 ± 2.2 | 71 ± 3.4 | | 72 ± 4.0 | 27 ± 2.0 |
| **LG-1781** | 66 ± 4.0 | 27 ± 1.7 | 16 ± 1.0 | | < 5 | 72 ± 2.2 | | 17 ± 0.4 | | 84 ± 1.3 | 55 ± 0.8 | | 42 ± 1.9 | < 5 |
| **LG-1786** | 64 ± 2.3 | 24 ± 1.8 | 11 ± 1.2 | | < 5 | 76 ± 1.6 | | 20 ± 0.6 | | 78 ± 4.0 | 53 ± 2.4 | | < 5 | < 5 |
| **LG-1703** | 39 ± 0.7 | 8 ± 0.2 | 8 ± 0.7 | | < 5 | 48 ± 1.0 | | 9 ± 0.3 | | 66 ± 2.3 | 22 ± 1.2 | | 29 ± 1.5 | 6 ± 0.1 |
| **LG-1704** | 56 ± 0.4 | 17 ± 0.1 | 11 ± 0.7 | | < 5 | 63 ± 5.7 | | 15 ± 2.3 | | 81 ± 2.7 | 33 ± 1.5 | | 36 ± 0.6 | < 5 |
| **LG-1829** | 59 ± 0.5 | 19 ± 0.4 | 11 ± 0.4 | | < 5 | 70 ± 1.9 | | 16 ± 0.8 | | 82 ± 1.2 | 33 ± 0.9 | | < 5 | < 5 |
| **LG-1853** | 44 ± 0.9 | 12 ± 0.4 | 7 ± 0.1 | | < 5 | 50 ± 4.8 | | 8 ± 1.3 | | 73 ± 3.9 | 21 ± 1.5 | | < 5 | < 5 |

^1^ The results are expressed as the mean of at least three experiments

**Table S4** *In vitro* reactivation rates of OP inhibited *h*BChE at both concentrations. The *h*BChE activity was measured after 10 min incubation of the compound with OP-inhibited *h*BChE.

|  | **% recovery of OP-inhibited *h*BChE ± SD** | | | | | | | | | | | | | |
| --- | --- | --- | --- | --- | --- | --- | --- | --- | --- | --- | --- | --- | --- | --- |
|  | **GB** | | **GA** | | | **VX** | | | **PXE** | | | **PXM** | | |
|  | **100 µM** | **10 µM** | | **100 µM** | **10 µM** | | **100 µM** | **10 µM** | | **100 µM** | **10 µM** | | **100 µM** | **10 µM** |
| **LüH-6** | 21 ± 0.8 | 6 ±0.2 | | < 5 | < 5 | | 16 ± 0.3 | < 5 | | < 5 | < 5 | | 8 ± 0.5 | < 5 |
| **2PAM** | 27 ± 0.6 | < 5 | | < 5 | < 5 | | 21 ± 0.6 | 6 ± 0.2 | | 7 ± 0.3 | < 5 | | < 5 | < 5 |
| **HI-6** | 26 ± 0.4 | < 5 | | < 5 | < 5 | | 14 ± 0.4 | < 5 | | < 5 | < 5 | | < 5 | < 5 |
| **MMB4** | 25 ± 0.4 | 8 ± 0.2 | | < 5 | < 5 | | 15 ± 0.1 | < 5 | | < 5 | < 5 | | < 5 | < 5 |
| **LG-1154** | 38 ± 1.8 | 12 ± 0.4 | | < 5 | < 5 | | 63 ± 0.7 | 26 ± 0.4 | | 34 ± 0.7 | 7.1 ± 0.4 | | 34 ± 1.0 | 8 ± 0.4 |
| **LG-1795** | 72 ± 6.3 | 31 ± 0.7 | | < 5 | < 5 | | 72 ± 3.7 | 67 ± 0.9 | | 75 ± 2.0 | 24 ± 0.6 | | 53 ± 7.7 | 60 ± 1.2 |
| **LG-1781** | 45 ± 0.9 | 8 ± 0.6 | | < 5 | < 5 | | 72 ± 0.8 | 21 ± 0.2 | | 7 ± 0.3 | < 5 | | 42 ± 1.6 | 6 ± 0.6 |
| **LG-1786** | 61 ± 1.0 | 16 ± 0.6 | | < 5 | < 5 | | 77 ± 0.1 | 28 ± 0.6 | | 57 ± 1.0 | 10 ± 0.7 | | 45 ± 0.6 | 10 ± 0.6 |
| **LG-1703** | < 5 | < 5 | | < 5 | < 5 | | 20 ± 0.6 | < 5 | | 9 ± 0.3 | < 5 | | 12 ± 0.8 | < 5 |
| **LG-1704** | < 5 | < 5 | | < 5 | < 5 | | 24 ± 0.4 | 6 ± 0.1 | | 10 ± 0.3 | < 5 | | 6 ± 0.8 | < 5 |
| **LG-1829** | 13 ± 0.4 | < 5 | | < 5 | < 5 | | 32 ± 0.8 | 6 ± 0.2 | | 14 ± 0.5 | < 5 | | 7 ± 0.9 | < 5 |
| **LG-1853** | 23 ± 1.9 | 11 ± 0.4 | | < 5 | < 5 | | 40 ± 0.4 | 8 ± 0.2 | | 9 ± 0.6 | < 5 | | 13 ± 0.1 | < 5 |

^1^ The results are expressed as the mean of at least three experiments

# Molecular dynamics to answer reactivation capacities

**Table S5** The molecular dynamics result overview shows for each ligand orientation the average (mean ± SD) oximate oxygen-GB phosphorus distance (O-P distance), the average distance (mean ± SD) between Ser-203 hydroxyl group and GB phosphorus (OG-P distance). The interaction energies are defined as the sum of short-range Lennard–Jones and Coulombic interaction energies (mean ± SD).

| **ligand** | **orientation mark closer to GB** | **average O-P distance [Å]** | **average OG-P distance [Å]** | **interaction energy between GB and the enzyme active site [kJ/mol]** | **interaction energy between the oxime and active site of GB-inhibited enzyme [kJ/mol]** | **interaction energy between the oxime and GB [kJ/mol]** |
| --- | --- | --- | --- | --- | --- | --- |
| **LüH-6** | A | 9.2 ± 0.2  9.5 ± 0.1 | 1.66 ± 0 | -648 ± 3 | -128 ± 4 | -6.1 ± 1 |
| **2-PAM** | A | 8.7 ± 2.5 | 1.6 ± 0 | -659 ± 3 | -68 ± 2 | -1.9 ± 1.2 |
| **HI-6** | A | 9.2 ± 2.8 | 1.6 ± 0 | -664 ± 2 | -151 ± 5 | -5.0 ± 1.1 |
| **LG-1154** | A | 11.3 ± 2.8  14.9 ± 0.4 | 1.58 ± 0.04 | -662 ± 3 | -180 ± 10 | -3.0 ± 1.5 |
|  | B | 11.9 ± 0.2  10.6 ± 1.3 | 1.58 ± 0.04 | -660 ± 3 | -145 ± 6 | -3.2 ± 0.9 |
| **LG-1795** | A | 8.7 ±0.8  11 ± 1.0 | 1.59 ±0.04 | -652 ± 3 | -197 ± 6 | -12.4 ± 0.7 |
|  | B | 14.5 ± 3.2  11.2 ± 1.2 | 1.58 ± 0.04 | -655 ± 3 | -181 ± 5 | -6.8 ± 1.4 |
| **LG-1781** | A | 7.4 ± 1.5  16.8 ± 2.1 | 1.59 ± 0.04 | -655 ± 3 | -144 ± 3 | -7.7 ±0.9 |
|  | B | 13.8 ± 2.7  12.7 ± 4.7 | 1.59 ± 0.04 | -636 ± 4 | -169 ± 5 | -2.8 ± 0.7 |
| **LG-1786** | A | 8.6 ± 1.8  14.3 ± 1.0 | 1.58 ± 0.04 | -652 ± 3 | -154 ± 7 | -6.6 ± 0.5 |
|  | B | 13.9 ± 2.0  10.4 ± 2.9 | 1.58 ± 0.04 | -652 ± 2 | -152 ± 2 | -4.2 ± 0.6 |
| **LG-1703** | A | 14.9 ± 4.8  16.1 ± 3.9 | 1.58 ± 0.04 | -660 ± 3 | -240 ± 14 | -2.0 ± 1.5 |
|  | B | 21.7 ± 3.1  7.1 ± 2.0 | 1.6 ± 0.04 | -603 ± 3 | -213 ± 10 | -1.0 ± 0.6 |
| **LG-1704** | A | No molecular dynamics simulation was RMSD valid. | | | | |
|  | B | 16.5 ± 2.5  12 ± 1.3 | 1.58 ± 0.04 | -666 ± 2 | -237 ± 17 | -3.4 ± 0.3 |
| **LG-1853** | A | 11 ± 3.9  12.4 ± 2.7 | 1.58 ± 0.04 | -653 ± 5 | -151 ± 10 | -4.3 ±1.3 |
|  | B | 20.1 ± 1.2  13.8 ± 2.4 | 1.58 ± 0.04 | -663 ± 2 | -202 ± 8 | -0.8 ± 0.3 |
| **LG-1829** | A | 11.4 ± 2.1  17.4 ± 2.6 | 1.58 ± 0.04 | -663 ± 4 | -170 ± 6 | -3.0 ± 0.5 |
|  | B | 18.5 ± 2.5  11 ± 2 | 1.58 ± 0.04 | -660 ± 4 | -192 ± 6 | -2.3 ± 0.3 |

**Table S6** The molecular dynamics result overview shows for each ligand orientation the average (mean ± SD) oximate oxygen-VX phosphorus distance (O-P distance), the average distance (mean ± SD) between Ser-203 hydroxyl group and VX phosphorus (OG-P distance). The interaction energies are defined as the sum of short-range Lennard–Jones and Coulombic interaction energies (mean ± SD).

| **ligand** | **orientation mark closer to VX** | **average O-P distance [Å]** | **average OG-P distance [Å]** | **interaction energy between VX and the enzyme active site [kJ/mol]** | **interaction energy between the oxime and active site of VX-inhibited enzyme [kJ/mol]** | **interaction energy between the oxime and VX [kJ/mol]** |
| --- | --- | --- | --- | --- | --- | --- |
| **LüH-6** | A | 9.6 ± 2.3  14.3 ± 2.2 | 1.59 ± 0.004 | -654 ± 3 | -123 ± 3 | -3.2 ± 0.4 |
| **2-PAM** | A | 7.4 ± 0.8 | 1.59 ± 0.004 | -658 ± 1 | -73 ± 2 | -4.8 ± 3.6 |
| **HI-6** | A | 9.8 ± 2.1 | 1.59 ± 0.004 | -660 ± 1 | -138 ± 4 | -2.5 ± 0.2 |
| **LG-1154** | A | 7.5 ± 1.9  14.8 ± 2.6 | 1.59 ± 0.004 | -649 ± 2 | -147 ± 7 | -4.7 ± 0.4 |
|  | B | 16.8 ± 2.0  10.2 ± 3.6 | 1.59 ± 0.004 | -611 ± 3 | -153 ± 9 | -0.03 ± 0.53 |
| **LG-1795** | A | 13.5 ± 3.3  17.2 ± 2.0 | 1.58 ± 0.004 | -658 ± 1 | -213 ± 18 | -0.5 ± 0.2 |
|  | B | 20.3 ± 4.4  10.7 ± 3.0 | 1.58 ± 0.004 | -652 ± 7 | -168 ± 8 | -0.9 ± 0.6 |
| **LG-1781** | A | 8.8 ± 1.8  16.5 ± 3.0 | 1.59 ± 0.004 | -652 ± 2 | -158 ± 7 | -4.1 ± 0.6 |
|  | B | 15.7 ± 4.1  5.2 ± 0.5 | 1.59 ± 0.004 | -657 ± 9 | -137 ± 3 | -7.6 ± 0.4 |
| **LG-1786** | A | 7.2 ± 1.9  11.2 ± 0.9 | 1.59 ± 0.004 | -639 ± 2 | -164 ± 6 | -10 ± 0.7 |
|  | B | 13.7 ± 2.3  10 ± 1.3 | 1.59 ± 0.004 | -660 ± 2 | -173 ± 9 | -6.2 ± 0.8 |
| **LG-1703** | A | 8 ± 2.2  11.6 ± 1.6 | 1.59 ± 0.004 | -648 ± 3 | -269 ± 8 | -3.7 ± 0.6 |
|  | B | 20.3 ± 1.8  5.3 ± 1.8 | 1.59 ± 0.004 | -654 ± 4 | -231 ± 9 | -5.7 ± 0.5 |
| **LG-1704** | A | 13 ± 4.8  13.6 ± 2.9 | 1.59 ± 0.003 | -656 ± 6 | -158 ± 9 | -4 ± 0.4 |
|  | B | 15.5 ± 3.1  12.8 ± 1.5 | 1.59 ± 0.004 | -658 ± 3 | -224 ± 13 | -1.7 ± 0.4 |
| **LG-1853** | A | 4.5 ± 1.2  17.6 ± 2.2 | 1.59 ± 0.004 | -658 ± 2 | -194 ± 9 | -9 ± 0.5 |
|  | B | 17.1 ± 1.2  14.6 ± 2.9 | 1.59 ± 0.004 | -645 ± 1 | -210 ± 21 | -0.3 ± 0.1 |
| **LG-1829** | A | 7.9 ± 1.7  15.8 ± 2.9 | 1.59 ± 0.004 | -657 ± 5 | -205 ± 12 | -6.5 ± 1 |
|  | B | 13.5 ± 2.8  11.4 ± 1.1 | 1.59 ± 0.004 | -658 ± 1 | -186 ± 16 | -5.3 ± 0.8 |

# Root mean square deviation (RMSD) for molecular dynamics

**Figure S1** RMSD values of **LüH-6** heavy atoms by least-square fitting to the backbone over time during molecular dynamics simulations of ligand with GB-inhibited *h*AChE structure.

**Figure S2** RMSD values of **2-PAM** heavy atoms by least-square fitting to the backbone over time during molecular dynamics simulations of ligand with GB-inhibited *h*AChE structure.

**Figure S3** RMSD values of **HI-6** heavy atoms by least-square fitting to the backbone over time during molecular dynamics simulations of ligand with GB-inhibited *h*AChE structure.

**Figure S4** RMSD values of **LG-1154** heavy atoms by least-square fitting to the backbone over time during molecular dynamics simulations of ligand with GB-inhibited *h*AChE structure.

**Figure S5** RMSD values of **LG-1795** heavy atoms by least-square fitting to the backbone over time during molecular dynamics simulations of ligand with GB-inhibited *h*AChE structure.

**Figure S6** RMSD values of **LG-1781** heavy atoms by least-square fitting to the backbone over time during molecular dynamics simulations of ligand with GB-inhibited *h*AChE structure.

**Figure S7** RMSD values of **LG-1786** heavy atoms by least-square fitting to the backbone over time during molecular dynamics simulations of ligand with GB-inhibited *h*AChE structure.

**Figure S8** RMSD values of **LG-1703** heavy atoms by least-square fitting to the backbone over time during molecular dynamics simulations of ligand with GB-inhibited *h*AChE structure.

**Figure S9** RMSD values of **LG-1704** heavy atoms by least-square fitting to the backbone over time during molecular dynamics simulations of ligand with GB-inhibited *h*AChE structure.

**Figure S10** RMSD values of **LG-1853** heavy atoms by least-square fitting to the backbone over time during molecular dynamics simulations of ligand with GB-inhibited *h*AChE structure.

**Figure S11** RMSD values of **LG-1829** heavy atoms by least-square fitting to the backbone over time during molecular dynamics simulations of ligand with GB-inhibited *h*AChE structure.

**Figure S12** RMSD values of **LüH-6** heavy atoms by least-square fitting to the backbone over time during molecular dynamics simulations of ligand with VX-inhibited *h*AChE structure.

**Figure S13** RMSD values of **2-PAM** heavy atoms by least-square fitting to the backbone over time during molecular dynamics simulations of ligand with VX-inhibited *h*AChE structure.

**Figure S14** RMSD values of **HI-6** heavy atoms by least-square fitting to the backbone over time during molecular dynamics simulations of ligand with VX-inhibited *h*AChE structure.

**Figure S15** RMSD values of **LG-1154** heavy atoms by least-square fitting to the backbone over time during molecular dynamics simulations of ligand with VX-inhibited *h*AChE structure.

**Figure S16** RMSD values of **LG-1795** heavy atoms by least-square fitting to the backbone over time during molecular dynamics simulations of ligand with VX-inhibited *h*AChE structure.

**Figure S17** RMSD values of **LG-1781** heavy atoms by least-square fitting to the backbone over time during molecular dynamics simulations of ligand with VX-inhibited *h*AChE structure.

**Figure S18** RMSD values of **LG-1786** heavy atoms by least-square fitting to the backbone over time during molecular dynamics simulations of ligand with VX-inhibited *h*AChE structure.

**Figure S19** RMSD values of **LG-1703** heavy atoms by least-square fitting to the backbone over time during molecular dynamics simulations of ligand with VX-inhibited *h*AChE structure.

**Figure S20** RMSD values of **LG-1704** heavy atoms by least-square fitting to the backbone over time during molecular dynamics simulations of ligand with VX-inhibited *h*AChE structure.

**Figure S21** RMSD values of **LG-1853** heavy atoms by least-square fitting to the backbone over time during molecular dynamics simulations of ligand with VX-inhibited *h*AChE structure.

**Figure S22** RMSD values of **LG-1829** heavy atoms by least-square fitting to the backbone over time during molecular dynamics simulations of ligand with VX-inhibited *h*AChE structure.

# Degradation of GB, VX and A234 by selected reactivators

**Figure S23** Time curve degradation of (A) GB in PBS by selected reactivators in 1:1 (GB to reactivator, molar concentration) ratio with or without reactivator, (B) GB in PBS by selected reactivators in 1:5 (GB to reactivator, molar concentration) ratio with or without reactivator. (C) GB in PBS by selected reactivators in 1:10 (GB to reactivator, molar concentration) ratio with or without reactivator at the time range of 120 min. The results are expressed as mean ± SD (n = 3).

# LC analysis of compounds stability


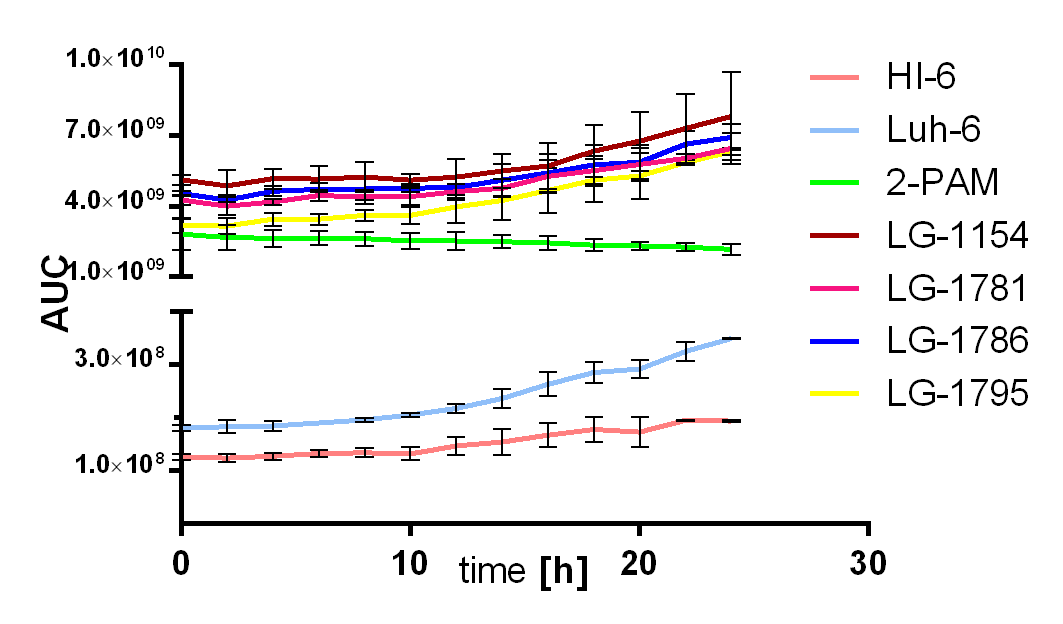


**Figure S24** LC analysis of compounds stability in PBS (pH = 7.4) at laboratory temperature, evaluated using LC-MS techniques.

# *In vivo* toxicity study

**Table S7** Maximum Tolerated Dose (MTD) study of LG-1154, LG-1781, LG-1786, and LG-1795 (i.m.; 10% Kolliphor/PBS, volume 0.1 ml/kg), Balb/c mice.

| **LG-1154** | | | **LG-1781** | | | **LG-1786** | | |
| --- | --- | --- | --- | --- | --- | --- | --- | --- |
| **Dose mg/kg** | **Survived/treated** | **Symptoms** | **Dose mg/kg** | **Survived/ treated** | **Symptoms** | **Dose mg/kg** | **Survived/treated** | **Symptoms** |
| 200 | 0/4  2♀ and 2♂ | Breathing changes (+++), tremor (++/+++), activity reduction (+), and half-closed eyes were observed after administration (both sexes). Convulsions were observed in one ♀.  All animals died (13´,15´,20´,27´). | 100 | 8/8  4♀ and 4♂ | All ♀ survived. Breathing changes (++), tremor (+), and activity reduction (+/0) were observed. All symptoms disappeared during one hour.  All ♂ survived. Only tremor (++/+/0), and activity reduction (+) were observed. All symptoms disappeared during one hour.  **Maximum Tolerated Dose (MTD).** | 200 | 0/4  2♀ and 2♂ | Breathing changes (+++), tremor (++), convulsions (+++), activity reduction (+), and half-closed eyes were observed after administration (both sexes).  All animals died (20´, 25´, 26´,30´). |
| 100 | 4/6 2♀ and 4♂ | Both ♀ died (16´, 35´). Breathing changes (+++), tremor (++/+++), activity reduction (+), and half closed eyes were observed before death. Convulsions were observed in one ♀.  All ♂ survived. Only tremor (++/+), activity reduction (+), and half-closed eyes were observed. All symptoms disappeared during one hour.  **Maximum Tolerated Dose (MTD in males).** |  |  |  | 100 | 2/6  2♀ and 4♂ | Both ♀ died (23´, 32´). Breathing changes (+++), tremor (+++), convulsions (++), activity reduction (+), and half-closed eyes were observed.  Two ♂ died (12´, 15´). Breathing changes (+++/++), tremor (++), activity reduction (+), and half-closed eyes were observed. Convulsions were observed in one ♂. |
| 50 | 4/4  4♀ | All ♀ survived. Only tremor (+), activity reduction (+), and half-closed eyes were observed. All symptoms disappeared during one hour.  **Maximum Tolerated Dose (MTD in females).** |  |  |  | 50 | 8/8  4♀ and 4♂ | All ♀ survived. Breathing changes (++), tremor (+), and activity reduction (+) were observed. All symptoms disappeared during one hour.  All ♂ survived. Breathing changes (++), tremor (+), and activity reduction (+) were observed. All symptoms disappeared during one hour.  **Maximum Tolerated Dose (MTD).** |

| **LG-1795** | | |  | | |  | | |
| --- | --- | --- | --- | --- | --- | --- | --- | --- |
| **Dose mg/kg** | **Survived/treated** | **Symptoms** |  |  |  |  |  |  |
| 100 | 8/8  4♀ and 4♂ | All ♀ survived. Only activity reduction (+), and half-closed eyes were observed. Tremor (+) was observed in one ♀. All symptoms disappeared during one hour.  All ♂ survived. Only activity reduction (+) were observed. Tremor (+) was observed in two ♂. All symptoms disappeared during one hour.  **Maximum Tolerated Dose (MTD).** |  |  |  |  |  |  |

**Table S8** Histopathological findings in the kidney and liver of mice administered with **LG-1154**, **LG-1781**, **LG-1786**, and **LG-1795** at doses corresponding with MTDs.

|  |  | **dose (MTD, mg/kg)** | **kidney** | **liver** |
| --- | --- | --- | --- | --- |
| **Control ^a^** | **males (N = 2)** | **0** | **0** | **0** |
|  | **females (N = 2)** | **0** | **0** | **0** |
| **LG-1154** | **males (N = 4)** | **100** | **0** | **0** |
|  | **females (N = 4)** | **50** | **0** | **0** |
| **LG-1781** | **males (N = 4)** | **100** | **0** | **0** |
|  | **females (N = 4)** | **100** | **0** | **0** |
| **LG-1786** | **males (N = 4)** | **50** | **0** | **0** |
|  | **females (N = 4)** | **50** | **0** | **0** |
| **LG-1795** | **males (N = 4)** | **100** | **0** | **0** |
|  | **females (N = 4)** | **100** | **0** | **0** |

Histopathological findings: 0 (none), + (mild), ++ (moderate), and +++ (severe). ^a^ Control group received 10% kolliphor/PBS (V/V).

# Functional Observational Battery

**Table S9** Keying table to Functional Observational Battery studies, scored values from Tables S10-18.

| **Marker** | **Scored values** | | | | | | | | | |
| --- | --- | --- | --- | --- | --- | --- | --- | --- | --- | --- |
|  | **-2** | **-1** | **0** | **1** | **2** | **3** | **4** | **5** | **6** | **7** |
| **posture** |  |  |  | ***sitting or standing*** | ***rearing*** | ***asleep*** | flattened | lying on side | crouched over | head bobbing |
| **catch difficulty** |  |  |  | passive | ***normal*** | defense | flight | escape | aggression |  |
| **ease of handling** |  |  |  | very easy | ***easy*** | moderately difficult | difficult |  |  |  |
| **respiration** | apnoe | bradypnoe | ***normal*** | tachypnoe | dyspnoe |  |  |  |  |  |
| **muscular tonus** | atonic | hypotonic | ***normal*** | hypertonic | rigidity | fasciculation |  |  |  |  |
| **lacrimation** |  |  | ***none*** | slight | severe | crusts | colored crusts |  |  |  |
| **lids position** |  |  |  | ***open*** | Slightly dropping | half-way dropping | completely shut | ptosis |  |  |
| **endo/exophthalmos** |  | endophtalmos | ***normal*** | exophthalmos |  |  |  |  |  |  |
| **fur abnormalities** |  |  | ***normal*** | colored | disheveled | colored + disheveled | baldness | injury | other changes | piloerection |
| **skin abnormalities** |  |  | ***normal*** | pale | erythema | cyanosis | pigmentation | cold | injury |  |
| **salivation** |  |  | ***none*** | slight | severe |  |  |  |  |  |
| **nose secretion** |  |  | ***none*** | slight | severe | colored |  |  |  |  |
| **hyperkinesis** |  |  | ***none*** | repetitive movements of mouth and jaws | non-rhythmic quivers | mild tremors | severe tremors | myoclonic jerks | clonic convulsion |  |
| **tremors** |  |  | ***none*** | only when stimulated | mild local | mild overall | moderate – affecting movement | strong – defending movement |  |  |
| **clonic movements** |  |  | ***none*** | twitching | non-rhythmic movement |  |  |  |  |  |
| **tonic movements** |  |  | ***normal*** | contraction of extensors | opistotonus | emprostotonus | explosive jumps | tonic convulsions |  |  |
| **ataxia** |  |  | ***none*** | slight | severe |  |  |  |  |  |
| **paresis** |  |  | ***none*** | slight | severe |  |  |  |  |  |
| **gait** |  |  | ***normal*** | ataxia | overcompensation of hind limb movements | feet point outwards from body | forelimbs are extended | walks on tiptoes | hunched body | body is flattened against surface |
| **total disability score** |  |  |  | ***normal*** | slightly impaired | somewhat impaired | totally impaired |  |  |  |
| **mobility score** |  |  |  | ***normal*** | slightly impaired | somewhat impaired | totally impaired |  |  |  |
| **activity** |  |  |  | very low | sporadic | reduced | ***normal*** | enhanced | permanent |  |
| **tension** |  |  | ***none*** | partial (ears) | stupor |  |  |  |  |  |
| **vocalization** |  |  | ***none*** | provoked | spontaneous | excessive |  |  |  |  |
| **stereotypy** |  |  | ***none*** | head weaving | body weaving | grooming | circling | others |  |  |
| **bizarre behavior** |  |  | ***none*** | head | body | self-mutilation | abnormal movements | others |  |  |
| **approach response** |  |  |  | no reaction | ***normal*** | increased reaction/ stiffening | energetic reaction | exaggerated reaction |  |  |
| **touch response** |  |  |  | no reaction | ***normal*** | increased reaction/ stiffening | energetic reaction | exaggerated reaction |  |  |
| **click response** |  |  |  | no reaction | ***normal*** | increased reaction/ stiffening | energetic reaction | exaggerated reaction |  |  |
| **tail-pinch response** |  |  |  | no reaction | ***normal*** | increased reaction/ stiffening | energetic reaction | exaggerated reaction |  |  |
| **pupil size** | strong miosis | miosis | ***normal*** | mydriasis | strong mydriasis |  |  |  |  |  |
| **pupil response** |  | no reaction | slow reaction | ***normal reaction*** |  |  |  |  |  |  |
| **righting reflex from back or vertical positions** |  |  |  | ***normal*** | slightly uncoordinated | lands on side | lands on back |  |  |  |

**Table S10** Overview of markers defining activity and neuromuscular functions affected by sarin (GB), with or without the applied treatment strategy, assessed 2 or 24 hours after administration (n = 9)

**Table S11** Overview of markers defining sensorimotor and neurotoxic excitability functions affected by sarin (GB), with or without the applied treatment strategy, assessed 2 or 24 hours after administration (n = 9).

**Table S12** Overview of markers defining autonomic neurotoxic functions affected by sarin (GB), with or without the applied treatment strategy, assessed 2 or 24 hours after administration (n = 9).

**Table S13** Overview of markers defining activity and neuromuscular functions affected by compound VX, with or without the applied treatment strategy, assessed 2 or 24 hours after administration (n = 9)

**Table S14** Overview of markers defining sensorimotor and neurotoxic excitability functions affected by compound VX, with or without the applied treatment strategy, assessed 2 or 24 hours after administration (n = 9).

**Table S15** Overview of markers defining autonomic neurotoxic functions affected by compound VX, with or without the applied treatment strategy, assessed 2 or 24 hours after administration (n = 9).

**Table S16** Overview of markers defining activity and neuromuscular functions affected by paraoxon (PXE), with or without the applied treatment strategy, assessed 2 or 24 hours after administration (n = 9)

**Table S17** Overview of markers defining sensorimotor and neurotoxic excitability functions affected by paraoxon (PXE), with or without the applied treatment strategy, assessed 2 or 24 hours after administration (n = 9).

**Table S18** Overview of markers defining autonomic neurotoxic functions affected by paraoxon (PXE), with or without the applied treatment strategy, assessed 2 or 24 hours after administration (n = 9).

# Chemical synthesis

## Preparation of key intermediates

**6‐(5‐bromopentyl)‐2‐[(E)‐(hydroxyimino)methyl]pyridin‐3‐ol (5):** Prepared according to already published procedure [1]. Overall yield ≈ 20% for nine steps. ^1^H NMR (500 MHz, CDCl_3_-*d*) δ 9.97 – 9.80 (m, 1H), 8.47 (s, 1H), 7.24 (d, *J* = 4.9 Hz, 1H), 7.06 (d, *J* = 8.5 Hz, 1H), 3.36 (t, *J* = 6.8 Hz, 2H), 2.77 – 2.70 (m, 2H), 1.90 – 1.81 (m, 2H), 1.73 – 1.65 (m, 2H), 1.52 – 1.40 (m, 2H). ^13^C NMR (126 MHz, CDCl_3_) δ 153.50, 153.20, 152.83, 134.45, 125.41, 124.46, 36.71, 33.71, 32.56, 29.28, 27.75.

**(*E*)‐*N*‐[(1‐methylimidazol‐2‐yl)methylidene]hydroxylamine (6):** Prepared according to already published procedure [2]. ^1^H NMR (500 MHz, DMSO-*d*_6_) δ 11.48 (s, 1H), 8.05 (s, 1H), 7.25 (d, *J* = 1.1 Hz, 1H), 6.98 (d, *J* = 1.1 Hz, 1H), 3.80 (s, 3H). ^13^C NMR (126 MHz, DMSO) δ 141.55, 140.28, 128.70, 124.75, 34.92. HRMS (ESI^+^): [M+H]^+^: calculated for C_5_H_8_N_3_O^+^: 126.06619; detected: 126.06583. LC-UV purity >99%.

**(*E*)‐*N*‐(isoquinolin‐5‐ylmethylidene)hydroxylamine (7):** Prepared according to already published procedure [2]. ^1^H NMR (500 MHz, DMSO-*d*_6_) δ 11.61 (s, 1H), 9.36 (d, *J* = 1.1 Hz, 1H), 8.76 (s, 1H), 8.59 – 8.50 (m, 2H), 8.19 – 8.11 (m, 1H), 8.07 – 8.00 (m, 1H), 7.74 – 7.67 (m, 1H). ^13^C NMR (126 MHz, DMSO) δ 153.52, 148.06, 144.36, 132.77, 131.30, 129.74, 129.04, 128.49, 127.60, 118.15. HRMS (ESI^+^): [M+H]^+^: calculated for C_10_H_9_N_2_O^+^: 173.07094; detected: 173.06989. LC-UV purity 98%.

## Final *N*‑alkylation coupling

**3‐(5‐{5‐hydroxy‐6‐[(*E*)‐(hydroxyimino)methyl]pyridin‐2‐yl}pentyl)‐2‐[(*E*)‐(hydroxyimino)methyl]‐1‐methyl‐1*H*‐imidazol‐3‐ium bromide (1; LG1154):** Prepared according to already published procedure [1, 2]. The compound **5** (550 mg; 1.92 mmol) and the compound **6** (360 mg; 2.88 mmol) were mixed in anhydrous acetonitrile (MeCN; 10 mL). The mixture was subjected to MW irradiation with the following settings: dynamic curve, maximum power 100 W, maximum pressure cap 300 PSI, and 90 °C for 24 hours. The solvent was evaporated under reduced pressure, and the residue purified by column chromatography with mobile phase DCM/MeOH (5:1) to give pure product **1** as a white powder. Yield 44%. ^1^H NMR (500 MHz, CD_3_OD-*d*_4_) δ 8.49 (s, 1H), 8.27 (s, 1H), 7.74 (d, *J* = 2.0 Hz, 1H), 7.67 (d, *J* = 2.0 Hz, 1H), 7.28 (d, *J* = 8.5 Hz, 1H), 7.18 (d, *J* = 8.5 Hz, 1H), 4.44 – 4.34 (m, 2H), 4.01 (s, 3H), 2.77 – 2.70 (m, 2H), 1.95 – 1.85 (m, 2H), 1.79 – 1.69 (m, 2H), 1.43 (p, *J* = 7.6 Hz, 2H). ^13^C NMR (126 MHz, CD_3_OD) δ 154.36, 153.77, 152.62, 138.65, 136.13, 135.53, 126.19, 125.72, 125.54, 124.55, 50.69, 37.36, 37.32, 30.58, 30.32, 26.59. HRMS (ESI^+^): [M+2H]^2+^: calculated for C_16_H_23_N_5_O_3_^2+^: 166.58950; detected: 166.58937. LC-UV purity 97%.

**1‐(5‐{5‐hydroxy‐6‐[(*E*)‐(hydroxyimino)methyl]pyridin‐2‐yl}pentyl)‐4‐[(*E*)‐(hydroxyimino)methyl]pyridin‐1‐ium bromide (2; LG1781):** Prepared according to already published procedure [1, 2]. The compound **5** (541 mg; 1.88 mmol) and pyridine-4-aldoxime (345 mg; 2.82 mmol) were mixed in anhydrous acetonitrile (MeCN; 10 mL). The mixture was subjected to MW irradiation with the following settings: dynamic curve, maximum power 100 W, maximum pressure cap 300 PSI, and 90 °C for 24 hours. The solvent was evaporated under reduced pressure, and the residue purified by column chromatography with mobile phase DCM/MeOH (7:1) to give pure product **2** as a white powder. Yield 57%. ^1^H NMR (500 MHz, CD_3_OD-*d*_4_) δ 8.95 – 8.89 (m, 2H), 8.32 (s, 1H), 8.27 (s, 1H), 8.24 – 8.18 (m, 2H), 7.27 (d, *J* = 8.5 Hz, 1H), 7.17 (d, *J* = 8.5 Hz, 1H), 4.60 (t, *J* = 7.6 Hz, 2H), 2.74 (t, *J* = 7.6 Hz, 2H), 2.06 (p, *J* = 7.6 Hz, 2H), 1.77 (p, *J* = 7.6 Hz, 2H), 1.46 – 1.38 (m, 2H). ^13^C NMR (126 MHz, CD_3_OD) δ 152.83, 152.43, 151.36, 149.79, 144.56, 144.17, 134.90, 124.68, 124.07, 124.05, 60.94, 35.91, 30.63, 28.96, 25.08. HRMS (ESI^+^): [M+2H]^2+^: calculated for C_17_H_22_N_4_O_3_^2+^: 165.08405; detected: 165.08408. LC-UV purity 95%.

**1‐(5‐{5‐hydroxy‐6‐[(*E*)‐(hydroxyimino)methyl]pyridin‐2‐yl}pentyl)‐3‐[(*E*)‐(hydroxyimino)methyl]pyridin‐1‐ium bromide (3; LG1786):** Prepared according to already published procedure [1, 2]. The compound **5** (515 mg; 1.8 mmol) and pyridine-3-aldoxime (329 mg; 2.7 mmol) were mixed in anhydrous acetonitrile (MeCN; 10 mL). The mixture was subjected to MW irradiation with the following settings: dynamic curve, maximum power 100 W, maximum pressure cap 300 PSI, and 90 °C for 24 hours. The resulting residue was cooled to 0 °C and the precipitate was filtered to give pure product **3** as a white powder. Yield 78%. ^1^H NMR (500 MHz, CD_3_OD-*d*_4_) δ 9.22 – 9.16 (m, 1H), 8.97 – 8.92 (m, 1H), 8.78 – 8.72 (m, 1H), 8.29 – 8.24 (m, 2H), 8.12 – 8.05 (m, 1H), 7.27 (d, *J* = 8.5 Hz, 1H), 7.17 (d, *J* = 8.5 Hz, 1H), 4.65 (t, *J* = 7.6 Hz, 2H), 2.75 (t, *J* = 7.6 Hz, 2H), 2.08 (p, *J* = 7.6 Hz, 2H), 1.78 (p, *J* = 7.6 Hz, 2H), 1.50 – 1.39 (m, 2H). ^13^C NMR (126 MHz, CD_3_OD) δ 152.81, 152.44, 151.30, 143.88, 142.32, 142.25, 141.40, 134.91, 134.90, 127.97, 124.74, 124.07, 61.79, 35.89, 30.71, 28.97, 25.12. HRMS (ESI^+^): [M+2H]^2+^: calculated for C_17_H_22_N_4_O_3_^2+^: 165.08405; detected: 165.08385. LC-UV purity 97%.

**2‐(5‐{5‐hydroxy‐6‐[(*E*)‐(hydroxyimino)methyl]pyridin‐2‐yl}pentyl)‐5‐[(*E*)‐(hydroxyimino)methyl]isoquinolin‐2‐ium bromide (4; LG1795):** Prepared according to already published procedure [1, 2]. The compound **5** (492 mg; 1.71 mmol) and the compound **7** (443 mg; 2.57 mmol) were mixed in anhydrous acetonitrile (MeCN; 10 mL). The mixture was subjected to MW irradiation with the following settings: dynamic curve, maximum power 100 W, maximum pressure cap 300 PSI, and 90 °C for 24 hours. The solvent was evaporated under reduced pressure, and the residue purified by column chromatography with mobile phase CHCl_3_/MeOH (5:1) to give pure product **4** as a white powder. Yield 62%. ^1^H NMR (500 MHz, DMSO-*d*_6_) δ 11.95 (s, 1H), 11.81 (s, 1H), 10.18 (d, *J* = 1.5 Hz, 1H), 10.08 (s, 1H), 9.18 – 9.12 (m, 1H), 8.95 – 8.87 (m, 2H), 8.52 – 8.44 (m, 2H), 8.12 – 8.06 (m, 1H), 7.24 (d, *J* = 8.4 Hz, 1H), 7.14 (d, *J* = 8.4 Hz, 1H), 4.73 (t, *J* = 7.6 Hz, 2H), 2.66 (t, *J* = 7.6 Hz, 2H), 2.12 – 2.01 (m, 2H), 1.74 – 1.63 (m, 2H), 1.41 – 1.31 (m, 2H). ^13^C NMR (126 MHz, DMSO) δ 152.96, 151.89, 151.29, 150.87, 146.99, 136.43, 136.07, 135.83, 134.35, 131.98, 131.42, 129.62, 128.51, 124.58, 124.34, 123.77, 61.02, 36.46, 30.67, 29.12, 25.50. HRMS (ESI^+^): [M+2H]^2+^: calculated for C_21_H_24_N_4_O_3_^2+^ : 190.09187; detected: 190.09082. LC-purity 98%.

# ^1^H and ^13^C NMR spectra for the final products

**3‐(5‐{5‐hydroxy‐6‐[(*E*)‐(hydroxyimino)methyl]pyridin‐2‐yl}pentyl)‐2‐[(*E*)‐(hydroxyimino)methyl]‐1‐methyl‐1*H*‐imidazol‐3‐ium bromide (1; LG1154):**


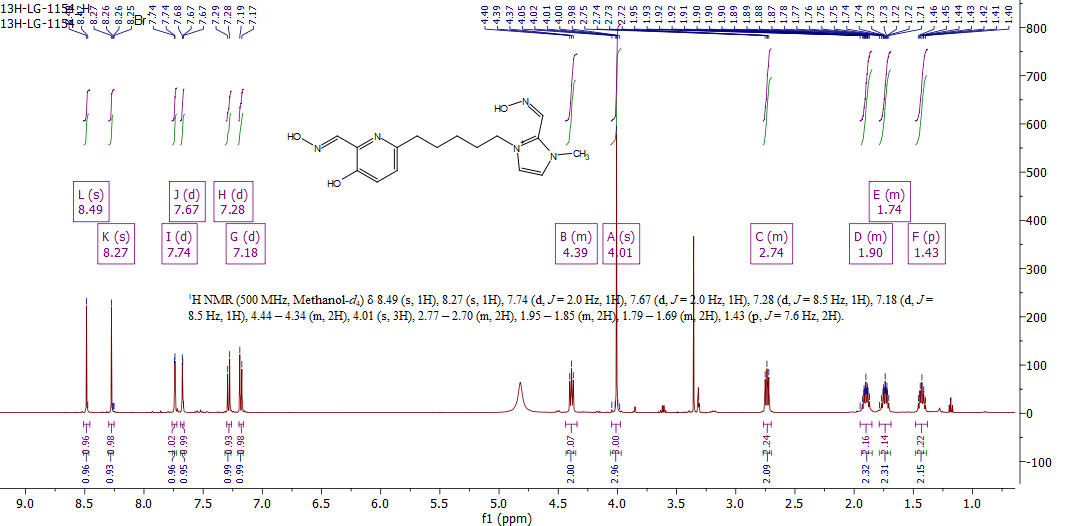


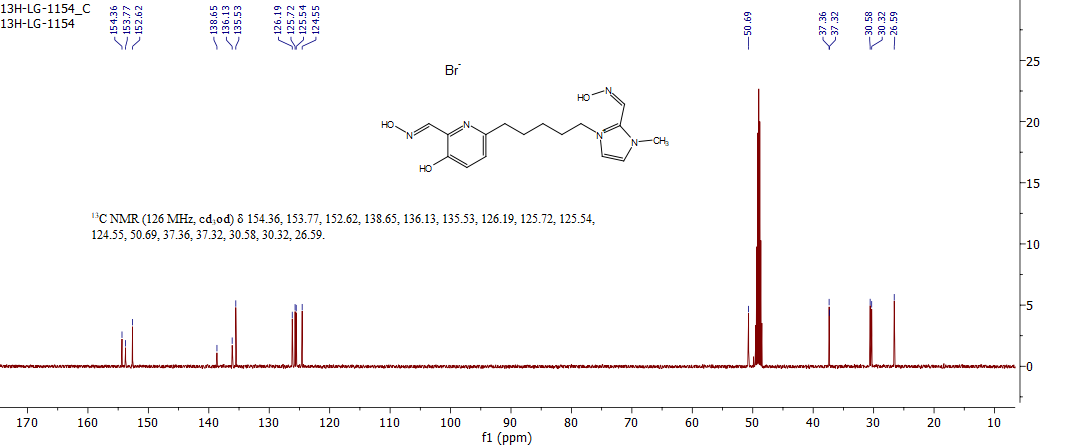


**1‐(5‐{5‐hydroxy‐6‐[(*E*)‐(hydroxyimino)methyl]pyridin‐2‐yl}pentyl)‐4‐[(*E*)‐(hydroxyimino)methyl]pyridin‐1‐ium bromide (2; LG1781):**


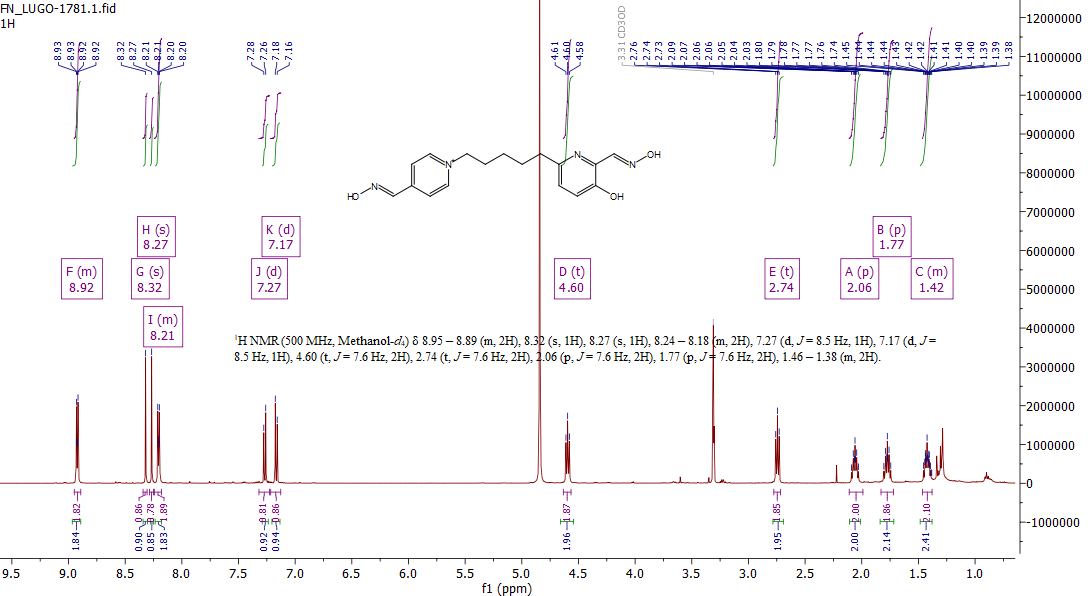


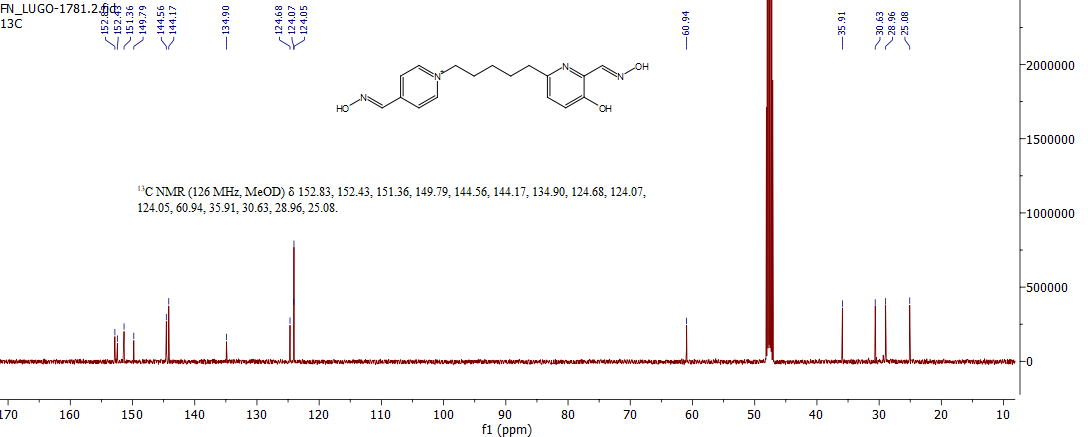


**1‐(5‐{5‐hydroxy‐6‐[(*E*)‐(hydroxyimino)methyl]pyridin‐2‐yl}pentyl)‐3‐[(*E*)‐(hydroxyimino)methyl]pyridin‐1‐ium bromide (3; LG1786):**


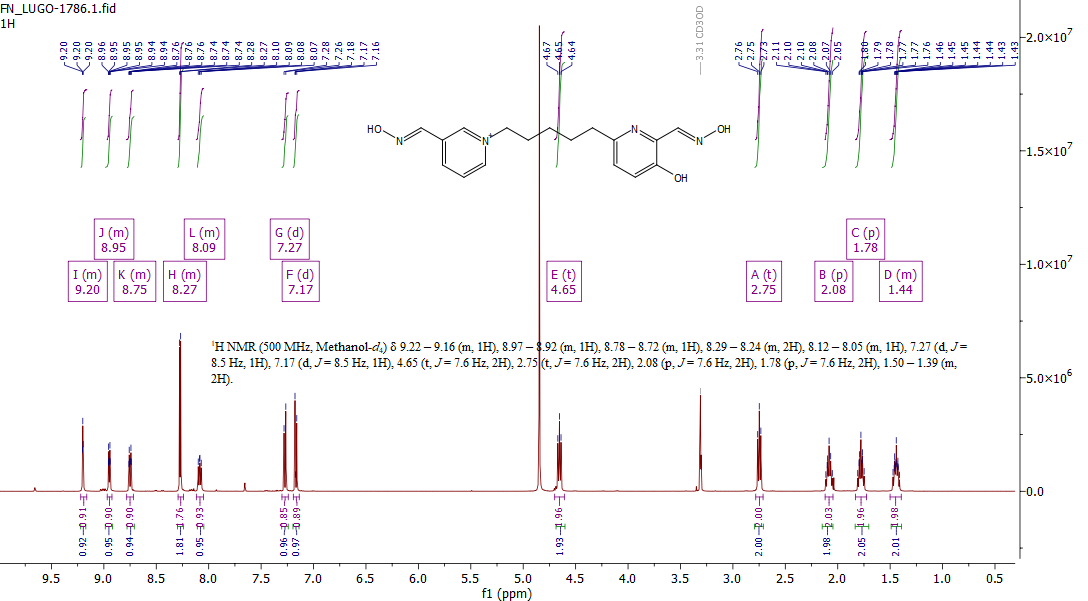


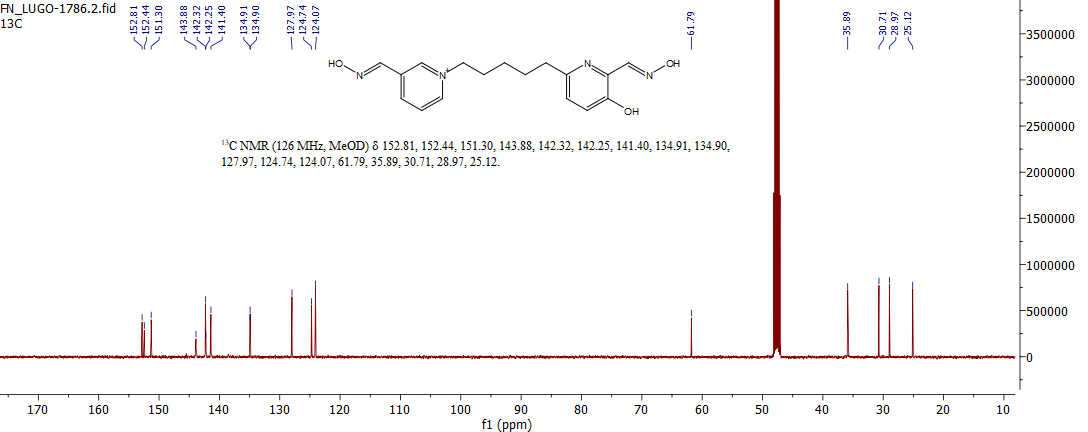


**2‐(5‐{5‐hydroxy‐6‐[(*E*)‐(hydroxyimino)methyl]pyridin‐2‐yl}pentyl)‐5‐[(*E*)‐(hydroxyimino)methyl]isoquinolin‐2‐ium bromide (4; LG1795):**


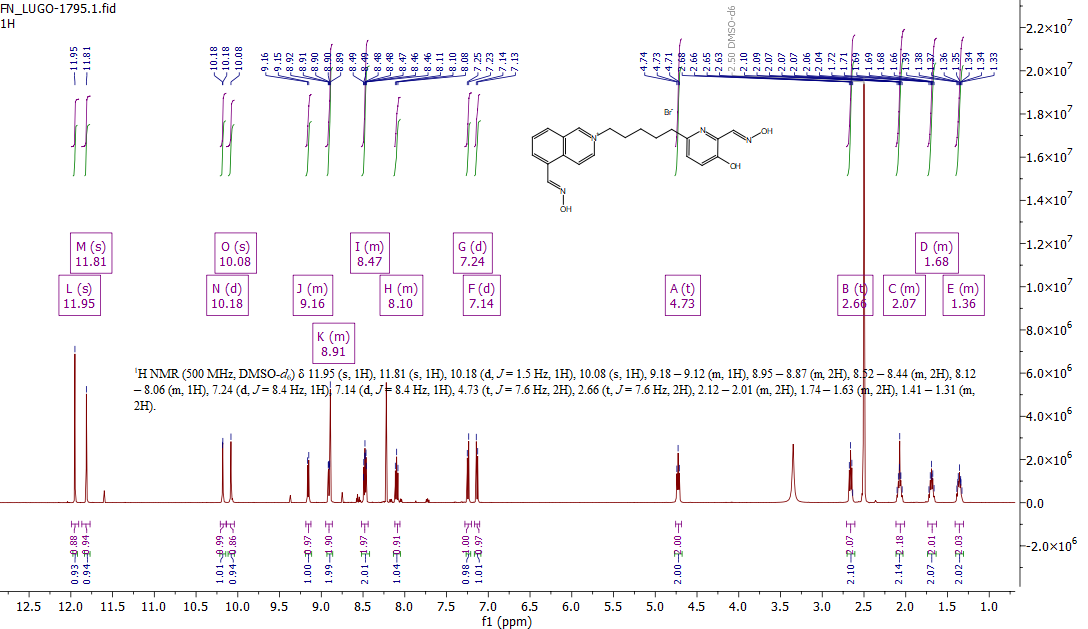


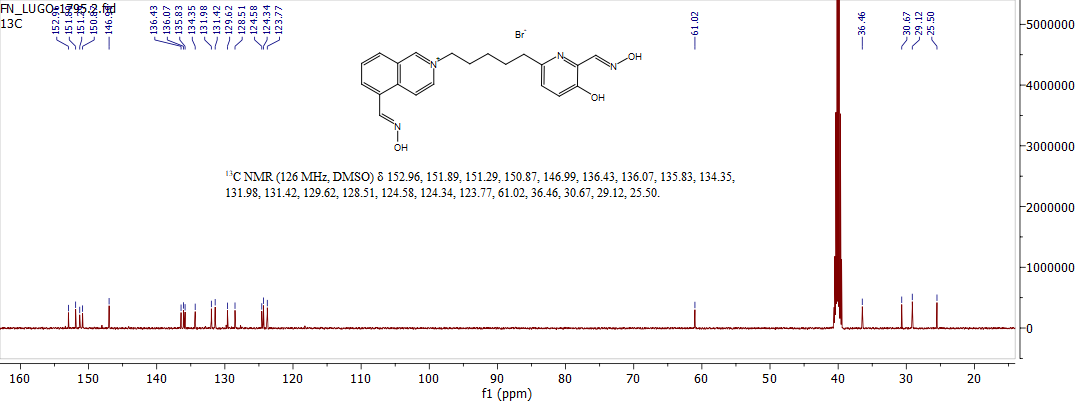


# LC-UV chromatograms and LC‑MS chromatograms for the final products

**3‐(5‐{5‐hydroxy‐6‐[(*E*)‐(hydroxyimino)methyl]pyridin‐2‐yl}pentyl)‐2‐[(*E*)‐(hydroxyimino)methyl]‐1‐methyl‐1*H*‐imidazol‐3‐ium bromide (1; LG1154):**

**1‐(5‐{5‐hydroxy‐6‐[(*E*)‐(hydroxyimino)methyl]pyridin‐2‐yl}pentyl)‐4‐[(*E*)‐(hydroxyimino)methyl]pyridin‐1‐ium bromide (2; LG1781):**

**1‐(5‐{5‐hydroxy‐6‐[(*E*)‐(hydroxyimino)methyl]pyridin‐2‐yl}pentyl)‐3‐[(*E*)‐(hydroxyimino)methyl]pyridin‐1‐ium bromide (3; LG1786):**

**2‐(5‐{5‐hydroxy‐6‐[(*E*)‐(hydroxyimino)methyl]pyridin‐2‐yl}pentyl)‐5‐[(*E*)‐(hydroxyimino)methyl]isoquinolin‐2‐ium bromide (4; LG1795):**

# References

[1] Gorecki L, Hepnarova V, Karasova JZ, Hrabinova M, Courageux C, Dias J, Kucera T, Kobrlova T, Muckova L, Prchal L, Malinak D, Jun D, Musilek K, Worek F, Nachon F, Soukup O, Korabecny J, 2021. Development of versatile and potent monoquaternary reactivators of acetylcholinesterase. Arch Toxicol 95, 985–1001. https://doi.org/10.1007/s00204-021-02981-w

[2] Gorecki L, Markova A, Hepnarova V, Zivna N, Junova L, Hrabinova M, Janousek J, Kobrlova T, Prchal L, Jun D, Soukup O, Horn G, Worek F, Marek J, Korabecny J, 2024. Uncharged mono- and bisoximes: In search of a zwitterion to countermeasure organophosphorus intoxication. Chem Biol Interact 394, 110941. https://doi.org/10.1016/j.cbi.2024.110941
